# Improving draft genome contiguity with reference-derived *in silico* mate-pair libraries

**DOI:** 10.1093/gigascience/giy029

**Published:** 2018-04-21

**Authors:** José Horacio Grau, Thomas Hackl, Klaus-Peter Koepfli, Michael Hofreiter

**Affiliations:** 1 Museum für Naturkunde Berlin, Leibniz-Institut für Evolutions- und Biodiversitätsforschung an der Humboldt-Universität zu Berlin. Invalidenstraße 43, 10115. Berlin, Germany; 2 Massachusetts Institute of Technology, Department of Civil and Environmental Engineering, 15 Vassar Street, Cambridge, MA, 02139, USA; 3 Smithsonian Conservation Biology Institute, National Zoological Park, 3001 Connecticut Avenue NW, Washington, D.C. 20008, USA; 4 Theodosius Dobzhansky Center for Genome Bioinformatics, St. Petersburg State University, Sredniy Prospekt 41A, St. Petersburg, 199004, Russia; 5 Faculty of Mathematics and Life Sciences, Institute of Biochemistry and Biology, Unit of General Zoology–Evolutionary Adaptive Genomics, University of Potsdam, Karl-Liebknecht-Straße 24–25, 14476 Potsdam, Germany

**Keywords:** genome assembly, mate-pairs, *in silico*, scaffolding, shotgun sequencing

## Abstract

**Background:**

Contiguous genome assemblies are a highly valued biological resource because of the higher number of completely annotated genes and genomic elements that are usable compared to fragmented draft genomes. Nonetheless, contiguity is difficult to obtain if only low coverage data and/or only distantly related reference genome assemblies are available.

**Findings:**

In order to improve genome contiguity, we have developed Cross-Species Scaffolding—a new pipeline that imports long-range distance information directly into the *de novo* assembly process by constructing mate-pair libraries *in silico*.

**Conclusions:**

We show how genome assembly metrics and gene prediction dramatically improve with our pipeline by assembling two primate genomes solely based on ∼30x coverage of shotgun sequencing data.

## Background

Accurate, complete, and well-annotated genomes provide a wealth of information about the past, present, and future of species and individuals and, therefore, constitute highly valuable resources for medical and biological research [1]. Thanks to the progress in DNA sequencing technology over the past decade, sequencing and assembly of a large variety of genomes from diverse branches of the tree of life have become possible, providing new insights into genomic architecture and phylogeny, as well as the functions of genes, RNAs, and other genomic features. Assemblies with at least near chromosome-level resolution are crucial for understanding genome biology due to the completeness of the information they contain, especially with regard to how loci are ordered and oriented along a chromosome [2]. Therefore, chromosome-level assemblies represent the aspired “gold standard,” but often this standard is hard to reach due to the difficulty of assembling the required long and continuous stretches of DNA [3]. While today more and more genomes are sequenced and assembled to the chromosome level, assemblies of large genomes often remain highly fragmented [4]. Improvement of assembly contiguity is therefore a central issue in genome research. Improved contiguity increases the completeness of genes and genomic elements across the assembly, thereby facilitating better and more complete annotations and downstream analyses. Contiguity, thus, has been proposed as one of the key metrics for evaluating modern assemblies [5, 6].

Despite recent advances in sequencing technologies and genome assembly approaches, obtaining a contiguous assembly of a large genome from short reads remains challenging. For this reason, sequencing technologies that are providing new means for contiguous assembly of large genomes are of great interest to the genomics community. Third-generation long-read sequencing technologies such as PacBio [7] and Nanopore [8], either on their own or in combination with short-read data [9–11], as well as high-quality long-insert clones and single-molecule restriction maps [12], are providing means by which more contiguous genome assemblies can be achieved [13]. However, the advantages of these approaches come at higher costs than simple short-read shotgun sequencing technologies.

Among the largest obstacles for assembling contiguous genomes, especially when using only short-reads, are low-complexity regions and transposable elements [14]; in the case of some chordates and plants, those regions may add up to more than 50% of the total genome size [15]. Repetitive regions complicate and hinder contiguous *de novo* assemblies because the many highly similar copies scattered across the genome lead to a multitude of ambiguous and often unresolvable paths in the underlying assembly graph. As a result, the obtained genome assemblies are fragmented, limiting their use for further analysis.

To increase contiguity, syntenic information may be imported from a closely related species for which a chromosome-level genome assembly is available [16]. While reference-assisted assemblies introduce occasional errors from genome rearrangements and gene duplications, this approach greatly reduces assembly fragmentation and allows better annotation and genomic feature analysis [16, 17]. Although genome assemblies can be further optimized using additional transcriptome [18, 19] or proteome data [20, 21], contiguous assemblies are still difficult to obtain when it comes to large genomes, particularly if only low coverage sequencing data and/or only distantly related reference assemblies are available. Thus, poor contiguity in genome assemblies is a persistent limiting factor in the quest for high-quality genomic references and comprehensively annotated gene repertoires [22].

While paired-end sequencing is usually restricted to insert sizes below 500 bp and thus is ineffective when it comes to re-solving longer repeat regions, mate-pair sequencing can span across several kilobase pairs. Effective use of small, medium, and large insert size mate-pair libraries has provided a dramatic improvement in assembly of large genomes [23, 24]. Several *de novo* genome assemblers today can make use of the long-range information of mate-pairs, and the use of large insert size libraries (20–25 kb) can greatly increase contiguity. Altogether, a more contiguous assembly with larger scaffolds is easily obtained if provided with adequate and sufficient mate-pair information [25]. Generation of mate-pair libraries and third-generation sequencing technologies, however, requires large amounts of high-quality DNA, which can only be obtained from fresh and abundant samples. Furthermore, library preparation and sequencing are much more expensive than for short-read sequencing alone.

## Findings

**Figure 1:**
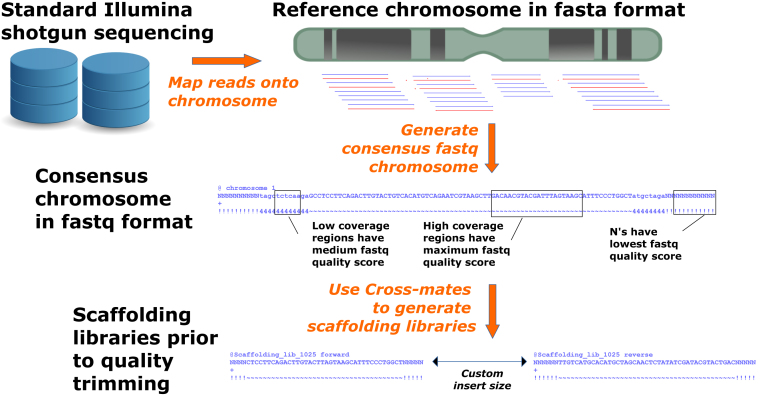
Chart demonstrating the workflow implemented in Cross-Species Scaffolding for generating mate-pair libraries *in silico*. The approach is composed of three steps. In the first step, reads from shotgun libraries are mapped onto a set of repeat-masked reference chromosomes or genome assembly. In the second step, a large consensus fastq file is obtained from every chromosome or contig, generated only from the mapped reads. Finally, Cross-mates is used to simulate the sequencing of mate-pair or paired-end scaffolding libraries from the consensus fastq chromosomes.

To overcome the necessity for long-range sequencing data, which, depending on the project, is either expensive to generate or unobtainable in the first place, we developed a workflow to aid genome assembly that only requires paired-end read data of the query organism and that uses available reference genomes as a basis for generating long-range information by constructing mate-pair or scaffolding libraries *in silico* (Fig. 1). This method has been implemented in a pipeline called Cross-Species Scaffolding.

**Figure 2:**
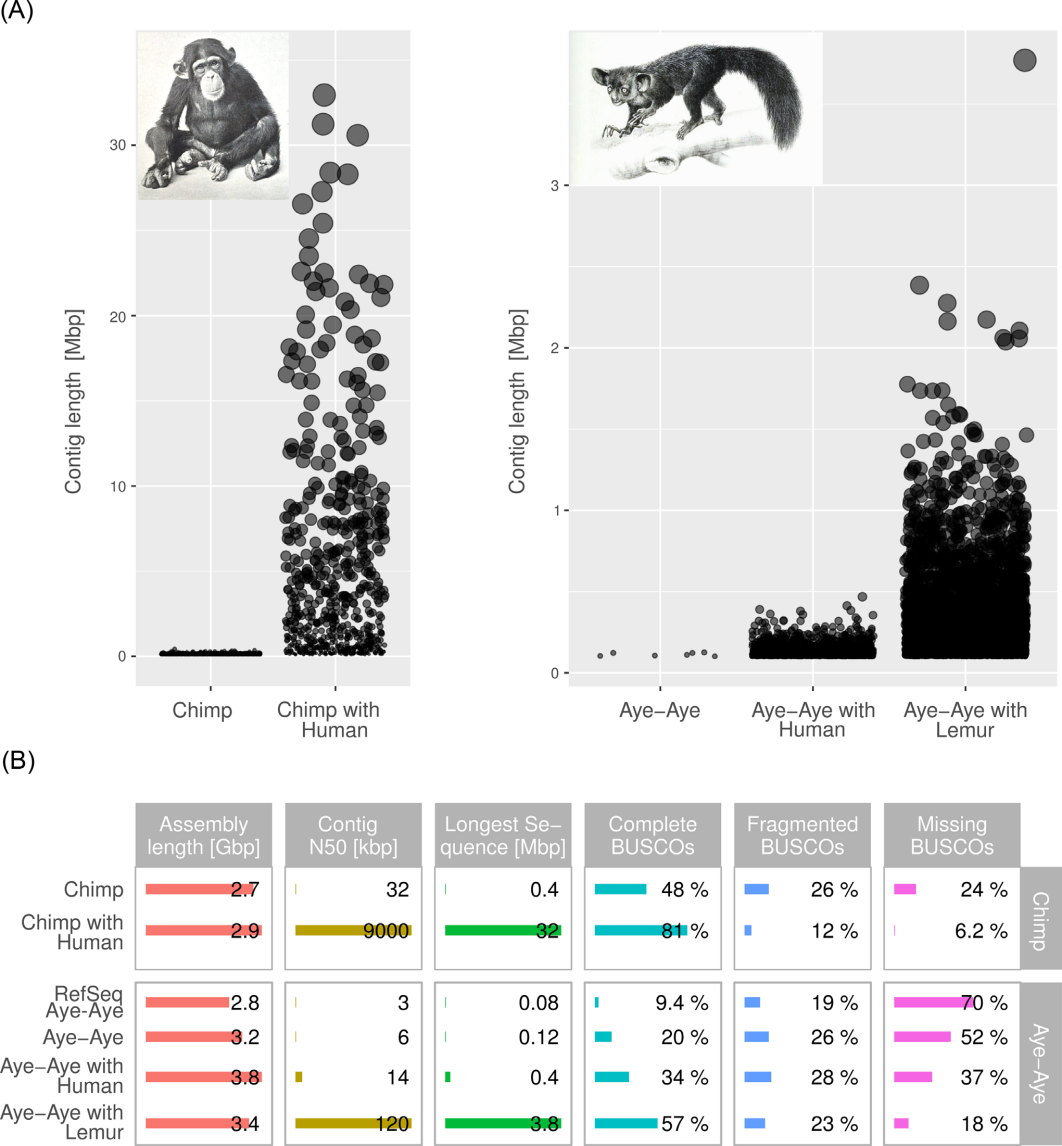
A) Plot of final contig size for the chimpanzee and aye-aye genome assemblies. Chimpanzee genome assembled with shotgun-only data (32x coverage) and with *in silico* mate-pairs generated from the human chromosomes using Cross-mates (see Methods section). Aye-aye genome assembled with shotgun only data (22x coverage) and with *in silico* mate-pairs generated from the human chromosomes and the gray mouse lemur. B) Summary table of the assembly statistics showing chimpanzee and aye-aye results.

To test the efficiency of *in silico* mate-pair libraries for assembling scaffolds, we assembled two genomes based only on standard Illumina shotgun sequencing. In the first assembly experiment, we assembled the chimpanzee genome by generating mate-pair libraries based on the human chromosome set. In the second experiment, we attempted to improve the genome of the aye-aye (*Daubentonia madagascariensis*), a basal nocturnal lemuroid primate with an estimated divergence time from humans of between 70 and 80 million years [26, 27], for which a very fragmented assembly was available. We generated mate-pair libraries using the human chromosome set as reference and a second set using the gray mouse lemur (*Microcebus murinus*) genome, which diverged around 57–59 million years ago (Mya) from the aye-aye [26, 27]. As a quality metric in all assemblies, we used the proportion of 3,023 vertebrate BUSCO (Benchmarking Universal Single-Copy Orthologs) genes that could be correctly and completely annotated. Assemblies were also assessed before and after the use of *in silico* mate-pairs for scaffold size (mean and maximum), number of scaffolds, and scaffold size distribution. While the size of the chimpanzee assembly increases only slightly, the assembly N50 increases by a factor of almost 30 and the length of the longest sequence by a factor of 80, from 400 kbp to 32 Mbp (Fig. 2; Additional file 1: Table S2). A plot of the final contig size shows that 78 contigs >10 Mb in length have been assembled from the short-read shotgun data of the chimpanzee using *in silico* mate-pairs generated from human chromosomes (Fig. 2A). Correspondingly, the gene completeness as measured by BUSCO almost doubles, while the number of fragmented and missing BUSCO genes are reduced by factors of >2 and 4, respectively. The picture is qualitatively similar for the aye-aye assemblies, where the N50 is increased by more than two times and the number of complete BUSCO genes doubles when using the human chromosome set as reference. Moreover, by using the gray mouse lemur as a reference, the N50 of the aye-aye assembly increased by a factor of 20 and the number of complete BUSCO genes nearly tripled (Fig. 2B; Additional file 1: Table S2). Thus, our approach works even when using genomes as references that diverged more than 50 Mya.

In order to time the generation of *in silico* mate-pair libraries, we computed run times based on the human-chimp consensus genome. Run time scales linearly with genome size and target coverage but is largely independent of insert size (Additional file 1: Fig. S1, Table S5). On the customary laptop used for the benchmark, generating 10x coverage of *in silico* mate-pairs takes about 6 seconds per 100 Mbp.

To show that our method is flexible and can be applied across a broad taxonomic spectrum, we also generated experimental assemblies of the pork tapeworm (*Taenia solium*) and of yeast (*Saccharomyces cerevisiae*). In both cases, the assembly N50 showed substantial improvement, with an 80-fold and 11-fold increase for the pork tapeworm and yeast, respectively (Additional file 1: Tables S3 and S4).

Furthermore, to estimate the amount of mis-assemblies, we conducted alignments of all contigs larger than 10 kbp against the reference assemblies for three datasets (yeast, tapeworm, and chimp). As expected, in all three datasets we found a larger amount of mis-assemblies on the assemblies done with *in silico* mate-pairs; nonetheless, in all three datasets, the adjusted N50 size was still nearly 5x larger when *in silico* mate-pairs were used (Additional files 2–4).

## Discussion

We present a simple, yet novel method for incorporating long-range distance information into *de novo* genome assembly from a reference genome through the generation of *in silico* mate-pair or scaffolding libraries. This is an essentially novel approach since other chromosome scaffolders, such as Chromosomer [17], MeDuSa [28], and AlignGraph [29], exploit distance information from a genome of a closely related organism to order and extend scaffold or contigs after the *de novo* assembly process, while *in silico* mate-pair libraries obtain distance information prior to the assembly process and can be adapted to any genome assembler that can take mate-pair sequences as input. Our results show that contiguity and completeness of genome assembly can be greatly improved through the use of *in silico* scaffolding libraries.

While the generation of *in silico* mate-pairs does not introduce errors such as paired-end contamination and chimeras, they cannot fully replace physical mate-pair and third-generation (long reads) sequencing information, as it is probably an inadequate method for studying gene copy number variation, chromosomal structural variation, and synteny.

A drawback of this approach may be the introduction of assembly chimaeras; therefore, special consideration should be given to several factors prior to *in silico* mate-pair generation: (1) quality and quantity (coverage) of shotgun sequencing since the amount of initial data will affect the downstream assembly process. For our experimental assemblies, we considered a minimum of 20–30x coverage of short insert (300–500 bp) paired-end shotgun libraries. Improvement and reduction of mis-assemblies can be expected if higher coverage and longer insert (> 500 bp) shotgun libraries are combined with *in silico* mate-pairs during the assembly. (2) The software chosen for mapping reads to the reference genome. Of the many short-read mappers available, we used BWA [30] with default parameters as a proof of concept. It is likely that mis-assemblies can be further avoided by choosing different mappers with different parameters (e.g., AlignerBoost [31]). (3) As in any genome assembly, a fraction of mis-assemblies can be attributed to the assembly software used. While most genome assemblers produce useful assemblies, there is still a high degree of variability among the assemblies produced by the different genome assemblers [3]; therefore, choosing an adequate assembler for the amount, design, and quality of data available is an important decision. (4) Finally, the phylogenetic distance, quality, and completeness of the reference genome, as well as its overall synteny and transposable element content, will influence the final amount of mis-assemblies. We therefore recommend use of references that are as closely related as possible and to hard mask repetitive regions in the references genomes prior to *in silico* mate-pair generation.

Despite the above-mentioned considerations, *in silico* mate-pair libraries offer several advantages over traditional mate-pair sequencing. First, extra-long-range scaffolding information can be easily obtained, since our tool has no maximum insert size and the upper limit of insert size remains to be explored in relation to syntenic conservation. Thus, it may also prove useful for super-scaffolding already existing scaffolded genome assemblies. Second, another advantage lies in the possibility to generate scaffolding libraries with precise and customized length, orientation, insert size, and coverage from a mapped consensus genome. It is also possible to generate “repetitive element free” scaffolding libraries from hard-masked reference genomes, and reads from phylogenetically distant references may also be used to map onto conserved regions, such as exons. Additionally, because of the consensus calling from the mapped reads, allelic differences will be converted to ambiguous bases in the scaffolding libraries. Third, our method would also allow for consensus libraries to be generated if multiple species/individuals are mapped to the same reference prior to consensus calling of mapped reads. Fourth, it is possible to use more than one reference genome for the generation of *in silico* mate-pair libraries. While this still requires further development and experimentation, we have briefly explored this possibility and successfully assembled a tapeworm genome based on four reference genomes of closely related species (Additional file 1: Table S3). Finally, adaptations of this rationale can be used to generate scaffolding libraries from uncorrected PacBio and Oxford nanopore reads if sufficient Illumina shotgun data are available.

## Conclusions

Overall, *in silico* generated mate-pairs represent a cost-effective strategy for incorporating chromosome-level and large scaffold distance information from related genomes directly into the *de novo* assembly process, requiring only standard Illumina shotgun sequencing data and a suitable reference genome. We have shown that it is even possible to use reference genomes that diverged more than 50 Mya to improve genome quality measures and gene predictions. This is a novel and versatile solution to enrich and improve scaffolding in any genome assembler or chromosome scaffolder that can make use of mate-paired sequences. It is expected that *in silico* generated mate-pairs and scaffolding libraries will become a popular method in the genome assembly community and that substantial improvement of the method will come about through its application.

## Methods

Sequences were downloaded from the National Center for Biotechnology Information (NCBI) SRA (*Daubentonia madagascariensis*: SRP007603; *Pan troglodytes*: SRP012268 [SRX142913]). Raw sequences were preprocessed with Prinseq [32] to remove forward/reverse duplicates and SeqPrep [33] to remove adapters and merge overlapping reads. All preprocessed sequences were passed through *k*mer error correction using BFC [34] specifying the -s parameter for genome size. Multiplicity distribution of 23mers was carried out with Jellyfish2 [35] and KrATER [36] in order to estimate coverage. *De novo* genome assembly was performed with SOAPdenovo2 [37], using the sparse_pregraph module with the following parameters: -g 15 -d 4 -e 4 -R -r 0, and parameter -M 1 during contig phase.

Multiple sets of *in silico* mate-pairs were generated with Cross-mates. First, paired-end reads of the target organism are mapped onto the reference genome with BWA and default settings [38]. Then, a consensus is computed using samtools/bcftools [39] with the samtools legacy variant calling model. Read pairs are sampled from the consensus in systematic mode, i.e., using exact insert sizes and sampling fragments at regularly spaced offsets, skipping regions of coverage lower than three. For the chimpanzee assembly, 14 scaffolding libraries ranging from 500 bp to 200 kb were generated from the human reference at a 10x coverage. For the aye-aye assembly, 16 scaffolding libraries ranging from 500 bp to 20 kb were generated from the human and lemur references, respectively, at a 10x coverage.

Finally, gaps in the assembly were filled in using SOAPdenovo2 GapCloser [37]. Assembly statistics and mis-assemblies were measured with Quast [40]. Completeness and biological accuracy of assembly contiguity were measured by searching for 3,023 vertebrate orthologs as implemented in BUSCO [41] on a set of protein predictions generated by Augustus 3.1.0 [42]. Reference assembly sequences used for generating scaffolding libraries and benchmarking were obtained from NCBI: human (GRCh38.p8; GCF_0 00001405); gray mouse lemur *Microcebus murinus* (Mmur_2.0; GCF_000 165445); aye-aye (DauMad-1.0; GCA_000 241425). All steps used for creating *in silico* scaffolding libraries, including Cross-mates, have been implemented in the pipeline Cross-Species Scaffolding, which is publicly available and maintained at Github. An example of the Cross-mates command line scripts used for the pork tapeworm assembly experiments is included in Additional file 1.

For the pork tapeworm test assembly, *in silico* mate pairs were generated using the reference genomes of four species of tapeworms (*Taenia saginata, T. asiatica, T. multiceps*, and *T. solium*) at a 10x coverage each, with multiple insert sizes ranging from 600 to 50,000 bp and assembled in SOAPdenovo. For the yeast test, we used a different assembler (SPAdes; [43]) for *de novo* assembly with 10x coverage of 500, 2,000, 5,000, and 10,000 bp insert sizes *in silico* mate pairs.

## Availability of supporting data

The datasets generated and/or analyzed are available in the NCBI Short Read Archive repository: SRP012268 [SRX142913] and SRP007603 for the chimpanzee and aye-aye, respectively. Supporting data, including assemblies, BUSCO results, and an archival copy of the code, are available via the *GigaScience* repository, GigaDB [44].

## Availability of supporting source code and requirements

Project name: Cross-species scaffolding

Project home page: https://github.com/thackl/cross-species-scaffolding

Operating system(s): Unix

Programming language: Perl, Bash

Other requirements: Perl v5.10.1 or higher, Bash v4.2 or higher

License: MIT

Research Resource Identifier: Cross-species-scaffolding, RRID:SCR_015932.

## Additional file

Additional file 1: Text S1, Tables S1 to S4, Figure S1.

Additional file 2: QUAST pdf reports for yeast dataset.

Additional file 3: QUAST pdf reports for tapeworm dataset.

Additional file 4: QUAST pdf reports for chimp dataset.

## Abbreviations

BUSCO, Benchmarking Universal Single-Copy Orthologs; Mya, million years ago; NCBI, National Center for Biotechnology Information.

## Competing interests

The authors declare that they have no competing interests.

## Supplementary Material

GIGA-D-17-00092_Original_Submission.pdf

GIGA-D-17-00092_Revision_1.pdf

GIGA-D-17-00092_Revision_2.pdf

GIGA-D-17-00092_Revision_3.pdf

Response_to_Reviewer_Comments_Original_Submission.pdf

Response_to_Reviewer_Comments_Revision_1.pdf

Response_to_Reviewer_Comments_Revision_2.pdf

Reviewer_1_Report_(Original_Submission) -- Kristoffer Sahlin 12 May 2017 Reviewed

Reviewer_1_Report_(Revision_1) -- Kristoffer Sahlin 22 Aug 2017 Reviewed

Reviewer_1_Report_(Revision_2) -- Kristoffer Sahlin 06 Dec 2017 Reviewed

Reviewer_2_Report_(Original_Submission) -- Mohammed-Amin Madoui 15 May 2017 Reviewed

Reviewer_2_Report_(Revision_1) -- Mohammed-Amin Madoui 24 Aug 2017 Reviewed

Additional Files
