# Supplementary material for: Improving draft genome contiguity with reference-derived in silico mate-pair libraries
Source: Gigascience. 2018 Apr 21;7(5):giy029. doi: 10.1093/gigascience/giy029 (PMC5967465; doi:10.1093/gigascience/giy029)
Supplement: GIGA-D-17-00092_Original_Submission.pdf [file giy029_giga-d-17-00092_original_submission.pdf]

GigaScience

Improving draft genome contiguity with reference-derived in silico mate-pair libraries

--Manuscript Draft--

**Manuscript Number:**	GIGA-D-17-00092
**Full Title:**	Improving draft genome contiguity with reference-derived in silico mate-pair libraries
**Article Type:**	Technical Note
**Funding Information:**	European Research Council (310763) — Dr Michael Hofreiter
**Abstract:**	Background. Contiguous genome assemblies are a highly valued biological resource because of the number of completely annotated genes and genomic elements. Nonetheless, contiguity is difficult to obtain if only low coverage data and/or only distantly related reference genome assemblies are available.<br>Findings. In order to improve genome contiguity, we have developed Cross-species scaffolding - a new pipeline which imports long-range distance information directly into the de novo assembly process by constructing mate-pair libraries in silico.<br>Conclusions. We show how genome quality measures and gene prediction dramatically improve with our pipeline by assembling two primate genomes based only on ~30x coverage of shotgun sequencing data.
**Corresponding Author:**	Jose Grau<br>Museum fur Naturkunde - Leibniz-Institut fur Evolutions- und Biodiversitatsforschung<br>Berlin, Berlin GERMANY
**Corresponding Author Secondary Information:**	
**Corresponding Author's Institution:**	Museum fur Naturkunde - Leibniz-Institut fur Evolutions- und Biodiversitatsforschung
**Corresponding Author's Secondary Institution:**	
**First Author:**	Jose Grau
**First Author Secondary Information:**	
**Order of Authors:**	Jose Grau
	Thomas Hackl
	Klaus-Peter Koepfli
	Michael Hofreiter
**Order of Authors Secondary Information:**	
**Opposed Reviewers:**	
**Additional Information:**	
**Question**	**Response**
Are you submitting this manuscript to a special series or article collection?	No
**Experimental design and statistics**<br><br>Full details of the experimental design and statistical methods used should be given in the Methods section, as detailed in our Minimum Standards Reporting Checklist. Information essential to interpreting the data presented should be made available in the figure legends.	Yes

Powered by Editorial Manager® and ProduXion Manager® from Aries Systems Corporation

Have you included all the information requested in your manuscript?	
**Resources**<br><br>A description of all resources used, including antibodies, cell lines, animals and software tools, with enough information to allow them to be uniquely identified, should be included in the Methods section. Authors are strongly encouraged to cite Research Resource Identifiers (RRIDs) for antibodies, model organisms and tools, where possible.<br><br>Have you included the information requested as detailed in our Minimum Standards Reporting Checklist?	Yes
**Availability of data and materials**<br><br>All datasets and code on which the conclusions of the paper rely must be either included in your submission or deposited in publicly available repositories (where available and ethically appropriate), referencing such data using a unique identifier in the references and in the "Availability of Data and Materials" section of your manuscript.<br><br>Have you have met the above requirement as detailed in our Minimum Standards Reporting Checklist?	Yes

Manuscript

Click here to download Manuscript insilicomatepairmanuscriptdraftforGS (1).pdf

Improving draft genome contiguity with reference-derived *in silico* mate-pair libraries

José Horacio Grau [1†], Thomas Hackl [2†], Klaus-Peter Koepfli [3,4], Michael Hofreiter [5].

[1] Museum für Naturkunde Berlin, Leibniz-Institut für Evolutions- und Biodiversitätsforschung an der Humboldt-Universität zu Berlin. Invalidenstraße 43, 10115. Berlin, Germany.

[2] Massachusetts Institute of Technology, Department of Civil and Environmental Engineering, 15 Vassar Street, Cambridge, MA, 02139. USA.

[3] Smithsonian Conservation Biology Institute, National Zoological Park, 3001 Connecticut Avenue NW, Washington, D.C. 20008. USA.

[4] Theodosius Dobzhansky Center for Genome Bioinformatics, St. Petersburg State University, Sredniy Prospekt 41A, St. Petersburg, 199004. Russia.

[5] Faculty of Mathematics and Life Sciences, Institute of Biochemistry and Biology, Unit of General Zoology–Evolutionary Adaptive Genomics, University of Potsdam, Karl-Liebknecht-Straße 24-25, 14476 Potsdam, Germany.

[†] Authors contributed equally

Corresponding author:

José Horacio Grau

jose.grau@mfn-berlin.de

ABSTRACT

Background. Contiguous genome assemblies are a highly valued biological resource because of the number of completely annotated genes and genomic elements that are usable compared to fragmented draft genomes. Nonetheless, contiguity is difficult to obtain if only low coverage data and/or only distantly related reference genome assemblies are available.

Findings. In order to improve genome contiguity, we have developed Cross-species scaffolding - a new pipeline which imports long-range distance information directly into the *de novo* assembly process by constructing mate-pair libraries *in silico*.

Conclusions. We show how genome quality measures and gene prediction dramatically improve with our pipeline by assembling two primate genomes solely based on ~30x coverage of shotgun sequencing data.

KEYWORDS

Genome assembly, mate-pairs, in silico, scaffolding, shotgun sequencing

BACKGROUND

Accurate, complete and well-annotated genomes provide a wealth of information about the past, present and future of species and individuals, and therefore, constitute highly valuable resources for medical and biological research [1]. Thanks to the progress in DNA sequencing technology over the past decade, sequencing and assembly of a large variety of genomes from diverse branches of the tree of life has become possible, providing new insights into genomic architecture and phylogeny, as well as the functions of genes, RNAs, and other genomic features. Assemblies with at least near chromosome-level resolution are crucial for understanding genome biology due to the completeness of the information they contain, especially with regards to how loci are ordered and oriented along a chromosome [2]. Therefore, chromosome-level assemblies represent the aspired “gold standard”, but this standard often is hard to reach due to the difficulty of assembling the required long and continuous stretches of DNA [3]. While today more and more genomes are sequenced and assembled to chromosome level, assemblies of large genomes often remain highly fragmented [4]. Improvement of assembly contiguity is therefore a central issue in genome research: Improved contiguity increases the completeness of genes and genomic elements across the assembly, thereby facilitating better and more complete annotations and downstream analyses.

Contiguity, thus, has been proposed as one of the key metrics for evaluating modern assemblies [5,6].

Despite recent advances in sequencing technologies and genome assembly approaches, obtaining a contiguous assembly of a large genome from short-reads remains challenging. For this reason, sequencing technologies that are providing new means for contiguous assembly of large genomes are of great interest to the genomics community. Third generation long-read sequencing technologies such as PacBio [7] and Nanopore [8], either on their own or in combination with short-read data [9–11], as well as high quality long-insert clones and single-molecule restriction maps [12], are providing means by which more contiguous genome assemblies can be achieved [13]. However, the advantages of these approaches come at higher costs than simple short-read shotgun sequencing technologies.

Among the largest obstacles for assembling contiguous genomes, especially when using only short-reads, are low complexity regions and transposable elements [14]; in the case of some chordates and plants those regions may add up to over 50% of the total genome size [15]. Repetitive regions complicate and hinder contiguous *de novo* assemblies because the many highly similar copies scattered across the genome lead to a multitude of ambiguous, and often unresolvable paths in the underlying assembly graph. As a result, the obtained genome assemblies are fragmented, limiting their use for further analysis.

To increase contiguity, syntenic information may be imported from a closely related species for which a chromosome-level genome assembly is available [16]. While reference-assisted assemblies introduce occasional errors from genome rearrangements and gene duplications, this approach greatly reduces assembly fragmentation and allows better annotation and genomic feature analysis [16,17]. Although genome assemblies can be further optimized using additional transcriptome [18,19] or proteome data [20,21], contiguous assemblies are still difficult to obtain when it comes to large genomes, particularly if only low coverage sequencing data and/or only distantly related reference assemblies are available. Thus, poor contiguity in genome assemblies is a persistent limiting factor in the quest for high-quality genomic references and comprehensively annotated gene repertoires [22].

While paired-end sequencing is usually restricted to insert sizes below 500 bp and thus ineffective when it comes to resolving longer repeat regions, mate-pair sequencing can span across several kilobase pairs. Effective use of small, medium and large insert size mate-pair libraries has provided a dramatic improvement in assembly of large genomes [23,24]. Several *de novo* genome assemblers today can make use of the long-range information of mate-pairs,

and the use of large insert size libraries (20-25 kb) can greatly increase contiguity. Altogether, a more contiguous assembly with larger scaffolds is easily obtained if provided with adequate and sufficient mate-pair information [25]. Generation of mate-pair libraries and third-generation sequencing technologies, however, requires large amounts of high quality DNA, which can only be obtained from fresh and abundant samples. Furthermore, library preparation and sequencing are much more expensive than for short-read sequencing alone.

FINDINGS

To overcome the necessity for long-range sequencing data, which, depending on the project, is either expensive to generate or unobtainable in the first place, we developed a new assembly workflow, which only requires paired-end read data of the query organism, and which utilizes available reference genomes as a basis for generating long-range information by constructing mate-pair or scaffolding libraries *in silico* (Figure 1). This method has been implemented in a pipeline called Cross-Species Scaffolding.

To test the efficiency of *in silico* mate-pair libraries for assembling contigs, we assembled two genomes based only on standard Illumina shotgun sequencing. In the first assembly experiment, we assembled the chimpanzee genome by generating mate-pair libraries based on the human chromosome set. In the second experiment, we attempted to improve the genome of the Aye-aye (*Daubentonia madagascariensis*), a basal nocturnal lemuroid primate with an estimated divergence time from humans between 70 and 80 million years [26,27], for which a very fragmented assembly was available. We generated mate-pair libraries using the human chromosome set as reference, and a second set using the Gray mouse lemur (*Microcebus murinus*) genome, which diverged around 57-59 mya from the Aye-aye [26,27]. As a quality metric in all assemblies, we have used the proportion of 3,023 vertebrate BUSCO (Benchmarking Universal Single-Copy Orthologs) genes that could be correctly and completely annotated. Assemblies were also assessed before and after the use of *in silico* mate-pairs for scaffold size (mean and maximum), number of scaffolds and scaffold size distribution. While the size of the chimp assembly increases only slightly, the assembly N50 increases by a factor of almost 30 and the length of the longest sequence by a factor of 80, from 400 kbp to 32 Mbp (Figure 2). A plot of the final contig size shows that 78 contigs >10 Mb in length have been assembled from the short read shotgun data of the Chimpanzee using *in silico* mate-pairs generated from Human chromosomes (Figure 2A). Correspondingly, the gene completeness as measured by BUSCO almost doubles, while the number of fragmented and missing BUSCO

genes are reduced by factors of >2 and 4, respectively. The picture is qualitatively similar for the Aye-aye assemblies, where the N50 is increased by more than two times and the number of complete BUSCO genes doubles when using the human chromosome set as reference. Moreover, by using the Gray mouse lemur as reference, the N50 of the Aye-aye assembly increased by a factor of 20 and the number of complete BUSCO genes nearly triples (Figure 2B). Thus, our approach works even when using genomes as references that diverged more than 50 mya.

Discussion.

We present a simple, yet novel method for incorporating long-range distance information into *de novo* genome assembly from a reference genome through the generation of *in silico* mate-pair or scaffolding libraries. This is an essentially novel approach since other chromosome scaffolders, such as Chromosomer [17], MeDuSa [28], and AlignGraph [29], exploit distance information from genomes of closely related organisms to order and extend scaffold or contigs after the *de novo* assembly process. Our results show that contiguity and completeness of genome assembly can be greatly improved through the use of *in silico* scaffolding libraries. While *in silico* mate-pairs introduces minimal errors since position and arrangement is suggested and validated by shotgun data during the *de novo* assembly process, they cannot fully replace physical mate-pair and third generation (long reads) sequencing information as it is probably an inadequate method for studying gene copy number variation, chromosomal structural variation and synteny.

Nonetheless, *in silico* mate-pair libraries offer several advantages over traditional mate-pair sequencing. First, extra-long-range scaffolding information can be easily obtained, since our tool has no maximum insert size and the upper limit of insert size remains to be explored in relation to syntenic conservation. Thus, it may also prove useful for super-scaffolding already existing scaffolded genome assemblies. Second, another advantage lies in the possibility to generate scaffolding libraries with precise and customized length, orientation, insert size and coverage from a mapped consensus genome. It is also possible to generate “repetitive element free” scaffolding libraries from hard-masked reference genomes, and reads from phylogenetically distant references may also be used to map onto conserved regions, such as exons. Additionally, because of the consensus calling from the mapped reads, allelic differences will be converted to ambiguous bases in the scaffolding libraries. Third, our method would also allow for consensus libraries to be generated if multiple species/individuals

are mapped to the same reference prior to consensus calling of mapped reads. Furthermore, adaptations of this rationale can be used to generate scaffolding libraries from uncorrected PacBio and Oxford nanopore reads if sufficient Illumina shotgun data is available.

Conclusions.

Overall, *in silico* generated mate-pairs represent a cost-effective strategy for incorporating chromosome-level and large scaffold distance information from related genomes directly into the *de novo* assembly process, requiring only standard Illumina shotgun sequencing data and a suitable reference genome. We have shown that it is even possible to use reference genomes that diverged more than 50 million years ago to improve genome quality measures and gene predictions. This is an essentially novel and versatile solution to enrich and improve scaffolding in any genome assembler or chromosome scaffolder that can make use of mate-paired sequences. It is expected that *in silico* generated mate-pairs and scaffolding libraries will become a popular method in the genome assembly community, and that substantial improvement of the method will come about through its application.

METHODS

Sequences were downloaded from the NCBI SRA (*Daubentonia madagascariensis*: SRP007603; *Pan troglodytes*: SRP012268 [SRX142913]). Raw sequences were preprocessed with Prinseq [30] to remove forward/reverse duplicates and SeqPrep (https://github.com/jstjohn/SeqPrep) to remove adapters and merge overlapping reads. All preprocessed sequences were passed through *k*mer error correction using BFC [31] specifying the -s parameter for genome size. Multiplicity distribution of 23mers was carried out with Jellyfish2 [32] and KrATER (https://github.com/mahajrod/KrATER) in order to estimate coverage. *De novo* genome assembly was performed with SOAPdenovo2 [33], using the sparse_pregraph module with the following parameters: -g 15 -d 4 -e 4 -R -r 0, and parameter -M 1 during contig phase. Multiple sets of *in silico* mate-pairs were generated with the program Cross-mates using different insert sizes and length. For the Chimp assembly, 14 scaffolding libraries ranging from 500bp to 200kb were generated from the Human reference. For the Aye-aye assembly, 16 scaffolding libraries ranging from 500bp to 20kb were generated from the human and lemur references, respectively. Finally, gaps in the assembly were filled-in using GapCloser (http://soap.genomics.org.cn). Assembly quality statistics were measured with Quast

[34]. Completeness and biological accuracy of assembly contiguity was measured by searching for 3,023 vertebrate orthologs as implemented in BUSCO [35] on a set of protein predictions generated by Augustus 3.1.0 [36]. Reference assembly sequences used for generating scaffolding libraries were obtained from NCBI: Human (GRCh38.p8; GCF_000001405); and Gray mouse lemur *Microcebus murinus* (Mmur_2.0; GCF_000165445).

All steps used for creating *in silico* scaffolding libraries, including Cross-mates, have been implemented in the pipeline Cross-Species Scaffolding, which is publicly available and maintained at Github (https://github.com/thackl/cross-species-scaffolding).

AVAILABILITY OF SUPPORTING SOURCE CODE AND REQUIREMENTS

Project name: Cross-species scaffolding

Project home page: https://github.com/thackl/cross-species-scaffolding

Operating system(s): Unix

Programming language: Perl, Bash

Other requirements: Perl v5.10.1 or higher, Bash v4.2 or higher

License: MIT

DECLARATIONS

List of Abbreviations.

BUSCO (Benchmarking Universal Single-Copy Orthologs).

Ethics approval and consent to participate.

Not applicable.

Availability of data and material.

The datasets generated and/or analysed during the current study are available in the NCBI Short Read Archive repository: https://www.ncbi.nlm.nih.gov/sra/SRX142913 and https://www.ncbi.nlm.nih.gov/sra/SRP007603 for the Chimp and Aye-Aye, respectively.

Consent for publication.

Not applicable.

Competing interests.

The authors declare that they have no competing interests.

Funding.

This work was supported by European Research Council (consolidator grant 310763 GeneFlow to M.H.).

Authors' contributions.

JHG and TH conceived and designed the study, and developed the main pipeline of the method. KPK and MH made substantial intellectual contributions and actively participated in drafting, revising, and improving the manuscript and method. All authors read and approved the final manuscript.

REFERENCES

1. Ekblom R, Wolf JBW. A field guide to whole-genome sequencing, assembly and annotation. Evol. Appl. 2014;7:1026–42.

2. Damas J, O'Connor R, Farré M, Lenis VPE, Martell HJ, Mandawala A, et al. Upgrading short read animal genome assemblies to chromosome level using comparative genomics and a universal probe set. Genome Res. 2016; Available from: http://dx.doi.org/10.1101/gr.213660.116

3. Bradnam KR, Fass JN, Alexandrov A, Baranay P, Bechner M, Birol I, et al. Assemblathon 2: evaluating de novo methods of genome assembly in three vertebrate species. Gigascience. 2013;2:10.

4. Baker M. De novo genome assembly: what every biologist should know. Nat. Methods. 2012;9:333–7.

5. Koepfli K-P, Paten B, Genome 10K Community of Scientists, O'Brien SJ. The Genome 10K

Project: a way forward. Annu Rev Anim Biosci. 2015;3:57–111.

6. Lee H, Gurtowski J, Yoo S, Nattestad M, Marcus S, Goodwin S, et al. Third-generation sequencing and the future of genomics. bioRxiv. 2016; 048603; doi: https://doi.org/10.1101/048603

7. Rhoads A, Au KF. PacBio Sequencing and Its Applications. Genomics Proteomics Bioinformatics. 2015;13:278–89.

8. Mikheyev AS, Tin MMY. A first look at the Oxford Nanopore MinION sequencer. Mol. Ecol. Resour. 2014;14:1097–102.

9. Hackl T, Hedrich R, Schultz J, Förster F. proovread: large-scale high-accuracy PacBio correction through iterative short read consensus. Bioinformatics. 2014;30:3004–11.

10. Lin H-H, Liao Y-C. Evaluation and Validation of Assembling Corrected PacBio Long Reads for Microbial Genome Completion via Hybrid Approaches. PLoS One. 2015;10:e0144305.

11. Antipov D, Korobeynikov A, McLean JS, Pevzner PA. hybridSPAdes: an algorithm for hybrid assembly of short and long reads. Bioinformatics. 2016;32:1009–15.

12. Howe K, Wood JMD. Using optical mapping data for the improvement of vertebrate genome assemblies. Gigascience; 2015;4:10.

13. Vij S, Kuhl H, Kuznetsova IS, Komissarov A, Yurchenko AA, Van Heusden P, et al. Chromosomal-Level Assembly of the Asian Seabass Genome Using Long Sequence Reads and Multi-layered Scaffolding. PLoS Genet. 2016;12:e1005954.

14. Salzberg SL, Yorke JA. Beware of mis-assembled genomes. Bioinformatics. 2005;21:4320–1.

15. Elliott TA, Gregory TR. Do larger genomes contain more diverse transposable elements? BMC Evol. Biol. 2015;15:69.

16. Kim J, Larkin DM, Cai Q, Asan, Zhang Y, Ge R-L, et al. Reference-assisted chromosome assembly. Proc. Natl. Acad. Sci. 2013;110:1785–90.

17. Tamazian G, Dobrynin P, Krasheninnikova K, Komissarov A, Koepfli K-P, O’Brien SJ.

Chromosomer: a reference-based genome arrangement tool for producing draft chromosome sequences. Gigascience. 2016;5:38.

18. Zhang SV, Zhuo L, Hahn MW. AGOUTI: improving genome assembly and annotation using transcriptome data. Gigascience. 2016;5:31.

19. Song L, Shankar DS, Florea L. Rascaf: Improving Genome Assembly with RNA Sequencing Data. Plant Genome. 2016;9. Available from: http://dx.doi.org/10.3835/plantgenome2016.03.0027

20. Li YI, Copley RR. Scaffolding low quality genomes using orthologous protein sequences. Bioinformatics. 2013;29:160–5.

21. Zhu B-H, Song Y-N, Xue W, Xu G-C, Xiao J, Sun M-Y, et al. PEP_scaffolder: using (homologous) proteins to scaffold genomes. Bioinformatics. 2016;32:3193–5.

22. Salzberg SL, Phillippy AM, Zimin A, Puiu D, Magoc T, Koren S, et al. GAGE: A critical evaluation of genome assemblies and assembly algorithms. Genome Res. 2012;22:557–67.

23. Wetzel J, Kingsford C, Pop M. Assessing the benefits of using mate-pairs to resolve repeats in de novo short-read prokaryotic assemblies. BMC Bioinformatics. 2011;12:95.

24. van Heesch S, Kloosterman WP, Lansu N, Ruzius F-P, Levandowsky E, Lee CC, et al. Improving mammalian genome scaffolding using large insert mate-pair next-generation sequencing. BMC Genomics. 2013;14:257.

25. Lin H. Theoretical Bounds on Mate-Pair Information for Accurate Genome Assembly. arXiv. 2013. http://arxiv.org/abs/1310.1653. Accessed 24 Apr 2017.

26. Finstermeier K, Zinner D, Brameier M, Meyer M, Kreuz E, Hofreiter M, et al. A Mitogenomic Phylogeny of Living Primates. PLoS One. 2013;8:e69504.

27. Perelman P, Johnson WE, Roos C, Seuánez HN, Horvath JE, Moreira MAM, et al. A Molecular Phylogeny of Living Primates. PLoS Genet. 2011;7:e1001342.

28. Bosi E, Donati B, Galardini M, Brunetti S, Sagot M-F, Lió P, et al. MeDuSa: a multi-draft based scaffolder. Bioinformatics. 2015;31:2443–51.

29. Bao E, Jiang T, Girke T. AlignGraph: algorithm for secondary de novo genome assembly guided by closely related references. Bioinformatics. 2014;30:i319–28.

30. Schmieder R, Edwards R. Quality control and preprocessing of metagenomic datasets. Bioinformatics. 2011;27:863–4.

31. Li H. BFC: correcting Illumina sequencing errors. Bioinformatics. 2015;31:2885–7.

32. Marçais G, Kingsford C. A fast, lock-free approach for efficient parallel counting of occurrences of k-mers. Bioinformatics. 2011;27:764–70.

33. Luo R, Liu B, Xie Y, Li Z, Huang W, Yuan J, et al. SOAPdenovo2: an empirically improved memory-efficient short-read de novo assembler. Gigascience. 2012;1:18.

34. Gurevich A, Saveliev V, Vyahhi N, Tesler G. QUAST: quality assessment tool for genome assemblies. Bioinformatics. 2013;29:1072–5.

35. Simão FA, Waterhouse RM, Ioannidis P, Kriventseva EV, Zdobnov EM. BUSCO: assessing genome assembly and annotation completeness with single-copy orthologs. Bioinformatics. 2015;31:3210–2.

36. Stanke M, Keller O, Gunduz I, Hayes A, Waack S, Morgenstern B. AUGUSTUS: ab initio prediction of alternative transcripts. Nucleic Acids Res. 2006;34:W435–9.

FIGURE CAPTIONS

Figure 1. Chart demonstrating the workflow implemented in Cross-Species Scaffolding for generating mate-pair libraries *in silico*. The approach is composed of three steps. In the first step, reads from shotgun libraries are mapped onto a set of repeat-masked reference chromosomes or genome assembly. In the second step, a large consensus fastq file is obtained from every chromosome or contig, generated only from the mapped reads. And finally,

Cross-mates is used to simulate the sequencing of mate-pair or paired-end scaffolding libraries from the consensus fastq chromosomes.

Figure 2. A) Plot of final contig size for the Chimp and Aye-Aye genome assemblies. Chimp genome assembled with shotgun only data (32x coverage) and with *in silico* mate-pairs generated from the Human chromosomes using Cross-mates (see Materials and Methods). Aye-Aye genome assembled with shotgun only data (22x coverage) and with *in silico* mate-pairs generated from the Human chromosomes and the Gray mouse lemur. B) Summary table of the assembly statistics showing Chimp and Aye-Aye results.

Figure 1

Click here to download Figure crossmates.svg.pdf

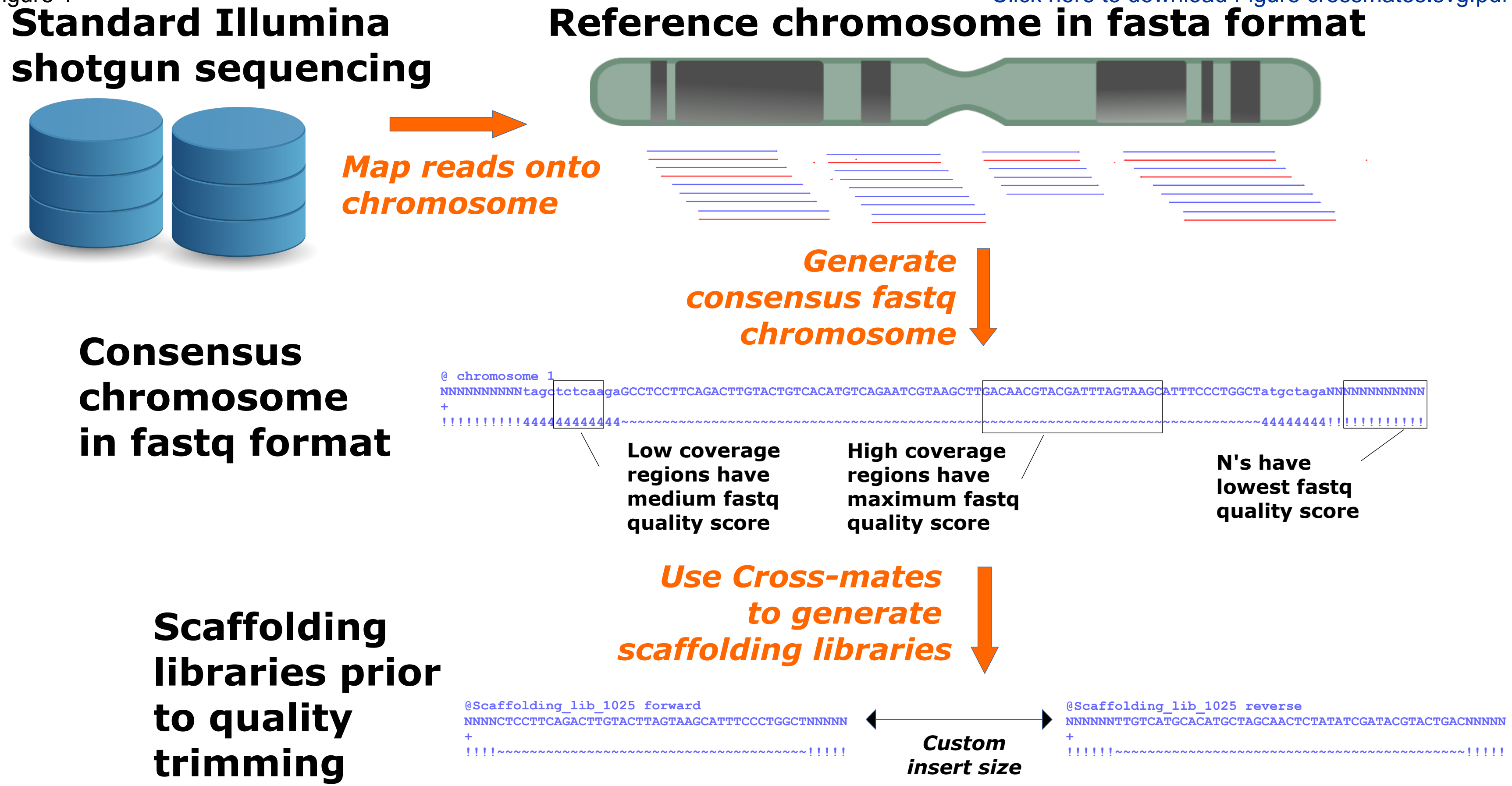

Figure 2

Click here to download Figure mates_drawing.svg.pdf

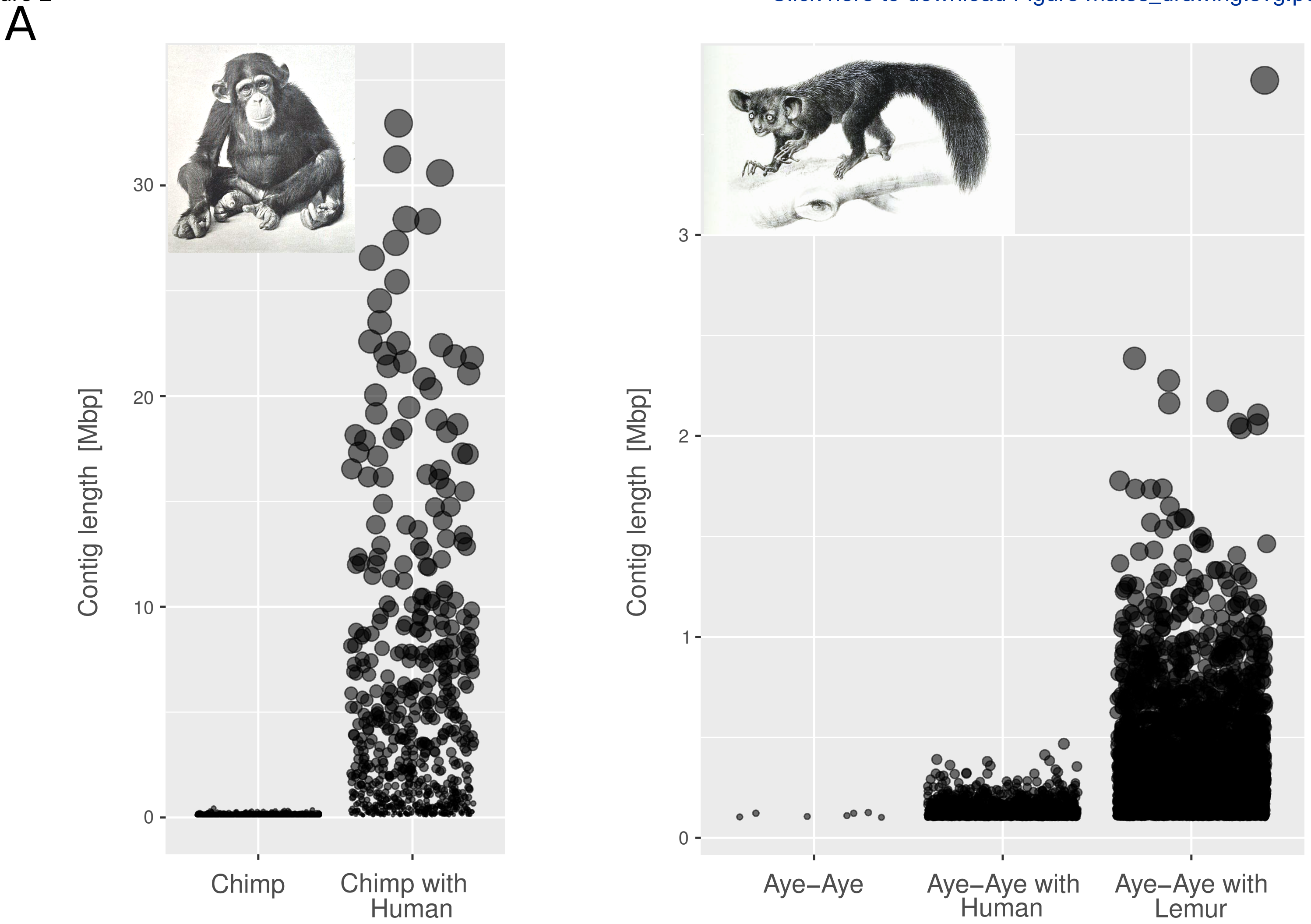

B

		Assembly length [Gbp]	Contig N50 [kbp]	Longest Sequence [Mbp]	Complete BUSCOs	Fragmented BUSCOs	Missing BUSCOs
Chimp	Chimp	2.7	32	0.4	48 %	26 %	24 %
Chimp	Chimp with Human	2.9	9000	32	81 %	12 %	6.2 %
Aye–Aye	DauMad_1.0	2.8	3	0.08	9.4 %	19 %	70 %
Aye–Aye	Aye–Aye	3.2	6	0.12	20 %	26 %	52 %
Aye–Aye	Aye–Aye with Human	3.8	14	0.4	34 %	28 %	37 %
Aye–Aye	Aye–Aye with Lemur	3.4	120	3.8	57 %	23 %	18 %
