# Supplementary material for: Improving draft genome contiguity with reference-derived in silico mate-pair libraries
Source: Gigascience. 2018 Apr 21;7(5):giy029. doi: 10.1093/gigascience/giy029 (PMC5967465; doi:10.1093/gigascience/giy029)
Supplement: GIGA-D-17-00092_Revision_1.pdf [file giy029_giga-d-17-00092_revision_1.pdf]

GigaScience

Improving draft genome contiguity with reference-derived in silico mate-pair libraries

--Manuscript Draft--

**Manuscript Number:**	GIGA-D-17-00092R1
**Full Title:**	Improving draft genome contiguity with reference-derived in silico mate-pair libraries
**Article Type:**	Technical Note
**Funding Information:**	European Research Council (310763) — Dr Michael Hofreiter
**Abstract:**	Background. Contiguous genome assemblies are a highly valued biological resource because of the higher number of completely annotated genes and genomic elements that are usable compared to fragmented draft genomes. Nonetheless, contiguity is difficult to obtain if only low coverage data and/or only distantly related reference genome assemblies are available.<br>Findings. In order to improve genome contiguity, we have developed Cross-Species Scaffolding - a new pipeline which imports long-range distance information directly into the de novo assembly process by constructing mate-pair libraries in silico.<br>Conclusions. We show how genome assembly metrics and gene prediction dramatically improve with our pipeline by assembling two primate genomes solely based on ~30x coverage of shotgun sequencing data.
**Corresponding Author:**	Jose Grau<br>Museum fur Naturkunde - Leibniz-Institut fur Evolutions- und Biodiversitatsforschung<br>Berlin, Berlin GERMANY
**Corresponding Author Secondary Information:**	
**Corresponding Author's Institution:**	Museum fur Naturkunde - Leibniz-Institut fur Evolutions- und Biodiversitatsforschung
**Corresponding Author's Secondary Institution:**	
**First Author:**	Jose Grau
**First Author Secondary Information:**	
**Order of Authors:**	Jose Grau
	Thomas Hackl
	Klaus-Peter Koepfli
	Michael Hofreiter
**Order of Authors Secondary Information:**	
**Response to Reviewers:**	Please find our comments in the attached PDF.
**Additional Information:**	
**Question**	**Response**
Are you submitting this manuscript to a special series or article collection?	No
**Experimental design and statistics**<br><br>Full details of the experimental design and statistical methods used should be given in the Methods section, as detailed in our Minimum Standards Reporting Checklist. Information essential to interpreting the data presented should be made available in the figure legends.	Yes

Powered by Editorial Manager® and ProduXion Manager® from Aries Systems Corporation

Have you included all the information requested in your manuscript?	
**Resources**<br><br>A description of all resources used, including antibodies, cell lines, animals and software tools, with enough information to allow them to be uniquely identified, should be included in the Methods section. Authors are strongly encouraged to cite Research Resource Identifiers (RRIDs) for antibodies, model organisms and tools, where possible.<br><br>Have you included the information requested as detailed in our Minimum Standards Reporting Checklist?	Yes
**Availability of data and materials**<br><br>All datasets and code on which the conclusions of the paper rely must be either included in your submission or deposited in publicly available repositories (where available and ethically appropriate), referencing such data using a unique identifier in the references and in the "Availability of Data and Materials" section of your manuscript.<br><br>Have you have met the above requirement as detailed in our Minimum Standards Reporting Checklist?	Yes

Powered by Editorial Manager® and ProduXion Manager® from Aries Systems Corporation

Manuscript
Click here to download Manuscript insilico mate pair manuscript draft for GS2.pdf

Improving draft genome contiguity with reference-derived *in silico* mate-pair libraries

José Horacio Grau [1†], Thomas Hackl [2†], Klaus-Peter Koepfli [3,4], Michael Hofreiter [5].

[1] Museum für Naturkunde Berlin, Leibniz-Institut für Evolutions- und Biodiversitätsforschung an der Humboldt-Universität zu Berlin. Invalidenstraße 43, 10115. Berlin, Germany.

[2] Massachusetts Institute of Technology, Department of Civil and Environmental Engineering, 15 Vassar Street, Cambridge, MA, 02139. USA.

[3] Smithsonian Conservation Biology Institute, National Zoological Park, 3001 Connecticut Avenue NW, Washington, D.C. 20008. USA.

[4] Theodosius Dobzhansky Center for Genome Bioinformatics, St. Petersburg State University, Sredniy Prospekt 41A, St. Petersburg, 199004. Russia.

[5] Faculty of Mathematics and Life Sciences, Institute of Biochemistry and Biology, Unit of General Zoology–Evolutionary Adaptive Genomics, University of Potsdam, Karl-Liebknecht-Straße 24-25, 14476 Potsdam, Germany.

[†] Authors contributed equally

Corresponding author:

José Horacio Grau

jose.grau@mfn-berlin.de

ABSTRACT

Background. Contiguous genome assemblies are a highly valued biological resource because of the higher number of completely annotated genes and genomic elements that are usable compared to fragmented draft genomes. Nonetheless, contiguity is difficult to obtain if only low coverage data and/or only distantly related reference genome assemblies are available.
Findings. In order to improve genome contiguity, we have developed Cross-Species Scaffolding - a new pipeline which imports long-range distance information directly into the *de novo* assembly process by constructing mate-pair libraries *in silico*.
Conclusions. We show how genome assembly metrics and gene prediction dramatically improve with our pipeline by assembling two primate genomes solely based on ~30x coverage of shotgun sequencing data.

KEYWORDS

Genome assembly, mate-pairs, in silico, scaffolding, shotgun sequencing

BACKGROUND

Accurate, complete and well-annotated genomes provide a wealth of information about the past, present and future of species and individuals, and therefore, constitute highly valuable resources for medical and biological research [1]. Thanks to the progress in DNA sequencing technology over the past decade, sequencing and assembly of a large variety of genomes from diverse branches of the tree of life has become possible, providing new insights into genomic architecture and phylogeny, as well as the functions of genes, RNAs, and other genomic features. Assemblies with at least near chromosome-level resolution are crucial for understanding genome biology due to the completeness of the information they contain, especially with regards to how loci are ordered and oriented along a chromosome [2]. Therefore, chromosome-level assemblies represent the aspired “gold standard”, but often this standard is hard to reach due to the difficulty of assembling the required long and continuous stretches of DNA [3]. While today more and more genomes are sequenced and assembled to chromosome level, assemblies of large genomes often remain highly fragmented [4]. Improvement of assembly contiguity is therefore a central issue in genome research: Improved contiguity increases the completeness of genes and genomic elements across the assembly, thereby facilitating better and more complete annotations and downstream analyses. Contiguity, thus, has been proposed as one of the key metrics for evaluating modern assemblies [5,6].

Despite recent advances in sequencing technologies and genome assembly approaches, obtaining a contiguous assembly of a large genome from short reads remains challenging. For this reason, sequencing technologies that are providing new means for contiguous assembly of large genomes are of great interest to the genomics community. Third generation long-read sequencing technologies such as PacBio [7] and Nanopore [8], either on their own or in combination with short-read data [9–11], as well as high quality long-insert clones and single-molecule restriction maps [12], are providing means by which more contiguous genome assemblies can be achieved [13]. However, the advantages of these approaches come at higher costs than simple short-read shotgun sequencing technologies.

Among the largest obstacles for assembling contiguous genomes, especially when using only short-reads, are low complexity regions and transposable elements [14]; in the case of some chordates and plants those regions may add up to over 50% of the total genome size [15]. Repetitive regions complicate and hinder contiguous *de novo* assemblies because the many highly similar copies scattered across the genome lead to a multitude of ambiguous, and often unresolvable paths in the underlying assembly graph. As a result, the obtained genome assemblies are fragmented, limiting their use for further analysis.

To increase contiguity, syntenic information may be imported from a closely related species for which a chromosome-level genome assembly is available [16]. While reference-assisted assemblies introduce occasional errors from genome rearrangements and gene duplications, this approach greatly reduces assembly fragmentation and allows better annotation and genomic feature analysis [16,17]. Although genome assemblies can be further optimized using additional transcriptome [18,19] or proteome data [20,21], contiguous assemblies are still difficult to obtain when it comes to large genomes, particularly if only low coverage sequencing data and/or only distantly related reference assemblies are available. Thus, poor contiguity in genome assemblies is a persistent limiting factor in the quest for high-quality genomic references and comprehensively annotated gene repertoires [22].

While paired-end sequencing is usually restricted to insert sizes below 500 bp and thus ineffective when it comes to resolving longer repeat regions, mate-pair sequencing can span across several kilobase pairs. Effective use of small, medium and large insert size mate-pair libraries has provided a dramatic improvement in assembly of large genomes [23,24]. Several *de novo* genome assemblers today can make use of the long-range information of mate-pairs, and the use of large insert size libraries (20-25 kb) can greatly increase contiguity. Altogether, a more contiguous assembly with larger scaffolds is easily obtained if provided with adequate and

sufficient mate-pair information [25]. Generation of mate-pair libraries and third-generation sequencing technologies, however, requires large amounts of high quality DNA, which can only be obtained from fresh and abundant samples. Furthermore, library preparation and sequencing are much more expensive than for short-read sequencing alone.

FINDINGS

To overcome the necessity for long-range sequencing data, which, depending on the project, is either expensive to generate or unobtainable in the first place, we developed a workflow to aid genome assembly, which only requires paired-end read data of the query organism, and which utilizes available reference genomes as a basis for generating long-range information by constructing mate-pair or scaffolding libraries *in silico* (Figure 1). This method has been implemented in a pipeline called Cross-Species Scaffolding.

To test the efficiency of *in silico* mate-pair libraries for assembling scaffolds, we assembled two genomes based only on standard Illumina shotgun sequencing. In the first assembly experiment, we assembled the chimpanzee genome by generating mate-pair libraries based on the human chromosome set. In the second experiment, we attempted to improve the genome of the aye-aye (*Daubentonia madagascariensis*), a basal nocturnal lemuroid primate with an estimated divergence time from humans between 70 and 80 million years [26,27], for which a very fragmented assembly was available. We generated mate-pair libraries using the human chromosome set as reference, and a second set using the gray mouse lemur (*Microcebus murinus*) genome, which diverged around 57-59 mya from the aye-aye [26,27]. As a quality metric in all assemblies, we have used the proportion of 3,023 vertebrate BUSCO (Benchmarking Universal Single-Copy Orthologs) genes that could be correctly and completely annotated. Assemblies were also assessed before and after the use of *in silico* mate-pairs for scaffold size (mean and maximum), number of scaffolds and scaffold size distribution. While the size of the chimpanzee assembly increases only slightly, the assembly N50 increases by a factor of almost 30 and the length of the longest sequence by a factor of 80, from 400 kbp to 32 Mbp (Figure 2; Additional file 1: Table S2). A plot of the final contig size shows that 78 contigs >10 Mb in length have been assembled from the short read shotgun data of the chimpanzee using *in silico* mate-pairs generated from Human chromosomes (Figure 2A). Correspondingly, the gene completeness as measured by BUSCO almost doubles, while the number of fragmented and missing BUSCO genes are reduced by factors of >2 and 4, respectively. The picture is qualitatively similar for the aye-aye assemblies, where the N50 is increased by more

than two times and the number of complete BUSCO genes doubles when using the human chromosome set as reference. Moreover, by using the gray mouse lemur as reference, the N50 of the aye-aye assembly increased by a factor of 20 and the number of complete BUSCO genes nearly triples (Figure 2B; Additional file 1: Table S2). Thus, our approach works even when using genomes as references that diverged more than 50 mya.

In order to time the generation of *in silico* mate-pair libraries, we have computed runtimes based on the human-chimp consensus genome. Runtime scales linearly with genome size and target coverage, but is largely independent of insert size (Additional file 1: Figure S1). On the customary laptop used for the benchmark, generating 10x mate-pairs takes about 6 seconds per 100Mbp.

To show that our method is flexible and can be applied across a broad taxonomic spectrum, we also generated experimental assemblies of the pork tapeworm (*Taenia solium*) and of yeast (*Saccharomyces cerevisiae*). In both cases, the assembly N50 showed substantial improvement, with an 80-fold and 11-fold increase for the pork tapeworm and yeast, respectively (Additional file 1: Table S3-4).

Discussion.

We present a simple, yet novel method for incorporating long-range distance information into *de novo* genome assembly from a reference genome through the generation of *in silico* mate-pair or scaffolding libraries. This is an essentially novel approach since other chromosome scaffolders, such as Chromosomer [17], MeDuSa [28], and AlignGraph [29], exploit distance information from a genome of closely a related organism to order and extend scaffold or contigs after the *de novo* assembly process, while *in silico* mate-pair libraries obtain distance information prior to the assembly process and can be adapted to any genome assembler that can take mate-pair sequences as input. Our results show that contiguity and completeness of genome assembly can be greatly improved through the use of *in silico* scaffolding libraries.

While *in silico* mate-pairs introduces minimal errors since position and arrangement is suggested and validated by shotgun data during the *de novo* assembly process, they cannot fully replace physical mate-pair and third generation (long reads) sequencing information, as it is probably an inadequate method for studying gene copy number variation, chromosomal structural variation and synteny.

An obvious drawback of this approach may be the introduction of assembly chimaeras; therefore, special consideration should be given to several factors prior to *in silico* mate-pair generation: (1) quality and quantity (coverage) of shotgun sequencing since the amount of initial data will affect the downstream assembly process. For our experimental assemblies, we have considered a minimum of 20-30x coverage of short insert (300-500 bp) paired-end shotgun libraries. Improvement and reduction of mis-assemblies can be expected if higher coverage and longer insert (> 500 bp) shotgun libraries are combined with *in silico* mate-pairs during the assembly. (2) The software chosen for mapping reads to the reference genome. Of the many short-read mappers available, we have used BWA [30]with default parameters as a proof of concept. It is likely that mis-assemblies can be further avoided by choosing different mappers with different parameters (e.g., AlignerBoost; [31]). (3) Like in any genome assembly, a fraction of mis-assemblies can be attributed to the assembly software used. While most genome assemblers produce useful assemblies, there is still a high degree of variability among the assemblies produced by the different genome assemblers [3], therefore choosing an adequate assembler for the amount, design and quality of data available is an important decision. (4) Finally the phylogenetic distance, quality and completeness of the reference genome, as well as its overall synteny and transposable element content will influence the final amount of mis-assemblies. We therefore recommend to use references as closely related as possible, and to hard mask repetitive regions in the references genomes prior to *in silico* mate-pair generation.

Despite the above-mentioned considerations, *in silico* mate-pair libraries offer several advantages over traditional mate-pair sequencing. First, extra-long-range scaffolding information can be easily obtained, since our tool has no maximum insert size and the upper limit of insert size remains to be explored in relation to syntenic conservation. Thus, it may also prove useful for super-scaffolding already existing scaffolded genome assemblies. Second, another advantage lies in the possibility to generate scaffolding libraries with precise and customized length, orientation, insert size and coverage from a mapped consensus genome. It is also possible to generate “repetitive element free” scaffolding libraries from hard-masked reference genomes, and reads from phylogenetically distant references may also be used to map onto conserved regions, such as exons. Additionally, because of the consensus calling from the mapped reads, allelic differences will be converted to ambiguous bases in the scaffolding libraries. Third, our method would also allow for consensus libraries to be generated if multiple species/individuals are mapped to the same reference prior to consensus calling of

mapped reads. Fourth, it is possible to use more than one reference genome for the generation *in silico* mate-pair libraries, while this still requires further development and experimentation, we have briefly explored this possibility and successfully assembled a tapeworm genome based on 4 reference genomes of closely related species (Additional file 1: Table S3).

Furthermore, adaptations of this rationale can be used to generate scaffolding libraries from uncorrected PacBio and Oxford nanopore reads if sufficient Illumina shotgun data is available.

Conclusions.

Overall, *in silico* generated mate-pairs represent a cost-effective strategy for incorporating chromosome-level and large scaffold distance information from related genomes directly into the *de novo* assembly process, requiring only standard Illumina shotgun sequencing data and a suitable reference genome. We have shown that it is even possible to use reference genomes that diverged more than 50 million years ago to improve genome quality measures and gene predictions. This is a novel and versatile solution to enrich and improve scaffolding in any genome assembler or chromosome scaffolder that can make use of mate-paired sequences. It is expected that *in silico* generated mate-pairs and scaffolding libraries will become a popular method in the genome assembly community, and that substantial improvement of the method will come about through its application.

METHODS

Sequences were downloaded from the NCBI SRA (*Daubentonia madagascariensis*: SRP007603; *Pan troglodytes*: SRP012268 [SRX142913]). Raw sequences were preprocessed with Prinseq [32] to remove forward/reverse duplicates and SeqPrep (https://github.com/jstjohn/SeqPrep) to remove adapters and merge overlapping reads. All preprocessed sequences were passed through *k*mer error correction using BFC [33] specifying the -s parameter for genome size. Multiplicity distribution of 23mers was carried out with Jellyfish2 [34] and KrATER (https://github.com/mahajrod/KrATER) in order to estimate coverage. *De novo* genome assembly was performed with SOAPdenovo2 [35], using the sparse_pregraph module with the following parameters: -g 15 -d 4 -e 4 -R -r 0, and parameter -M 1 during contig phase.

Multiple sets of *in silico* mate-pairs were generated with Cross-mates. First, paired-end reads of the target organism are mapped onto the reference genome with BWA and default settings [36]. Then, a consensus is computed using samtools/bcftools [37] with the samtools legacy variant calling model. Read pairs are sampled from the consensus in systematic mode, i.e. using exact insert sizes and sampling fragments at regularly spaced offsets, skipping regions of coverage lower than three. For the chimpanzee assembly, 14 scaffolding libraries ranging from 500bp to 200kb were generated from the human reference at a 10x coverage. For the aye-aye assembly, 16 scaffolding libraries ranging from 500bp to 20kb were generated from the human and lemur references, respectively, at a 10x coverage.

Finally, gaps in the assembly were filled-in using GapCloser (http://soap.genomics.org.cn). Assembly statistics were measured with Quast [38]. Completeness and biological accuracy of assembly contiguity was measured by searching for 3,023 vertebrate orthologs as implemented in BUSCO [39] on a set of protein predictions generated by Augustus 3.1.0 [40]. Reference assembly sequences used for generating scaffolding libraries and benchmarking were obtained from NCBI: human (GRCh38.p8; GCF_000001405); gray mouse lemur *Microcebus murinus* (Mmur_2.0; GCF_000165445); aye-aye (DauMad-1.0; GCA_000241425). All steps used for creating *in silico* scaffolding libraries, including Cross-mates, have been implemented in the pipeline Cross-Species Scaffolding, which is publicly available and maintained at Github (https://github.com/thackl/cross-species-scaffolding). An example of the Cross-mates command line scripts used for the pork tapeworm assembly experiments is included in Additional file 1 (Text S1).

For the pork tapeworm test assembly, *in silico* mate pairs were generated using the reference genomes of four species of tapeworms (*Taenia saginata, T. asiatica, T.multiceps* and *T. solium*) at a 10x coverage each, with multiple insert sizes ranging from 600 to 50,000 bp, and assembled in SOAPdenovo. For the yeast test, we used a different assembler (SPAdes; [41]) for *de novo* assembly with 10x coverage of 500, 2,000, 5,000 and 10,000 bp insert sizes *in silico* mate pairs.

Additional files

Additional file 1: Text S1, Tables S1 to S4, Figure S1.

AVAILABILITY OF SUPPORTING SOURCE CODE AND REQUIREMENTS

Project name: Cross-species scaffolding

Project home page: https://github.com/thackl/cross-species-scaffolding

Operating system(s): Unix

Programming language: Perl, Bash

Other requirements: Perl v5.10.1 or higher, Bash v4.2 or higher

License: MIT

DECLARATIONS

List of Abbreviations.

BUSCO (Benchmarking Universal Single-Copy Orthologs).

Ethics approval and consent to participate.

Not applicable.

Availability of data and material.

The datasets generated and/or analysed during the current study are available in the NCBI Short Read Archive repository: https://www.ncbi.nlm.nih.gov/sra/SRX142913 and https://www.ncbi.nlm.nih.gov/sra/SRP007603 for the chimpanzee and aye-aye, respectively.

Consent for publication.

Not applicable.

Competing interests.

The authors declare that they have no competing interests.

Funding.
This work was supported by European Research Council (consolidator grant 310763 GeneFlow to M.H.).

Authors' contributions.

JHG and TH conceived and designed the study, and developed the main pipeline of the method. KPK and MH made substantial intellectual contributions and actively participated in drafting, revising, and improving the manuscript and method. All authors read and approved the final manuscript.

REFERENCES

1. Ekblom R, Wolf JBW. A field guide to whole-genome sequencing, assembly and annotation. Evol. Appl. 2014;7:1026–42.

2. Damas J, O'Connor R, Farré M, Lenis VPE, Martell HJ, Mandawala A, et al. Upgrading short-read animal genome assemblies to chromosome level using comparative genomics and a universal probe set. Genome Res. 2017;27:875–84.

3. Bradnam KR, Fass JN, Alexandrov A, Baranay P, Bechner M, Birol I, et al. Assemblathon 2: evaluating de novo methods of genome assembly in three vertebrate species. Gigascience. 2013;2:10.

4. Baker M. De novo genome assembly: what every biologist should know. Nat. Methods. Nature Research; 2012;9:333–7.

5. Koepfli K-P, Paten B, Genome 10K Community of Scientists, O'Brien SJ. The Genome 10K Project: a way forward. Annu Rev Anim Biosci. 2015;3:57–111.

6. Lee H, Gurtowski J, Yoo S, Nattestad M, Marcus S, Goodwin S, et al. Third-generation sequencing and the future of genomics [Internet]. bioRxiv. 2016 [cited 2017 Jan 30]. p. 048603. Available from: http://biorxiv.org/content/early/2016/04/13/048603

7. Rhoads A, Au KF. PacBio Sequencing and Its Applications. Genomics Proteomics

Bioinformatics. 2015;13:278–89.

8. Mikheyev AS, Tin MMY. A first look at the Oxford Nanopore MinION sequencer. Mol. Ecol. Resour. 2014;14:1097–102.

9. Hackl T, Hedrich R, Schultz J, Förster F. proovread: large-scale high-accuracy PacBio correction through iterative short read consensus. Bioinformatics. 2014;30:3004–11.

10. Lin H-H, Liao Y-C. Evaluation and Validation of Assembling Corrected PacBio Long Reads for Microbial Genome Completion via Hybrid Approaches. PLoS One. journals.plos.org; 2015;10:e0144305.

11. Antipov D, Korobeynikov A, McLean JS, Pevzner PA. hybridSPAdes: an algorithm for hybrid assembly of short and long reads. Bioinformatics. 2016;32:1009–15.

12. Howe K, Wood JMD. Using optical mapping data for the improvement of vertebrate genome assemblies. Gigascience. 2015;4:10.

13. Vij S, Kuhl H, Kuznetsova IS, Komissarov A, Yurchenko AA, Van Heusden P, et al. Chromosomal-Level Assembly of the Asian Seabass Genome Using Long Sequence Reads and Multi-layered Scaffolding. PLoS Genet. 2016;12:e1005954.

14. Salzberg SL, Yorke JA. Beware of mis-assembled genomes. Bioinformatics. 2005;21:4320–1.

15. Elliott TA, Gregory TR. Do larger genomes contain more diverse transposable elements? BMC Evol. Biol. 2015;15:69.

16. Kim J, Larkin DM, Cai Q, Asan, Zhang Y, Ge R-L, et al. Reference-assisted chromosome assembly. Proc. Natl. Acad. Sci. U. S. A. 2013;110:1785–90.

17. Tamazian G, Dobrynin P, Krasheninnikova K, Komissarov A, Koepfli K-P, O’Brien SJ. Chromosomer: a reference-based genome arrangement tool for producing draft chromosome sequences. Gigascience. 2016;5:38.

18. Zhang SV, Zhuo L, Hahn MW. AGOUTI: improving genome assembly and annotation using transcriptome data. Gigascience. 2016;5:31.

19. Song L, Shankar DS, Florea L. Rascaf: Improving Genome Assembly with RNA Sequencing Data. Plant Genome [Internet]. 2016;9. Available from: http://dx.doi.org/10.3835/plantgenome2016.03.0027

20. Li YI, Copley RR. Scaffolding low quality genomes using orthologous protein sequences. Bioinformatics. Oxford Univ Press; 2013;29:160–5.

21. Zhu B-H, Song Y-N, Xue W, Xu G-C, Xiao J, Sun M-Y, et al. PEP_scaffolder: using (homologous) proteins to scaffold genomes. Bioinformatics. 2016;32:3193–5.

22. Salzberg SL, Phillippy AM, Zimin A, Puiu D, Magoc T, Koren S, et al. GAGE: A critical evaluation of genome assemblies and assembly algorithms. Genome Res. 2012;22:557–67.

23. Wetzel J, Kingsford C, Pop M. Assessing the benefits of using mate-pairs to resolve repeats in de novo short-read prokaryotic assemblies. BMC Bioinformatics. 2011;12:95.

24. van Heesch S, Kloosterman WP, Lansu N, Ruzius F-P, Levandowsky E, Lee CC, et al. Improving mammalian genome scaffolding using large insert mate-pair next-generation sequencing. BMC Genomics. 2013;14:257.

25. Lin H. Theoretical Bounds on Mate-Pair Information for Accurate Genome Assembly [Internet]. arXiv [q-bio.GN]. 2013. Available from: http://arxiv.org/abs/1310.1653

26. Finstermeier K, Zinner D, Brameier M, Meyer M, Kreuz E, Hofreiter M, et al. A Mitogenomic Phylogeny of Living Primates. PLoS One. Public Library of Science; 2013;8:e69504.

27. Perelman P, Johnson WE, Roos C, Seuánez HN, Horvath JE, Moreira MAM, et al. A Molecular Phylogeny of Living Primates. PLoS Genet. 2011;7:e1001342.

28. Bosi E, Donati B, Galardini M, Brunetti S, Sagot M-F, Lió P, et al. MeDuSa: a multi-draft based scaffolder. Bioinformatics. 2015;31:2443–51.

29. Bao E, Jiang T, Girke T. AlignGraph: algorithm for secondary de novo genome assembly guided by closely related references. Bioinformatics. 2014;30:i319–28.

30. Li H, Durbin R. Fast and accurate short read alignment with Burrows-Wheeler transform. Bioinformatics. 2009;25:1754–60.

31. Zheng Q, Grice EA. AlignerBoost: A Generalized Software Toolkit for Boosting Next-Gen Sequencing Mapping Accuracy Using a Bayesian-Based Mapping Quality Framework. PLoS Comput. Biol. 2016;12:e1005096.

32. Schmieder R, Edwards R. Quality control and preprocessing of metagenomic datasets. Bioinformatics. 2011;27:863–4.

33. Li H. BFC: correcting Illumina sequencing errors. Bioinformatics. 2015;31:2885–7.

34. Marçais G, Kingsford C. A fast, lock-free approach for efficient parallel counting of occurrences of k-mers. Bioinformatics. 2011;27:764–70.

35. Luo R, Liu B, Xie Y, Li Z, Huang W, Yuan J, et al. SOAPdenovo2: an empirically improved memory-efficient short-read de novo assembler. Gigascience. 2012;1:18.

36. Li H. Aligning sequence reads, clone sequences and assembly contigs with BWA-MEM. arXiv preprint arXiv. 2013;00:3.

37. Li H. A statistical framework for SNP calling, mutation discovery, association mapping and population genetical parameter estimation from sequencing data. Bioinformatics. 2011;27:2987–93.

38. Gurevich A, Saveliev V, Vyahhi N, Tesler G. QUAST: quality assessment tool for genome assemblies. Bioinformatics. Oxford Univ Press; 2013;29:1072–5.

39. Simão FA, Waterhouse RM, Ioannidis P, Kriventseva EV, Zdobnov EM. BUSCO: assessing genome assembly and annotation completeness with single-copy orthologs. Bioinformatics. Oxford Univ Press; 2015;31:3210–2.

40. Stanke M, Keller O, Gunduz I, Hayes A, Waack S, Morgenstern B. AUGUSTUS: ab initio prediction of alternative transcripts. Nucleic Acids Res. Oxford Univ Press; 2006;34:W435–9.

41. Bankevich A, Nurk S, Antipov D, Gurevich AA, Dvorkin M, Kulikov AS, et al. SPAdes: a new genome assembly algorithm and its applications to single-cell sequencing. J. Comput. Biol. 2012;19:455–77.

FIGURE CAPTIONS

Figure 1. Chart demonstrating the workflow implemented in Cross-Species Scaffolding for generating mate-pair libraries *in silico*. The approach is composed of three steps. In the first step, reads from shotgun libraries are mapped onto a set of repeat-masked reference chromosomes or genome assembly. In the second step, a large consensus fastq file is obtained from every chromosome or contig, generated only from the mapped reads. And finally, Cross-mates is used to simulate the sequencing of mate-pair or paired-end scaffolding libraries from the consensus fastq chromosomes.

Figure 2. A) Plot of final contig size for the chimpanzee and aye-aye genome assemblies. Chimpanzee genome assembled with shotgun only data (32x coverage) and with *in silico* mate-pairs generated from the human chromosomes using Cross-mates (see Materials and Methods). Aye-aye genome assembled with shotgun only data (22x coverage) and with *in silico* mate-pairs generated from the human chromosomes and the gray mouse lemur. B) Summary table of the assembly statistics showing chimpanzee and aye-aye results.

Figure 1

Click here to download Figure crossmates.svg.pdf

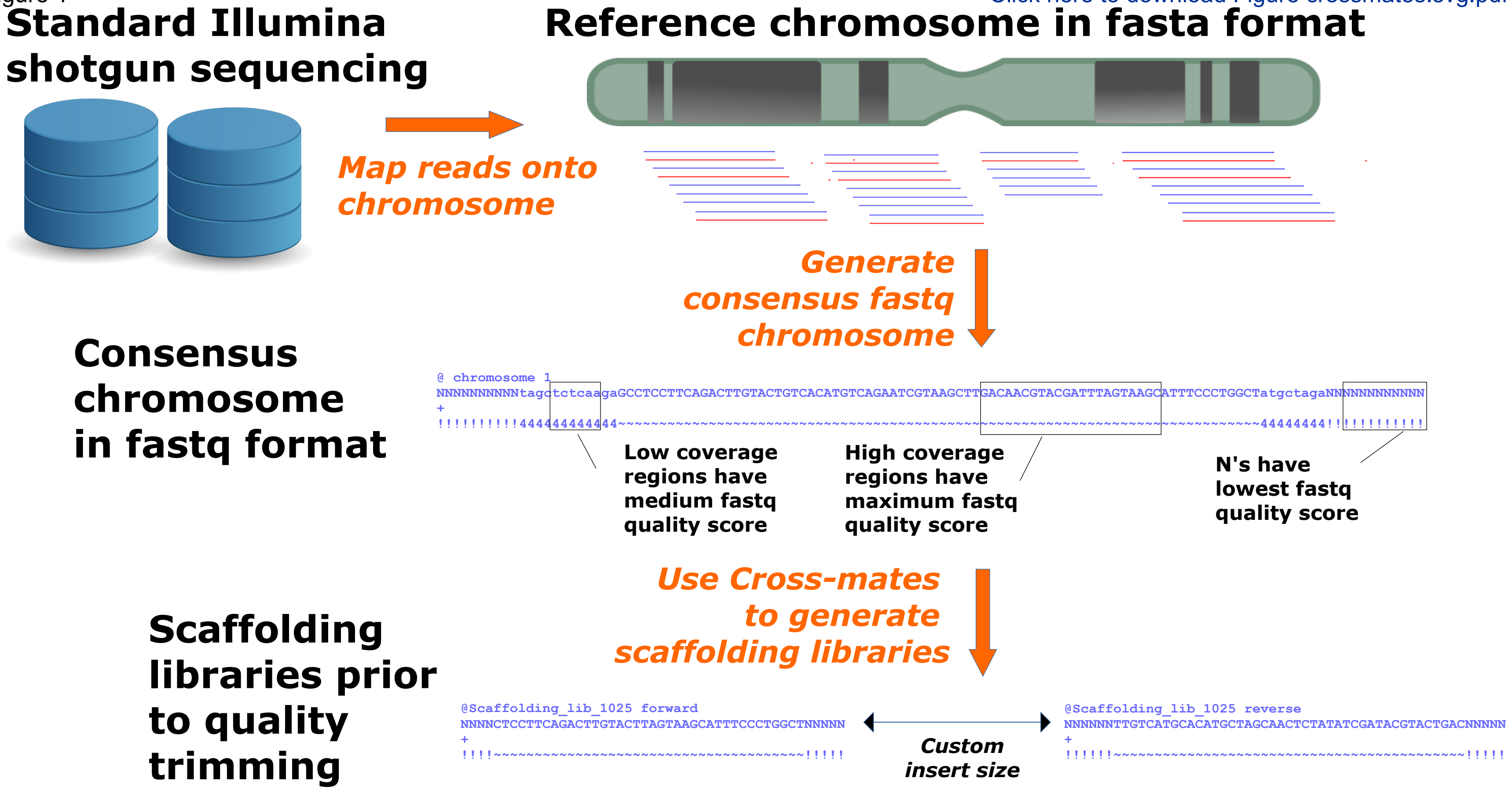

Figure 2

Click here to download Figure mates_drawing.svg.pdf

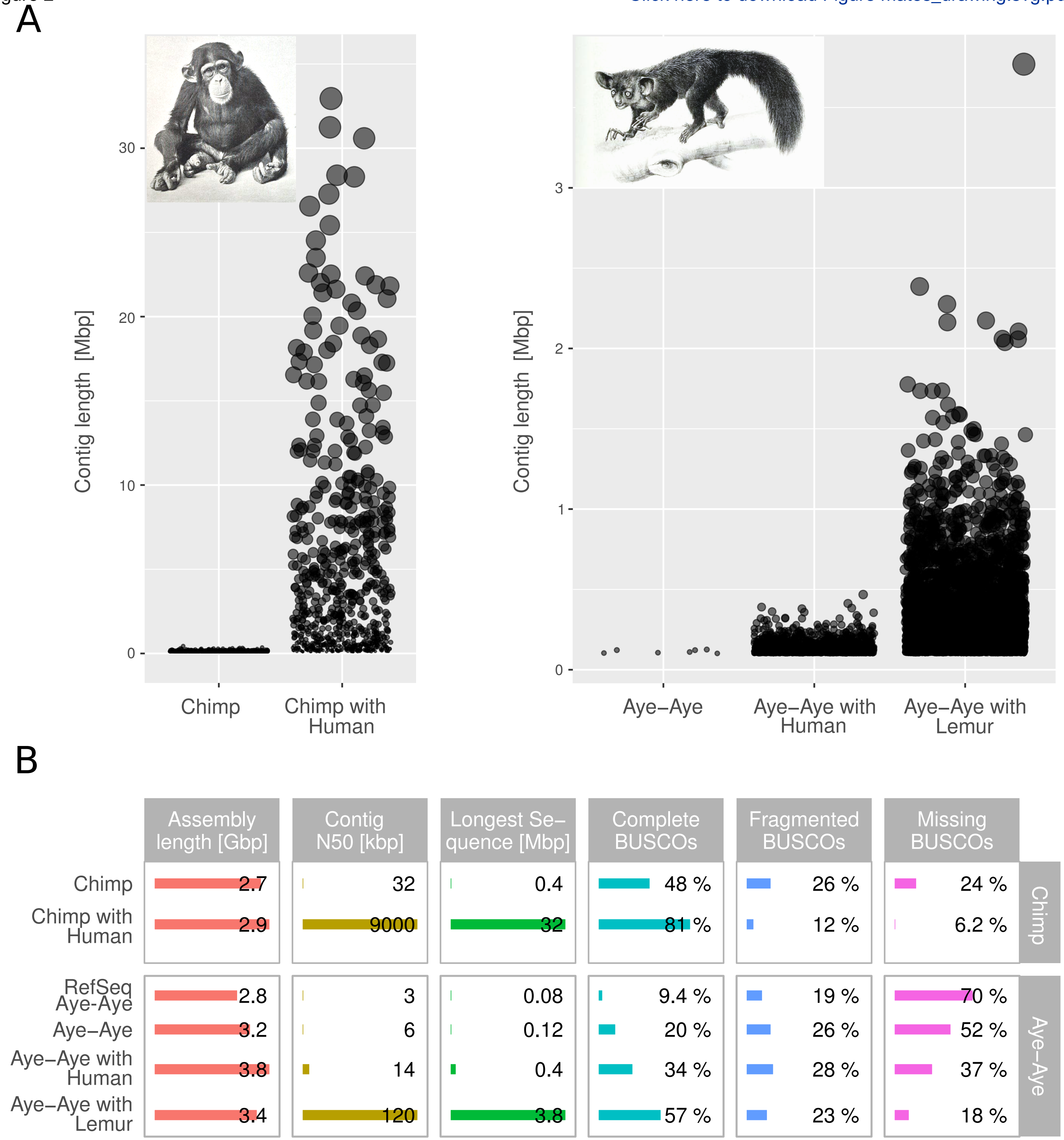

Click here to access/download
Supplementary Material
additional_files.pdf

 Click here to download Personal Cover Insili mate pair reviews for GS.pdf

Dear Editors,

Thank you very much for considering our manuscript. We found both reviews very helpful and constructive, and their suggestions have led to an improvement of our manuscript. We have addressed most of the issues pointed out by the reviewers, except for evaluation of assembly errors. While at first sight this seems to be a critical issue pointed out by both reviewers, we share the concerns of Reviewer #1 in that it is not crucial to evaluate assembly errors since our method only aids genome assembly and there are many pre- and post-assembly parameters that introduce assembly errors that are unrelated to our method. In addition, we have included a extended paragraph in the discussion of the manuscript pointing out multiple sources of assembly errors which should help readers to improve assembly with our method. Finally, we have included test assemblies from two additional organisms (tapeworm and yeast) and comparative tables (Additional file 1: Table S3-4).

Please find below our detailed answers to the individual points raised in the reviews.

Best Regards

Reviewer #1:
SUMMARY

The authors present a pipeline to generate in silico mate pair (MP) libraries of a query organism using a related genome sequence as reference. They show that their pipeline constructs libraries that, when used by the assembler SOAPdenovo, significantly improves various assembly metrics. The genome assembly approach used in this paper is to (1) generate a set of contigs using Illumina reads from a query genome, (2) Scaffold the contigs using their in silico generated MP libraries (3) fill the gaps with a gap closer. Several details on how the in silico MP libraries are generated are left out (see comments below). In general: Illumina reads from the query genome are mapped to a related reference genome. A artificial reference sequence in fastq format is constructed by consensus calling over each base pair based on the illumina query reads. The fastq quality values are determined by the coverage over each position. MP reads in fastq format are generated by simulating a position and an insert size on the artificial reference.

The pipeline for creating in silico mate pair libraries is easy to install and the code repository is well documented. The described pipeline seems to be novel and I find the assembly strategy appealing given that one is willing to accept the obvious risk of introducing errors from structural differences between query and reference. The authors show that not only the quantitative metrics improve with this method but also the quality in terms of recovered genes — which increases significantly. Also, the results show that scaffolding of the Aye-Aye genome is more conservative with a more distant reference (human) compared to a closer one (Lemur). These are both sound attributes of the method.

The authors do not directly evaluate assembly errors introduced by this approach — which is the obvious pitfall to point out. After some consideration, my opinion is that it's not crucial to evaluate assembly errors in this work. My reasons for this is that any evaluation of reasonable magnitude I can think of would not be directly capture flaws in the main idea, which is the construction of in silico MP reads. For instance, the pipeline is not tied to any specific assembler, thus assembly errors may vary with the assembly method using the in silico libraries. Also, it is relatively clear from context that this is an obvious drawback of such a method — although this should at least be explicitly mentioned in the manuscript. I would therefore leave any evaluation of assembly errors as discretionary. Below are some issues I believe need to be addressed.

We agree with the reviewer in that it is not crucial to evaluate assembly errors in our reconstructed genomes , since we are providing a novel method to assist assembly and not an assembly pipeline per se. We did, however, include a detail paragraph on mis-assemblies as suggested by the reviewer. Influential factors leading to mis-assemblies that we discuss are the following:

- *Quality and quantity (coverage) of shotgun sequencing.*
- *Mapping software and parameters.*
- *Assembly software and scaffolding algorithms.*
- *Phylogenetic distance and synteny of reference genome*
- *Quality and completeness of reference genome.*
- *Choice of length, coverage and insert-size distribution of in silico mate pairs.*

Furthermore, unlike CEGMA, the Augustus gene prediction pipeline heavily depends on genome contiguity and correct exon distance and order.

Therefore, the high BUSCO scores found in the Augustus predictions provide confidence that the genomes assembled with in silico mate pairs are biologically sound.

MINOR

* Several details of the pipeline needs to be described:

1. How are insert sizes and mate pair positions sampled from the reference? I assume at random by looking at seq-frag source, this needs to be described.

The cross-mates script is calling seq-frag in 'systematic' mode, i.e. each insert-size is used exactly as specified, and fragments are generated at regularly spaced offsets corresponding to the given coverage. We have added this information to the Methods section.

Our reasoning for going with the systematic mode is that even though seq-frag could be used to generate libraries with insert-sizes and fragment locations sampled from more realistic distributions, doing so would not provide any advantages for the downstream scaffolding process. However, it would unnecessarily increase computing time, and libraries would need to be generated at higher overall coverages to ensure sufficient coverage at every location along the assembly.

2. What happens if there are no reads in a region where a MP is sampled? will it be discarded, consists of only N's, or the sequence of the reference is taken? Please describe.

We are using the samtools/bcftools/vcfutils chain to generate the consensus with the samtools variant calling model and otherwise default settings. In this setup, the minimum depth for consensus calling is 3. Regions with lower coverage will be represented by N's and a quality of 0 in the consensus. Cross-mates calls seq-frag with default filtering, i.e. read pairs with a phred score below 20 will be dropped.

We've added a description of this default procedure to the manuscript (Methods section), and added a more detailed explanation on parameter adjustment and potential customization to the program documentation on github.

3. How are fastq qualities chosen in the reads? Specifically: "Additionally, because of the consensus calling from the mapped reads, allelic differences will be converted to ambiguous bases in the scaffolding libraries", what are the fastq qualities of those bases?

The consensus sequences are generated using samtools/bcftools with the samtools variant calling model. This is now explicitly stated in the Methods section. Details on how quality values are inferred and leveraged against read depth, variants, linkage, etc., are given in Li H., 2011, Bioinformatics, 10.1093/bioinformatics/btr509, and the documentations of the respective programs, e.g. http://www.htslib.org/doc/bcftools.html.

Generally speaking, with this approach also ambiguous bases do have good quality scores (similar to adjacent bases) provided the respective locations have sufficient support.

3. Which reads were used in the gap filling step, only illumina query or the in silico MP reads as well?.

Only illumina query were used. Ideally, in silico mate-pairs should only be used as a source of distance information within the assembly process, basically only providing distance and order information for the assembler to perform scaffolding or super-scaffolding of an existing scaffolded assembly.

4. What alignment parameters were used for BWA to align illumina to reference?

We have used default parameters for BWA mem for alignment, now mentioned in the manuscript, and in the improved documentation on github.

* Discussion: "This is an essentially novel approach since other chromosome scaffolders, such as Chromosomer [17] , MeDuSa [28] , and AlignGraph [29] , exploit distance information from genomes of closely related organisms to order and extend scaffold or contigs after the de novo assembly process." — Generating in silico MP libraries is on a high level the same as "exploit distance information", so this novelty motivation and and distinction to other methods needs to be refined.

The reviewer raises a valid point. The above mentioned scaffolders are limited to post-assembly scaffolding, and to a much closer phylogenetic distance. We have clarified this in the text.

* Provide runtime of the mate pair generation experiments.

We have computed runtimes for the generation of mate-pairs based on the human-chimp consensus genome presented in the manuscript. A plot and a description of the results have been added to the supplementary materials. Runtime scales linearly with genome size and target coverage, but is largely independent of insert size (Additional file 1: Figure S1). On the customary laptop used for the benchmark, generating 10X mate-pairs takes about 6 seconds per 100Mbp.

* Methods: "Assembly quality statistics were measured with Quast" A bit misleading if a reference is not used to calculate _quality_ of assembly such as misassembles. Use other term such as quantity metrics or listing the metrics explicitly.

The reviewer makes a valid point. We have used the term quality metric or metrics throughout the manuscript and refer to some of these Quast metrics directly.

* Discussion: "First, extra-long-range scaffolding information can be easily obtained, since our tool has no maximum insert size and the upper limit of insert size remains to be explored in relation to syntenic conservation" — Manuscript text says that mate pair libraries of any size can be generated but the pipeline script output options up to 200000. Fix discrepancy, e.g., by updating the code documentation that values are flexible and can easily be adjusted in the code. (Also, documentation says 200,000 kbp. —> 200kbp)

The insert sizes listed in the help screen for the cross-mates scripts are not the allowed choices, but rather the sizes used by default. The 200 kbp in the documentation also refers to this default. We have fixed the typo in the documentation, and also now explicitly state that the provided values are default values. Code-wise, there is no upper limit to the insert-size, although practical limitations may arise when using Mbp-sized inserts depending on the utilized hardware. Furthermore, insert sizes larger than reference contig sizes obviously will not result in any output.

* Explain "DauMad-1.0" in figure 2B.
We apologize for this oversight. We have corrected the mistake.

DISCRETIONARY

* How about comparing your assembly to the current chimp reference? For instance comparing the scaffolds (i.e., "Chimp with human") and the original contigs (i.e., "Chimp") using QUAST.

The reviewer puts forward a good suggestion, which we have included in the detailed table of the supplementary materials.

OTHER COMMENTS

The idea of using information of multiple reference sequences as done in MeDuSa or Multi-CAR might translate to a good extension for this approach as well, especially when multiple high quality references become available. Since coverage from each reference can be controlled in the in silico generation of mate pairs in cross-species-scaffolding pipeline, one possibility is to generate each mate pair library as a mixture of sampling from each artificial reference with, e.g., equal abundance.

The scaffolding tool (in this case SOAPdenovo) will in that case hopefully have more choices to select a correct join of contigs as it is more likely that at least one of the references are similar in any given region. This might conceptually remove some of the structural errors introduced by using a single reference sequence. The downside would be more "noisy" input to a typical genome scaffolder — with the "noise" being inconsistencies between read pairs within a library as they come from different references. This could potentially be handled with a scaffolder designed to take this specific input instead of a typical genome scaffolder.

Yes, we have explored using multiple genome references briefly and this is an interesting and fruitful avenue to explore. In the tapeworm supplementary assembly experiment, we show that it is possible to use up to four reference genomes.

Reviewer #2: The method implemented by the authors is well described and its impact may concern any person involved in the assembly process of a new genome project for which mate-pair library are not feasible or too expensive to produce.
However, the only way the authors validate their method is by using the N50 scaffolds and the gene completion information, which to me is not sufficient. To evaluate the quality of the final assembly proposed by their method, the author have to estimate the total number of misassemblies comparing to real mate-pair and using a genome reference. The different types of misassemblies have to be described, quantified and discussed. The gain in contiguity/gene completion obtained by their method has to be compared to the loss in accuracy created by chimeric scaffolds in order to really estimate the total gain of the method.
The dataset used for the method only corresponds to one mammal. Maybe, other genomes can be used to demonstrate the efficiency on a broad organisms spectrum.

We have included a paragraph in the discussion on the factors leading to mis-assemblies. In addition, we have tested our method on a broader set of organisms by generating assemblies of a tapeworm (Platyhelminthes) and of a yeast. The latter was assembled with the SPADES genome assembler. These results are included in Additional file 1: Table S3 and Table S4.
