# Supplementary material for: Improving draft genome contiguity with reference-derived in silico mate-pair libraries
Source: Gigascience. 2018 Apr 21;7(5):giy029. doi: 10.1093/gigascience/giy029 (PMC5967465; doi:10.1093/gigascience/giy029)
Supplement: GIGA-D-17-00092_Revision_2.pdf [file giy029_giga-d-17-00092_revision_2.pdf]

GigaScience

Improving draft genome contiguity with reference-derived in silico mate-pair libraries

--Manuscript Draft--

**Manuscript Number:**	GIGA-D-17-00092R2
**Full Title:**	Improving draft genome contiguity with reference-derived in silico mate-pair libraries
**Article Type:**	Technical Note
**Funding Information:**	European Research Council (310763) — Dr Michael Hofreiter
**Abstract:**	Background. Contiguous genome assemblies are a highly valued biological resource because of the higher number of completely annotated genes and genomic elements that are usable compared to fragmented draft genomes. Nonetheless, contiguity is difficult to obtain if only low coverage data and/or only distantly related reference genome assemblies are available.<br>Findings. In order to improve genome contiguity, we have developed Cross-Species Scaffolding - a new pipeline which imports long-range distance information directly into the de novo assembly process by constructing mate-pair libraries in silico.<br>Conclusions. We show how genome assembly metrics and gene prediction dramatically improve with our pipeline by assembling two primate genomes solely based on ~30x coverage of shotgun sequencing data.
**Corresponding Author:**	Jose Grau<br>Museum fur Naturkunde - Leibniz-Institut fur Evolutions- und Biodiversitatsforschung<br>Berlin, Berlin GERMANY
**Corresponding Author Secondary Information:**	
**Corresponding Author's Institution:**	Museum fur Naturkunde - Leibniz-Institut fur Evolutions- und Biodiversitatsforschung
**Corresponding Author's Secondary Institution:**	
**First Author:**	Jose Grau
**First Author Secondary Information:**	
**Order of Authors:**	Jose Grau
	Thomas Hackl
	Klaus-Peter Koepfli
	Michael Hofreiter
**Order of Authors Secondary Information:**	
**Response to Reviewers:**	Please find Respond to reviewers in the Personal letter. Thank you.
**Additional Information:**	
**Question**	**Response**
Are you submitting this manuscript to a special series or article collection?	No
**Experimental design and statistics**<br><br>Full details of the experimental design and statistical methods used should be given in the Methods section, as detailed in our Minimum Standards Reporting Checklist. Information essential to interpreting the data presented should be made available in the figure legends.	Yes

Powered by Editorial Manager® and ProduXion Manager® from Aries Systems Corporation

Have you included all the information requested in your manuscript?	
**Resources**<br><br>A description of all resources used, including antibodies, cell lines, animals and software tools, with enough information to allow them to be uniquely identified, should be included in the Methods section. Authors are strongly encouraged to cite Research Resource Identifiers (RRIDs) for antibodies, model organisms and tools, where possible.<br><br>Have you included the information requested as detailed in our Minimum Standards Reporting Checklist?	Yes
**Availability of data and materials**<br><br>All datasets and code on which the conclusions of the paper rely must be either included in your submission or deposited in publicly available repositories (where available and ethically appropriate), referencing such data using a unique identifier in the references and in the "Availability of Data and Materials" section of your manuscript.<br><br>Have you have met the above requirement as detailed in our Minimum Standards Reporting Checklist?	Yes

Manuscript

Click here to download Manuscript insilico mate pair manuscript draft for GS3.pdf

Improving draft genome contiguity with reference-derived *in silico* mate-pair libraries

José Horacio Grau [1†], Thomas Hackl [2†], Klaus-Peter Koepfli [3,4], Michael Hofreiter [5].

[1] Museum für Naturkunde Berlin, Leibniz-Institut für Evolutions- und Biodiversitätsforschung an der Humboldt-Universität zu Berlin. Invalidenstraße 43, 10115. Berlin, Germany.

[2] Massachusetts Institute of Technology, Department of Civil and Environmental Engineering, 15 Vassar Street, Cambridge, MA, 02139. USA.

[3] Smithsonian Conservation Biology Institute, National Zoological Park, 3001 Connecticut Avenue NW, Washington, D.C. 20008. USA.

[4] Theodosius Dobzhansky Center for Genome Bioinformatics, St. Petersburg State University, Sredniy Prospekt 41A, St. Petersburg, 199004. Russia.

[5] Faculty of Mathematics and Life Sciences, Institute of Biochemistry and Biology, Unit of General Zoology–Evolutionary Adaptive Genomics, University of Potsdam, Karl-Liebknecht-Straße 24-25, 14476 Potsdam, Germany.

[†] Authors contributed equally

Corresponding author:

José Horacio Grau

jose.grau@mfn-berlin.de

ABSTRACT

Background. Contiguous genome assemblies are a highly valued biological resource because of the higher number of completely annotated genes and genomic elements that are usable compared to fragmented draft genomes. Nonetheless, contiguity is difficult to obtain if only low coverage data and/or only distantly related reference genome assemblies are available.

Findings. In order to improve genome contiguity, we have developed Cross-Species Scaffolding - a new pipeline which imports long-range distance information directly into the *de novo* assembly process by constructing mate-pair libraries *in silico*.

Conclusions. We show how genome assembly metrics and gene prediction dramatically improve with our pipeline by assembling two primate genomes solely based on ~30x coverage of shotgun sequencing data.

KEYWORDS

Genome assembly, mate-pairs, in silico, scaffolding, shotgun sequencing

BACKGROUND

Accurate, complete and well-annotated genomes provide a wealth of information about the past, present and future of species and individuals, and therefore, constitute highly valuable resources for medical and biological research [1]. Thanks to the progress in DNA sequencing technology over the past decade, sequencing and assembly of a large variety of genomes from diverse branches of the tree of life has become possible, providing new insights into genomic architecture and phylogeny, as well as the functions of genes, RNAs, and other genomic features. Assemblies with at least near chromosome-level resolution are crucial for understanding genome biology due to the completeness of the information they contain, especially with regards to how loci are ordered and oriented along a chromosome [2]. Therefore, chromosome-level assemblies represent the aspired “gold standard”, but often this standard is hard to reach due to the difficulty of assembling the required long and continuous stretches of DNA [3]. While today more and more genomes are sequenced and assembled to chromosome level, assemblies of large genomes often remain highly fragmented [4]. Improvement of assembly contiguity is therefore a central issue in genome research: Improved contiguity increases the completeness of genes and genomic elements across the assembly, thereby facilitating better and more complete annotations and downstream analyses. Contiguity, thus, has been proposed as one of the key metrics for evaluating modern assemblies [5,6].

Despite recent advances in sequencing technologies and genome assembly approaches, obtaining a contiguous assembly of a large genome from short reads remains challenging. For this reason, sequencing technologies that are providing new means for contiguous assembly of large genomes are of great interest to the genomics community. Third generation long-read sequencing technologies such as PacBio [7] and Nanopore [8], either on their own or in combination with short-read data [9–11], as well as high quality long-insert clones and single-molecule restriction maps [12], are providing means by which more contiguous genome assemblies can be achieved [13]. However, the advantages of these approaches come at higher costs than simple short-read shotgun sequencing technologies.

Among the largest obstacles for assembling contiguous genomes, especially when using only short-reads, are low complexity regions and transposable elements [14]; in the case of some chordates and plants those regions may add up to over 50% of the total genome size [15]. Repetitive regions complicate and hinder contiguous *de novo* assemblies because the many highly similar copies scattered across the genome lead to a multitude of ambiguous, and often unresolvable paths in the underlying assembly graph. As a result, the obtained genome assemblies are fragmented, limiting their use for further analysis.

To increase contiguity, syntenic information may be imported from a closely related species for which a chromosome-level genome assembly is available [16]. While reference-assisted assemblies introduce occasional errors from genome rearrangements and gene duplications, this approach greatly reduces assembly fragmentation and allows better annotation and genomic feature analysis [16,17]. Although genome assemblies can be further optimized using additional transcriptome [18,19] or proteome data [20,21], contiguous assemblies are still difficult to obtain when it comes to large genomes, particularly if only low coverage sequencing data and/or only distantly related reference assemblies are available. Thus, poor contiguity in genome assemblies is a persistent limiting factor in the quest for high-quality genomic references and comprehensively annotated gene repertoires [22].

While paired-end sequencing is usually restricted to insert sizes below 500 bp and thus ineffective when it comes to resolving longer repeat regions, mate-pair sequencing can span across several kilobase pairs. Effective use of small, medium and large insert size mate-pair libraries has provided a dramatic improvement in assembly of large genomes [23,24]. Several *de novo* genome assemblers today can make use of the long-range information of mate-pairs, and the use of large insert size libraries (20-25 kb) can greatly increase contiguity. Altogether, a more contiguous assembly with larger scaffolds is easily obtained if provided with adequate and

sufficient mate-pair information [25]. Generation of mate-pair libraries and third-generation sequencing technologies, however, requires large amounts of high quality DNA, which can only be obtained from fresh and abundant samples. Furthermore, library preparation and sequencing are much more expensive than for short-read sequencing alone.

FINDINGS

To overcome the necessity for long-range sequencing data, which, depending on the project, is either expensive to generate or unobtainable in the first place, we developed a workflow to aid genome assembly, which only requires paired-end read data of the query organism, and which utilizes available reference genomes as a basis for generating long-range information by constructing mate-pair or scaffolding libraries *in silico* (Figure 1). This method has been implemented in a pipeline called Cross-Species Scaffolding.

To test the efficiency of *in silico* mate-pair libraries for assembling scaffolds, we assembled two genomes based only on standard Illumina shotgun sequencing. In the first assembly experiment, we assembled the chimpanzee genome by generating mate-pair libraries based on the human chromosome set. In the second experiment, we attempted to improve the genome of the aye-aye (*Daubentonia madagascariensis*), a basal nocturnal lemuroid primate with an estimated divergence time from humans between 70 and 80 million years [26,27], for which a very fragmented assembly was available. We generated mate-pair libraries using the human chromosome set as reference, and a second set using the gray mouse lemur (*Microcebus murinus*) genome, which diverged around 57-59 mya from the aye-aye [26,27]. As a quality metric in all assemblies, we have used the proportion of 3,023 vertebrate BUSCO (Benchmarking Universal Single-Copy Orthologs) genes that could be correctly and completely annotated. Assemblies were also assessed before and after the use of *in silico* mate-pairs for scaffold size (mean and maximum), number of scaffolds and scaffold size distribution. While the size of the chimpanzee assembly increases only slightly, the assembly N50 increases by a factor of almost 30 and the length of the longest sequence by a factor of 80, from 400 kbp to 32 Mbp (Figure 2; Additional file 1: Table S2). A plot of the final contig size shows that 78 contigs >10 Mb in length have been assembled from the short read shotgun data of the chimpanzee using *in silico* mate-pairs generated from Human chromosomes (Figure 2A). Correspondingly, the gene completeness as measured by BUSCO almost doubles, while the number of fragmented and missing BUSCO genes are reduced by factors of >2 and 4, respectively. The picture is qualitatively similar for the aye-aye assemblies, where the N50 is increased by more

than two times and the number of complete BUSCO genes doubles when using the human chromosome set as reference. Moreover, by using the gray mouse lemur as reference, the N50 of the aye-aye assembly increased by a factor of 20 and the number of complete BUSCO genes nearly triples (Figure 2B; Additional file 1: Table S2). Thus, our approach works even when using genomes as references that diverged more than 50 mya.

In order to time the generation of *in silico* mate-pair libraries, we have computed runtimes based on the human-chimp consensus genome. Runtime scales linearly with genome size and target coverage, but is largely independent of insert size (Additional file 1: Figure S1, Table S5). On the customary laptop used for the benchmark, generating 10x coverage of *in silico* mate-pairs takes about 6 seconds per 100Mbp.

To show that our method is flexible and can be applied across a broad taxonomic spectrum, we also generated experimental assemblies of the pork tapeworm (*Taenia solium*) and of yeast (*Saccharomyces cerevisiae*). In both cases, the assembly N50 showed substantial improvement, with an 80-fold and 11-fold increase for the pork tapeworm and yeast, respectively (Additional file 1: Table S3-4).

Discussion.

We present a simple, yet novel method for incorporating long-range distance information into *de novo* genome assembly from a reference genome through the generation of *in silico* mate-pair or scaffolding libraries. This is an essentially novel approach since other chromosome scaffolders, such as Chromosomer [17], MeDuSa [28], and AlignGraph [29], exploit distance information from a genome of closely a related organism to order and extend scaffold or contigs after the *de novo* assembly process, while *in silico* mate-pair libraries obtain distance information prior to the assembly process and can be adapted to any genome assembler that can take mate-pair sequences as input. Our results show that contiguity and completeness of genome assembly can be greatly improved through the use of *in silico* scaffolding libraries.

While the generation of *in silico* mate-pairs does not introduce errors such as paired-end contamination and chimeras, they cannot fully replace physical mate-pair and third generation (long reads) sequencing information, as it is probably an inadequate method for studying gene copy number variation, chromosomal structural variation and synteny.

A drawback of this approach may be the introduction of assembly chimaeras; therefore, special consideration should be given to several factors prior to *in silico* mate-pair generation:

(1) quality and quantity (coverage) of shotgun sequencing since the amount of initial data will affect the downstream assembly process. For our experimental assemblies, we have considered a minimum of 20-30x coverage of short insert (300-500 bp) paired-end shotgun libraries. Improvement and reduction of mis-assemblies can be expected if higher coverage and longer insert (> 500 bp) shotgun libraries are combined with *in silico* mate-pairs during the assembly. (2) The software chosen for mapping reads to the reference genome. Of the many short-read mappers available, we have used BWA [30] with default parameters as a proof of concept. It is likely that mis-assemblies can be further avoided by choosing different mappers with different parameters (e.g., AlignerBoost; [31]). (3) Like in any genome assembly, a fraction of mis-assemblies can be attributed to the assembly software used. While most genome assemblers produce useful assemblies, there is still a high degree of variability among the assemblies produced by the different genome assemblers [3]; therefore choosing an adequate assembler for the amount, design and quality of data available is an important decision. (4) Finally, the phylogenetic distance, quality and completeness of the reference genome, as well as its overall synteny and transposable element content will influence the final amount of mis-assemblies. We therefore recommend to use references as closely related as possible, and to hard mask repetitive regions in the references genomes prior to *in silico* mate-pair generation.

Despite the above-mentioned considerations, *in silico* mate-pair libraries offer several advantages over traditional mate-pair sequencing. First, extra-long-range scaffolding information can be easily obtained, since our tool has no maximum insert size and the upper limit of insert size remains to be explored in relation to syntenic conservation. Thus, it may also prove useful for super-scaffolding already existing scaffolded genome assemblies. Second, another advantage lies in the possibility to generate scaffolding libraries with precise and customized length, orientation, insert size and coverage from a mapped consensus genome. It is also possible to generate “repetitive element free” scaffolding libraries from hard-masked reference genomes, and reads from phylogenetically distant references may also be used to map onto conserved regions, such as exons. Additionally, because of the consensus calling from the mapped reads, allelic differences will be converted to ambiguous bases in the scaffolding libraries. Third, our method would also allow for consensus libraries to be generated if multiple species/individuals are mapped to the same reference prior to consensus calling of mapped reads. Fourth, it is possible to use more than one reference genome for the generation of *in silico* mate-pair libraries, while this still requires further development and experimentation,

we have briefly explored this possibility and successfully assembled a tapeworm genome based on 4 reference genomes of closely related species (Additional file 1: Table S3).

Furthermore, adaptations of this rationale can be used to generate scaffolding libraries from uncorrected PacBio and Oxford nanopore reads if sufficient Illumina shotgun data is available.

Conclusions.

Overall, *in silico* generated mate-pairs represent a cost-effective strategy for incorporating chromosome-level and large scaffold distance information from related genomes directly into the *de novo* assembly process, requiring only standard Illumina shotgun sequencing data and a suitable reference genome. We have shown that it is even possible to use reference genomes that diverged more than 50 million years ago to improve genome quality measures and gene predictions. This is a novel and versatile solution to enrich and improve scaffolding in any genome assembler or chromosome scaffolder that can make use of mate-paired sequences. It is expected that *in silico* generated mate-pairs and scaffolding libraries will become a popular method in the genome assembly community, and that substantial improvement of the method will come about through its application.

METHODS

Sequences were downloaded from the NCBI SRA (*Daubentonia madagascariensis*: SRP007603; *Pan troglodytes*: SRP012268 [SRX142913]). Raw sequences were preprocessed with Prinseq [32] to remove forward/reverse duplicates and SeqPrep (https://github.com/jstjohn/SeqPrep) to remove adapters and merge overlapping reads. All preprocessed sequences were passed through *k*mer error correction using BFC [33] specifying the -s parameter for genome size. Multiplicity distribution of 23mers was carried out with Jellyfish2 [34] and KrATER (https://github.com/mahajrod/KrATER) in order to estimate coverage. *De novo* genome assembly was performed with SOAPdenovo2 [35], using the sparse_pregraph module with the following parameters: -g 15 -d 4 -e 4 -R -r 0, and parameter -M 1 during contig phase.

Multiple sets of *in silico* mate-pairs were generated with Cross-mates. First, paired-end reads of the target organism are mapped onto the reference genome with BWA and default settings [36]. Then, a consensus is computed using samtools/bcftools [37] with the samtools legacy variant calling model. Read pairs are sampled from the consensus in systematic mode, i.e. using exact insert sizes and sampling fragments at regularly spaced offsets, skipping regions of coverage lower than three. For the chimpanzee assembly, 14 scaffolding libraries ranging from 500bp to 200kb were generated from the human reference at a 10x coverage. For the aye-aye assembly, 16 scaffolding libraries ranging from 500bp to 20kb were generated from the human and lemur references, respectively, at a 10x coverage.

Finally, gaps in the assembly were filled-in using GapCloser (http://soap.genomics.org.cn). Assembly statistics were measured with Quast [38]. Completeness and biological accuracy of assembly contiguity was measured by searching for 3,023 vertebrate orthologs as implemented in BUSCO [39] on a set of protein predictions generated by Augustus 3.1.0 [40]. Reference assembly sequences used for generating scaffolding libraries and benchmarking were obtained from NCBI: human (GRCh38.p8; GCF_000001405); gray mouse lemur *Microcebus murinus* (Mmur_2.0; GCF_000165445); aye-aye (DauMad-1.0; GCA_000241425). All steps used for creating *in silico* scaffolding libraries, including Cross-mates, have been implemented in the pipeline Cross-Species Scaffolding, which is publicly available and maintained at Github (https://github.com/thackl/cross-species-scaffolding). An example of the Cross-mates command line scripts used for the pork tapeworm assembly experiments is included in Additional file 1 (Text S1).

For the pork tapeworm test assembly, *in silico* mate pairs were generated using the reference genomes of four species of tapeworms (*Taenia saginata, T. asiatica, T.multiceps* and *T. solium*) at a 10x coverage each, with multiple insert sizes ranging from 600 to 50,000 bp, and assembled in SOAPdenovo. For the yeast test, we used a different assembler (SPAdes; [41]) for *de novo* assembly with 10x coverage of 500, 2,000, 5,000 and 10,000 bp insert sizes *in silico* mate pairs.

Additional files

Additional file 1: Text S1, Tables S1 to S4, Figure S1.

Additional file 2: QUAST pdf reports for yeast dataset.

Additional file 3: QUAST pdf reports for tapeworm dataset.

Additional file 4: QUAST pdf reports for chimp dataset.

AVAILABILITY OF SUPPORTING SOURCE CODE AND REQUIREMENTS

Project name: Cross-species scaffolding

Project home page: https://github.com/thackl/cross-species-scaffolding

Operating system(s): Unix

Programming language: Perl, Bash

Other requirements: Perl v5.10.1 or higher, Bash v4.2 or higher

License: MIT

DECLARATIONS

List of Abbreviations.

BUSCO (Benchmarking Universal Single-Copy Orthologs).

Ethics approval and consent to participate.

Not applicable.

Availability of data and material.

The datasets generated and/or analysed during the current study are available in the NCBI Short Read Archive repository: https://www.ncbi.nlm.nih.gov/sra/SRX142913 and https://www.ncbi.nlm.nih.gov/sra/SRP007603 for the chimpanzee and aye-aye, respectively.

Consent for publication.

Not applicable.

Competing interests.

The authors declare that they have no competing interests.

Funding.

This work was supported by European Research Council (consolidator grant 310763 GeneFlow to M.H.).

Authors' contributions.

JHG and TH conceived and designed the study, and developed the main pipeline of the method. KPK and MH made substantial intellectual contributions and actively participated in drafting, revising, and improving the manuscript and method. All authors read and approved the final manuscript.

REFERENCES

1. Ekblom R, Wolf JBW. A field guide to whole-genome sequencing, assembly and annotation. Evol. Appl. 2014;7:1026–42.

2. Damas J, O’Connor R, Farré M, Lenis VPE, Martell HJ, Mandawala A, et al. Upgrading short-read animal genome assemblies to chromosome level using comparative genomics and a universal probe set. Genome Res. 2017;27:875–84.

3. Bradnam KR, Fass JN, Alexandrov A, Baranay P, Bechner M, Birol I, et al. Assemblathon 2: evaluating de novo methods of genome assembly in three vertebrate species. Gigascience. 2013;2:10.

4. Baker M. De novo genome assembly: what every biologist should know. Nat. Methods. Nature Research; 2012;9:333–7.

5. Koepfli K-P, Paten B, Genome 10K Community of Scientists, O’Brien SJ. The Genome 10K

Project: a way forward. Annu Rev Anim Biosci. 2015;3:57–111.

6. Lee H, Gurtowski J, Yoo S, Nattestad M, Marcus S, Goodwin S, et al. Third-generation sequencing and the future of genomics [Internet]. bioRxiv. 2016 [cited 2017 Jan 30]. p. 048603. Available from: http://biorxiv.org/content/early/2016/04/13/048603

7. Rhoads A, Au KF. PacBio Sequencing and Its Applications. Genomics Proteomics Bioinformatics. 2015;13:278–89.

8. Mikheyev AS, Tin MMY. A first look at the Oxford Nanopore MinION sequencer. Mol. Ecol. Resour. 2014;14:1097–102.

9. Hackl T, Hedrich R, Schultz J, Förster F. proovread: large-scale high-accuracy PacBio correction through iterative short read consensus. Bioinformatics. 2014;30:3004–11.

10. Lin H-H, Liao Y-C. Evaluation and Validation of Assembling Corrected PacBio Long Reads for Microbial Genome Completion via Hybrid Approaches. PLoS One. journals.plos.org; 2015;10:e0144305.

11. Antipov D, Korobeynikov A, McLean JS, Pevzner PA. hybridSPAdes: an algorithm for hybrid assembly of short and long reads. Bioinformatics. 2016;32:1009–15.

12. Howe K, Wood JMD. Using optical mapping data for the improvement of vertebrate genome assemblies. Gigascience. 2015;4:10.

13. Vij S, Kuhl H, Kuznetsova IS, Komissarov A, Yurchenko AA, Van Heusden P, et al. Chromosomal-Level Assembly of the Asian Seabass Genome Using Long Sequence Reads and Multi-layered Scaffolding. PLoS Genet. 2016;12:e1005954.

14. Salzberg SL, Yorke JA. Beware of mis-assembled genomes. Bioinformatics. 2005;21:4320–1.

15. Elliott TA, Gregory TR. Do larger genomes contain more diverse transposable elements? BMC Evol. Biol. 2015;15:69.

16. Kim J, Larkin DM, Cai Q, Asan, Zhang Y, Ge R-L, et al. Reference-assisted chromosome assembly. Proc. Natl. Acad. Sci. U. S. A. 2013;110:1785–90.

17. Tamazian G, Dobrynin P, Krasheninnikova K, Komissarov A, Koepfli K-P, O'Brien SJ. Chromosomer: a reference-based genome arrangement tool for producing draft chromosome sequences. Gigascience. 2016;5:38.

18. Zhang SV, Zhuo L, Hahn MW. AGOUTI: improving genome assembly and annotation using transcriptome data. Gigascience. 2016;5:31.

19. Song L, Shankar DS, Florea L. Rascaf: Improving Genome Assembly with RNA Sequencing Data. Plant Genome [Internet]. 2016;9. Available from: http://dx.doi.org/10.3835/plantgenome2016.03.0027

20. Li YI, Copley RR. Scaffolding low quality genomes using orthologous protein sequences. Bioinformatics. Oxford Univ Press; 2013;29:160–5.

21. Zhu B-H, Song Y-N, Xue W, Xu G-C, Xiao J, Sun M-Y, et al. PEP_scaffolder: using (homologous) proteins to scaffold genomes. Bioinformatics. 2016;32:3193–5.

22. Salzberg SL, Phillippy AM, Zimin A, Puiu D, Magoc T, Koren S, et al. GAGE: A critical evaluation of genome assemblies and assembly algorithms. Genome Res. 2012;22:557–67.

23. Wetzel J, Kingsford C, Pop M. Assessing the benefits of using mate-pairs to resolve repeats in de novo short-read prokaryotic assemblies. BMC Bioinformatics. 2011;12:95.

24. van Heesch S, Kloosterman WP, Lansu N, Ruzius F-P, Levandowsky E, Lee CC, et al. Improving mammalian genome scaffolding using large insert mate-pair next-generation sequencing. BMC Genomics. 2013;14:257.

25. Lin H. Theoretical Bounds on Mate-Pair Information for Accurate Genome Assembly [Internet]. arXiv [q-bio.GN]. 2013. Available from: http://arxiv.org/abs/1310.1653

26. Finstermeier K, Zinner D, Brameier M, Meyer M, Kreuz E, Hofreiter M, et al. A Mitogenomic Phylogeny of Living Primates. PLoS One. Public Library of Science; 2013;8:e69504.

27. Perelman P, Johnson WE, Roos C, Seuánez HN, Horvath JE, Moreira MAM, et al. A Molecular Phylogeny of Living Primates. PLoS Genet. 2011;7:e1001342.

28. Bosi E, Donati B, Galardini M, Brunetti S, Sagot M-F, Lió P, et al. MeDuSa: a multi-draft

based scaffolder. Bioinformatics. 2015;31:2443–51.

29. Bao E, Jiang T, Girke T. AlignGraph: algorithm for secondary de novo genome assembly guided by closely related references. Bioinformatics. 2014;30:i319–28.

30. Li H, Durbin R. Fast and accurate short read alignment with Burrows-Wheeler transform. Bioinformatics. 2009;25:1754–60.

31. Zheng Q, Grice EA. AlignerBoost: A Generalized Software Toolkit for Boosting Next-Gen Sequencing Mapping Accuracy Using a Bayesian-Based Mapping Quality Framework. PLoS Comput. Biol. 2016;12:e1005096.

32. Schmieder R, Edwards R. Quality control and preprocessing of metagenomic datasets. Bioinformatics. 2011;27:863–4.

33. Li H. BFC: correcting Illumina sequencing errors. Bioinformatics. 2015;31:2885–7.

34. Marçais G, Kingsford C. A fast, lock-free approach for efficient parallel counting of occurrences of k-mers. Bioinformatics. 2011;27:764–70.

35. Luo R, Liu B, Xie Y, Li Z, Huang W, Yuan J, et al. SOAPdenovo2: an empirically improved memory-efficient short-read de novo assembler. Gigascience. 2012;1:18.

36. Li H. Aligning sequence reads, clone sequences and assembly contigs with BWA-MEM. arXiv preprint arXiv. 2013;00:3.

37. Li H. A statistical framework for SNP calling, mutation discovery, association mapping and population genetical parameter estimation from sequencing data. Bioinformatics. 2011;27:2987–93.

38. Gurevich A, Saveliev V, Vyahhi N, Tesler G. QUAST: quality assessment tool for genome assemblies. Bioinformatics. Oxford Univ Press; 2013;29:1072–5.

39. Simão FA, Waterhouse RM, Ioannidis P, Kriventseva EV, Zdobnov EM. BUSCO: assessing genome assembly and annotation completeness with single-copy orthologs. Bioinformatics. Oxford Univ Press; 2015;31:3210–2.

40. Stanke M, Keller O, Gunduz I, Hayes A, Waack S, Morgenstern B. AUGUSTUS: ab initio

prediction of alternative transcripts. Nucleic Acids Res. Oxford Univ Press; 2006;34:W435–9.

41. Bankevich A, Nurk S, Antipov D, Gurevich AA, Dvorkin M, Kulikov AS, et al. SPAdes: a new genome assembly algorithm and its applications to single-cell sequencing. J. Comput. Biol. 2012;19:455–77.

FIGURE CAPTIONS

Figure 1. Chart demonstrating the workflow implemented in Cross-Species Scaffolding for generating mate-pair libraries *in silico*. The approach is composed of three steps. In the first step, reads from shotgun libraries are mapped onto a set of repeat-masked reference chromosomes or genome assembly. In the second step, a large consensus fastq file is obtained from every chromosome or contig, generated only from the mapped reads. And finally, Cross-mates is used to simulate the sequencing of mate-pair or paired-end scaffolding libraries from the consensus fastq chromosomes.

Figure 2. A) Plot of final contig size for the chimpanzee and aye-aye genome assemblies. Chimpanzee genome assembled with shotgun only data (32x coverage) and with *in silico* mate-pairs generated from the human chromosomes using Cross-mates (see Materials and Methods). Aye-aye genome assembled with shotgun only data (22x coverage) and with *in silico* mate-pairs generated from the human chromosomes and the gray mouse lemur. B) Summary table of the assembly statistics showing chimpanzee and aye-aye results.

Figure 1

Click here to download Figure crossmates.svg.pdf

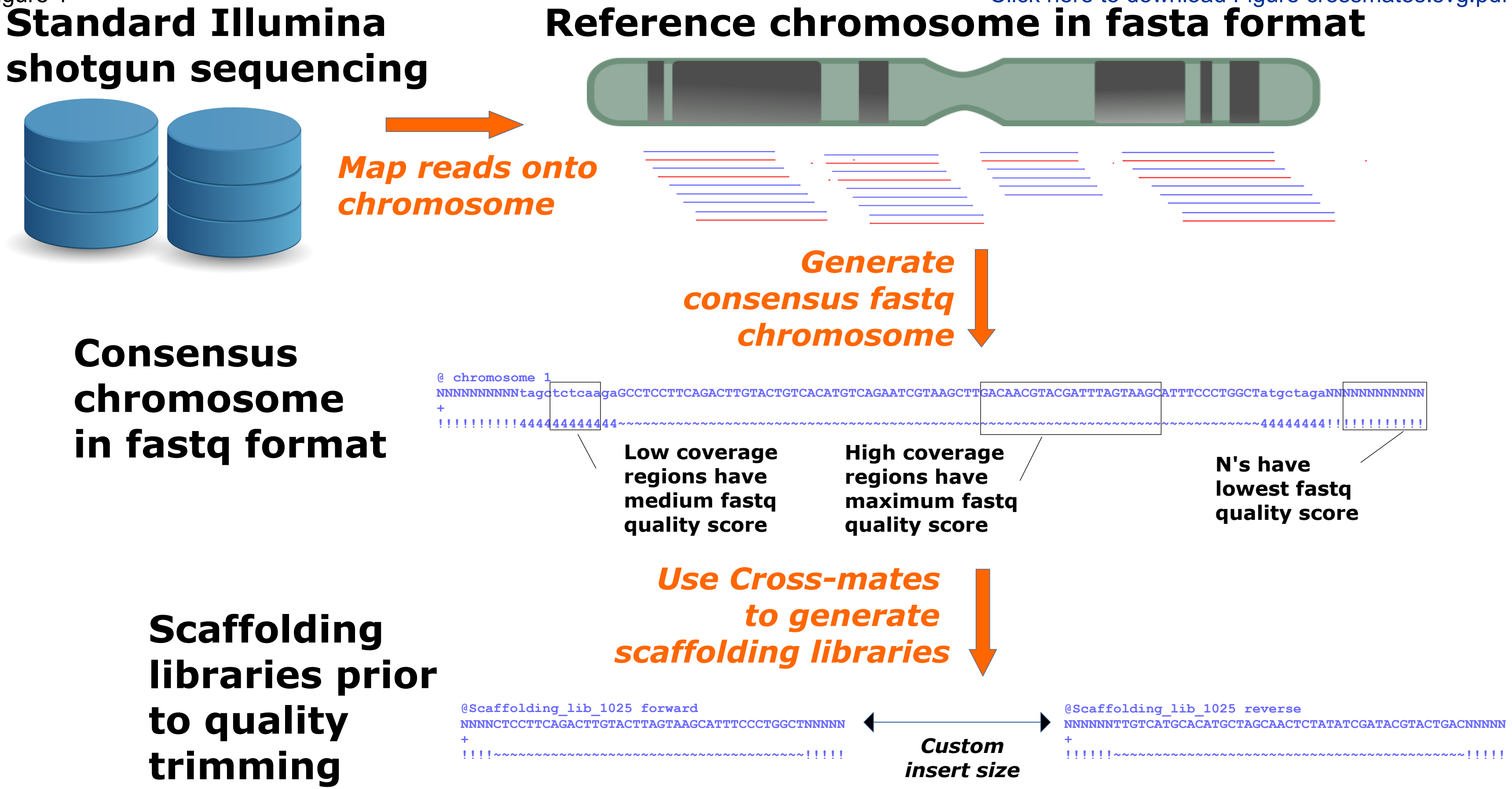

Figure 2

Click here to download Figure mates_drawing.svg.pdf

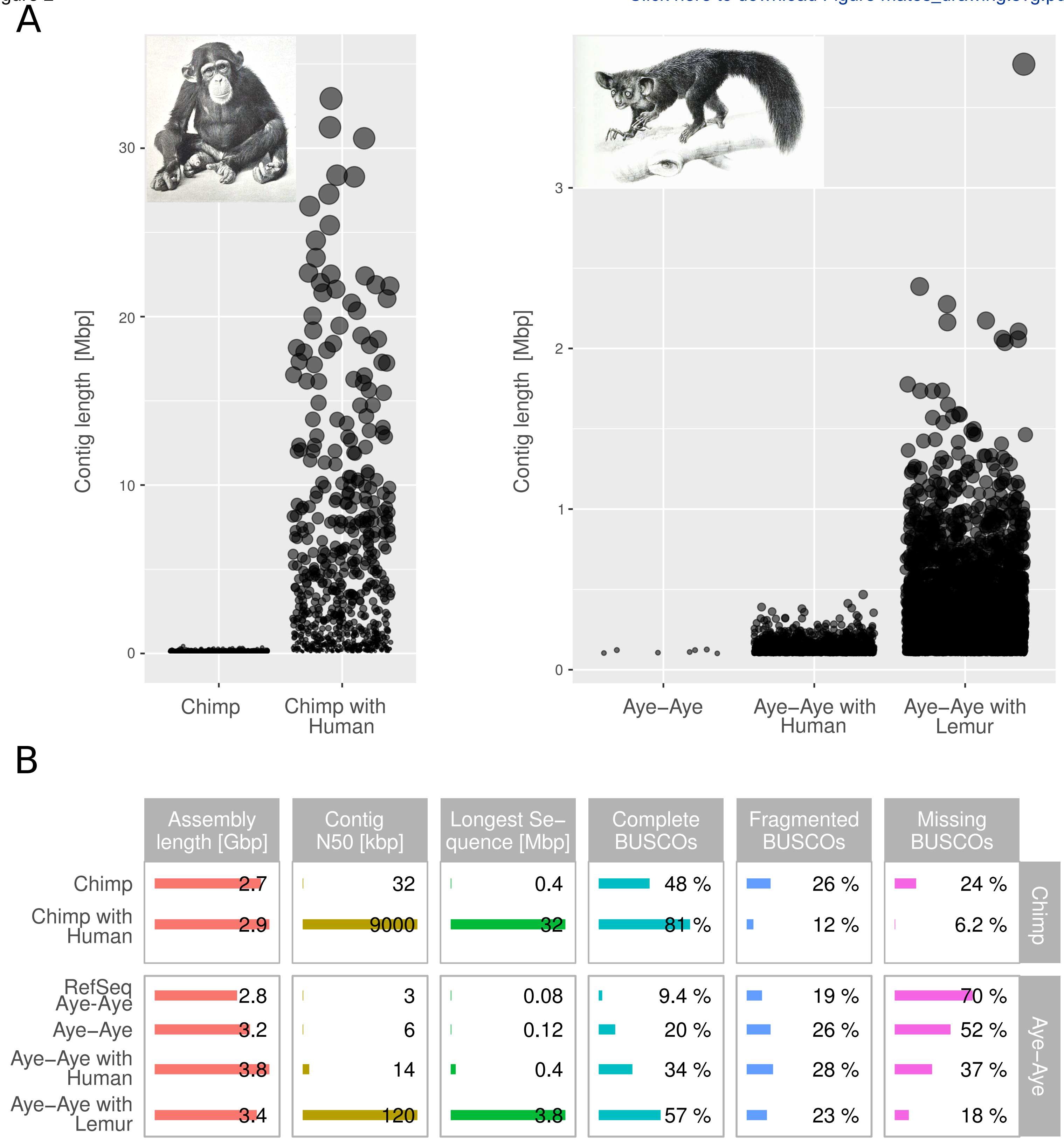

Additional file 1

Click here to access/download
Supplementary Material
Supplementary1.pdf

Additional file 2

Click here to access/download
Supplementary Material
yeast_report.pdf

Additional file 3

Click here to access/download
Supplementary Material
Taenia_report.pdf

Additional file 4

Click here to access/download
Supplementary Material
chimp_report.pdf

Click here to download Personal Cover Reviewer_reports_3.pdf

Dear Editors,

Thank you once again for considering our manuscript. Both reviews have been helpful and constructive and have led to a further improvement of our manuscript. We have addressed most of the issues pointed out by the reviewers. We have now included another additional file with the pdf reports of QUAST which also provide information on the mis-assembly evaluation. We hope to include this as merely discretionary as it has been impossible for us to pinpoint the origin (whether it was the assembly algorithm, quality of initial data, quality of reference assembly, and/or phylogenetic distance) of the mis-assemblies in our dataset. Nonetheless, it is clear that, despite mis-assemblies, the corrected N50 still increases considerably and that with pure shotgun sequences and a closely related reference genome, it is possible to generate a much more contiguous and biologically informative draft genome assembly.
Please find below our detailed answers to the individual points raised in the most recent reviews.

Reviewer reports:
Reviewer #1: The authors has addressed most of the points and I'm in general satisfied with the answers. I do have some significant remaining issues.

1. Runtime should be measured on the entire pipeline since a user have to run all three steps (alignment, pileup and seq-frag) to obtain the in silico mate pairs (my assumption at this point is especially that pileup is a costly way to iterate over alignments). I would advise the authors to provide the runtime of the three different steps separately (aligning, pileup, and seq-frag).

Indeed, pileup is the most time consuming step in the pipeline. We have added a runtime table to the supplementary materials for each of the steps separately.

2. Provide the mis-assembly metrics from QUAST. The authors only present the QUAST quantity metrics positively influenced by increased contiguity, but leaves the mis-assembly stats out. In my previous review I argued that I did not find an _evaluation_ of assembly errors and types necessary because this will depend on differences between reference and query as well as assembly algorithm. However, simply providing numbers such as adjusted N50 and the larger mis-assembly types for the

applicable datasets (where high quality reference exists, e.g., chimp, yeast) is useful and helps further development in this direction. In fact, the yeast data set will be very informative here as the reference and query are the same, thus removing the factor of structural differences between genomes.

We have attached the complete output of QUAST as pdf. mis-assemblies were calculated on alignments over 10 kbp against the reference assembly for all three of the presented datasets (Yeast, Taenia, and chimp). As expected, the cross-mates assemblies show a considerably larger amount of mis-assemblies, mainly translocations.

As the reviewer points out, the yeast dataset is the most interesting one since it compares a single-end assembly and an insilico mate-pair assembly, where the initial shotgun sequences and reference are the same organism. The few minor mis-assemblies that have been produced at the initial contig assembly stage can be attributed to data quality and coverage as well as assembly algorithm. A larger amount of mis-assemblies (50), almost all of which are translocations, have been introduced by our insilico mate-pair assembly. Interestingly, the translocations introduced in the initial contig assembly were not produced in the cross-mates assembly.

The Tapeworm (*Taenia solium*) dataset showed nearly 1000 mis-assemblies, with 600 relocations and 400 translocations. The larger amount and variety could be due to the fact that different species (*Taenia saginata*, *T. asiatica*, *T.multiceps* and *T. solium*) were used as references for the *in silico*-mate pair assembly.

Over 90% of the mis-assemblies in the Chimp *in silico* mate-pair assembly have been relocations. We can only assume that a large portion of mis-assemblies have been introduced because we used the human genome as reference.

In conclusion, mis-assemblies are hard to avoid, particularly when only shotgun sequences are used. Nonetheless, in all three examples presented in our manuscript, the NA50 is still considerably larger when insilico mate-pairs were used. Furthermore, by predicting genes through Augustus and then evaluating the predictions through BUSCO, we are confident in the biological accuracy of our gene calls. We, therefore, believe that the recovery of interpretable genomic information (i.e. gene calls) through in silico mate-pair aided assembly outweighs the introduction of mis-assemblies, especially in scenarios where physical mate-pair libraries are hard or impossible to obtain.

3. "While in silico mate-pairs introduces minimal errors since position and arrangement is suggested and validated by shotgun data during the de novo assembly process, they cannot fully replace …" Beginning of sentence a bit misleading. "introduces minimal errors" in this context can be interpreted as introduces errors in the assembly. MP has errors such as PE contamination and chimera, in silico generated MP on the other hand has "structural errors" corresponding to the differences between reference and query. I suggest something more precise in the style of "While in silico MP preparation does not introduce any [sequencing/library preparation] errors such as PE contamination, chimera,… they cannot replace"

We thank the reviewer for this suggestion. It now reads: "While the generation of in silico mate-pairs does not introduce errors such as paired-end contamination and chimeras, they cannot fully replace physical mate-pairs".

Discretionary
* "An obvious drawback of this approach may be the introduction of assembly chimaeras.." —> "A drawback of this approach may be the introduction of assembly chimaeras". Although I used "obvious" three times in my previous review, I don't think this belongs in a manuscript.

Again, we thank the reviewer for pointing this out. We have now removed the word "obvious" from the manuscript.

Reviewer #2: The authors have performed the recommended additional analyses needed to validate their method. The results they obtained showed that the assembly quality is comparable (or better) with simulated mate-pair reads comparing to real mate-pair reads and that is proved on several organisms. Thus, I am glad to accept the revised manuscript and recommend it for publication in GigaScience

Thank you.
