# Supplementary material for: Improving draft genome contiguity with reference-derived in silico mate-pair libraries
Source: Gigascience. 2018 Apr 21;7(5):giy029. doi: 10.1093/gigascience/giy029 (PMC5967465; doi:10.1093/gigascience/giy029)
Supplement: Additional Files [file giy029_supp.zip › chimp_report.pdf]

Report

	Pan.contigs	Pan.shotgun.scaffolds	Pan.crossmates.scaffolds
# contigs (>= 0 bp)	22311182	543105	387322
# contigs (>= 1000 bp)	690519	166796	22391
# contigs (>= 5000 bp)	47164	101771	2201
# contigs (>= 10000 bp)	3240	71696	1234
# contigs (>= 25000 bp)	4	32575	820
# contigs (>= 50000 bp)	0	11431	723
Total length (>= 0 bp)	3288617237	2726360032	2947169439
Total length (>= 1000 bp)	1651315972	2639881281	2870750141
Total length (>= 5000 bp)	322450145	2481733589	2834595366
Total length (>= 10000 bp)	39091263	2263681491	2828034793
Total length (>= 25000 bp)	110520	1632215540	2821935846
Total length (>= 50000 bp)	0	892232735	2818657214
# contigs	3240	71696	1234
Largest contig	29852	437303	32950256
Total length	39091263	2263681491	2828034793
Reference length	3231170666	3231170666	3231170666
GC (%)	38.51	40.16	40.65
Reference GC (%)	40.79	40.79	40.79
N50	11706	40289	9467859
NG50	-	25464	8141167
N75	10685	23283	5118318
NG75	-	-	3299374
L50	1417	16773	87
LG50	-	31916	110
L75	2293	35291	187
LG75	-	-	260
# misassemblies	0	1069	13722
# misassembled contigs	0	1051	411
Misassembled contigs length	0	79569937	2569807802
# local misassemblies	0	16126	51119
# unaligned mis. contigs	0	169	207
# unaligned contigs	6 + 3 part	13990 + 35874 part	432 + 754 part
Unaligned length	93389	520478695	875787196
Genome fraction (%)	1.207	53.996	60.447
Duplication ratio	1.000	0.999	1.000
# N's per 100 kbp	0.00	385.74	6014.94
# mismatches per 100 kbp	22.10	41.16	50.61
# indels per 100 kbp	3.25	26.18	30.17
Largest alignment	29852	436798	1328590
Total aligned length	38997297	1741718111	1952326244
NA50	11698	27622	121386
NGA50	-	12644	79252
NA75	10676	10919	-
LA50	1419	21710	5222
LGA50	-	47762	7253
LA75	2296	54756	-

All statistics are based on contigs of size >= 10000 bp, unless otherwise noted (e.g., "# contigs (>= 0 bp)" and "Total length (>= 0 bp)" include all contigs).

Misassemblies report

	Pan.contigs	Pan.shotgun.scaffolds	Pan.crossmates.scaffolds
# misassemblies	0	1069	13722
# relocations	0	1024	13450
# translocations	0	44	263
# inversions	0	1	9
# misassembled contigs	0	1051	411
Misassembled contigs length	0	79569937	2569807802
# local misassemblies	0	16126	51119
# misassemblies caused by fragmented reference	0	0	0
# unaligned mis. contigs	0	169	207
# mismatches	8619	718100	988511
# indels	1267	456703	589345
# indels (<= 5 bp)	1244	294417	403731
# indels (> 5 bp)	23	162286	185614
Indels length	1833	6291341	6645811

All statistics are based on contigs of size >= 10000 bp, unless otherwise noted (e.g., "# contigs (>= 0 bp)" and "Total length (>= 0 bp)" include all contigs).

Unaligned report

	Pan.contigs	Pan.shotgun.scaffolds	Pan.crossmates.scaffolds
# fully unaligned contigs	6	13990	432
Fully unaligned length	91430	210481865	13666789
# partially unaligned contigs	3	35874	754
Partially unaligned length	1959	309996830	862120407
# N's	0	8731822	170104500

All statistics are based on contigs of size >= 10000 bp, unless otherwise noted (e.g., "# contigs (>= 0 bp)" and "Total length (>= 0 bp)" include all contigs).

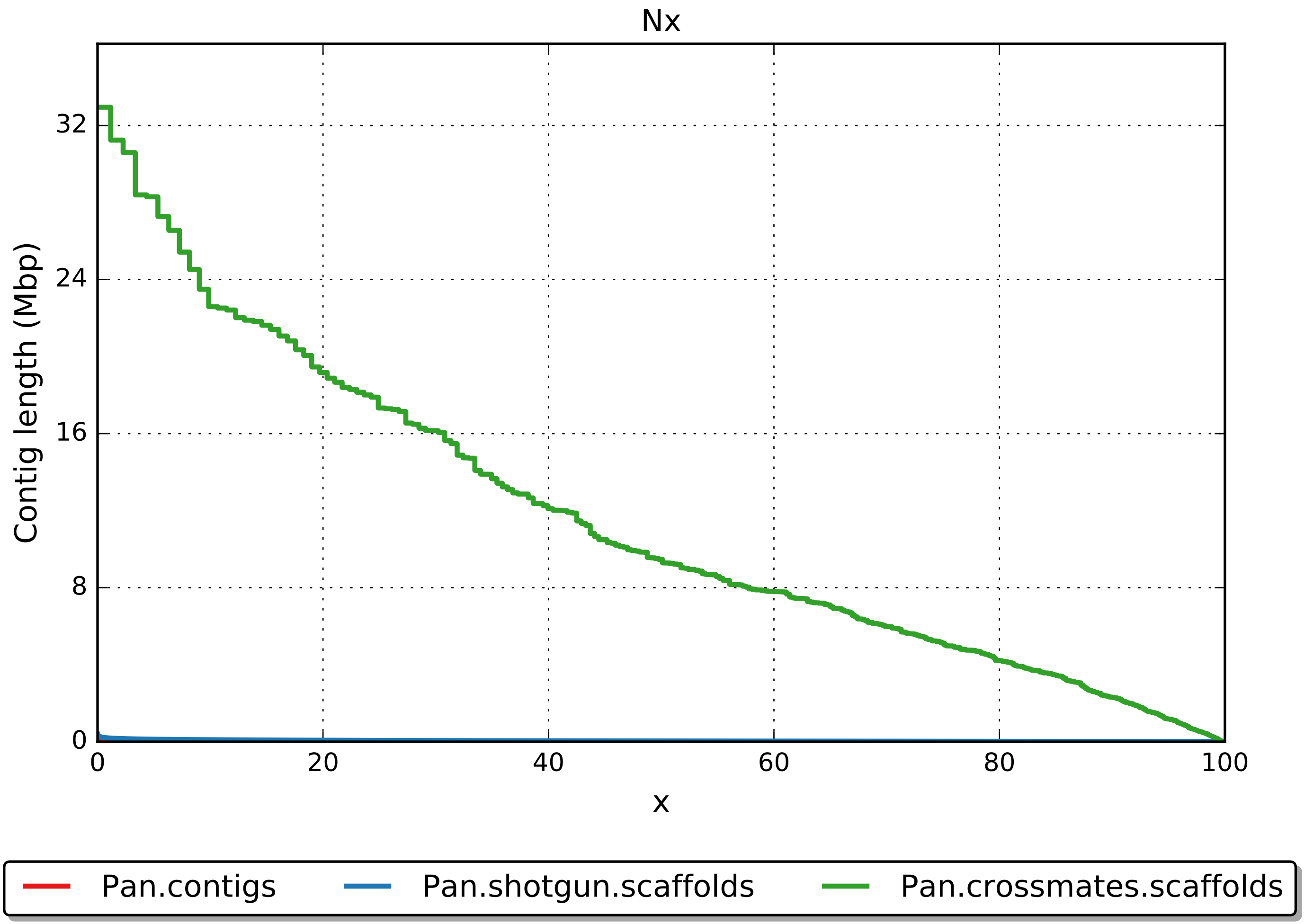

Nx
Contig length (Mbp)
0
8
16
24
32
0
20
40
60
80
100
x
Pan.contigs
Pan.shotgun.scaffolds
Pan.crossmates.scaffolds

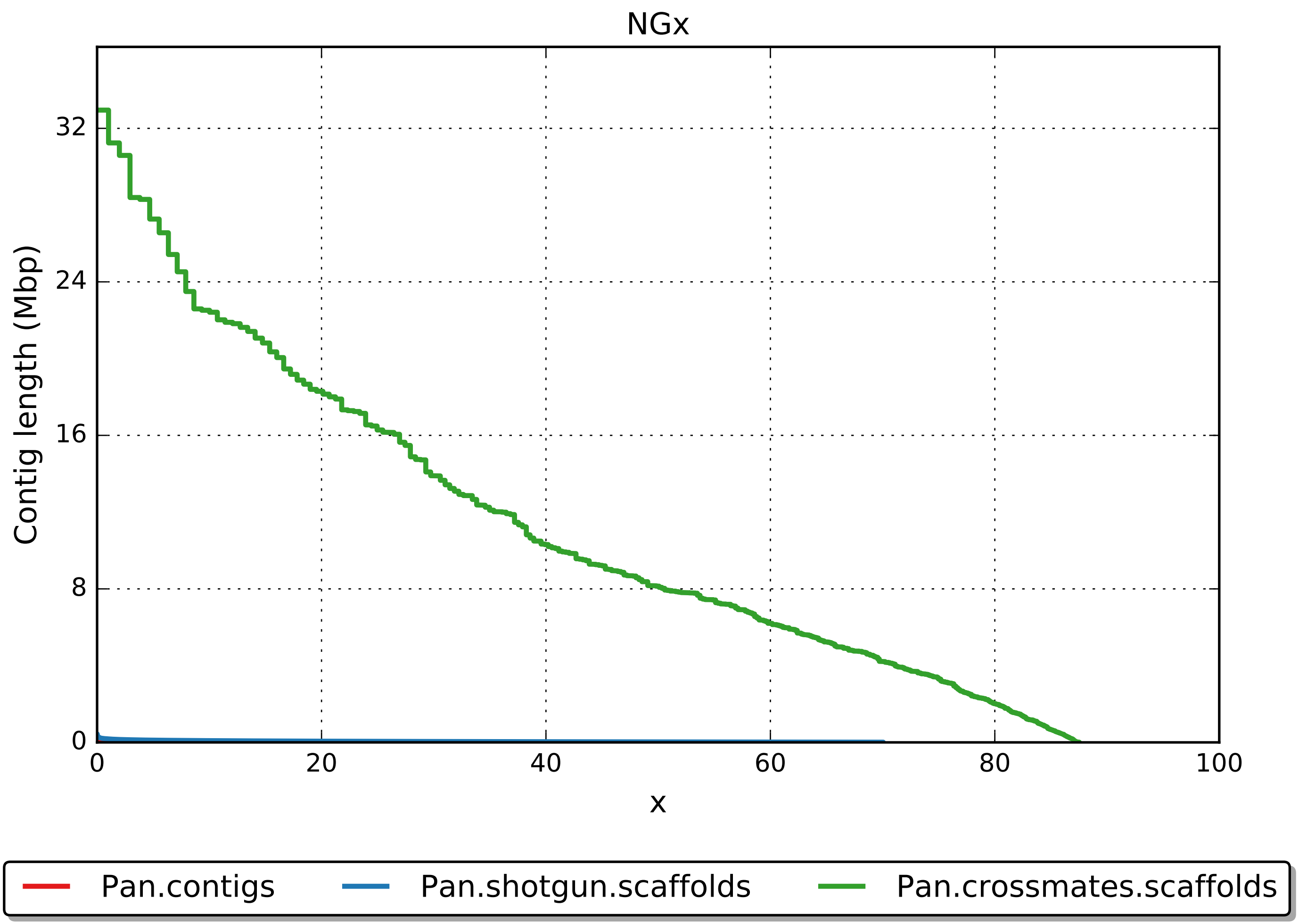
NGx
Contig length (Mbp)
32
24
16
8
0
0
20
40
60
80
100
x
Pan.contigs
Pan.shotgun.scaffolds
Pan.crossmates.scaffolds

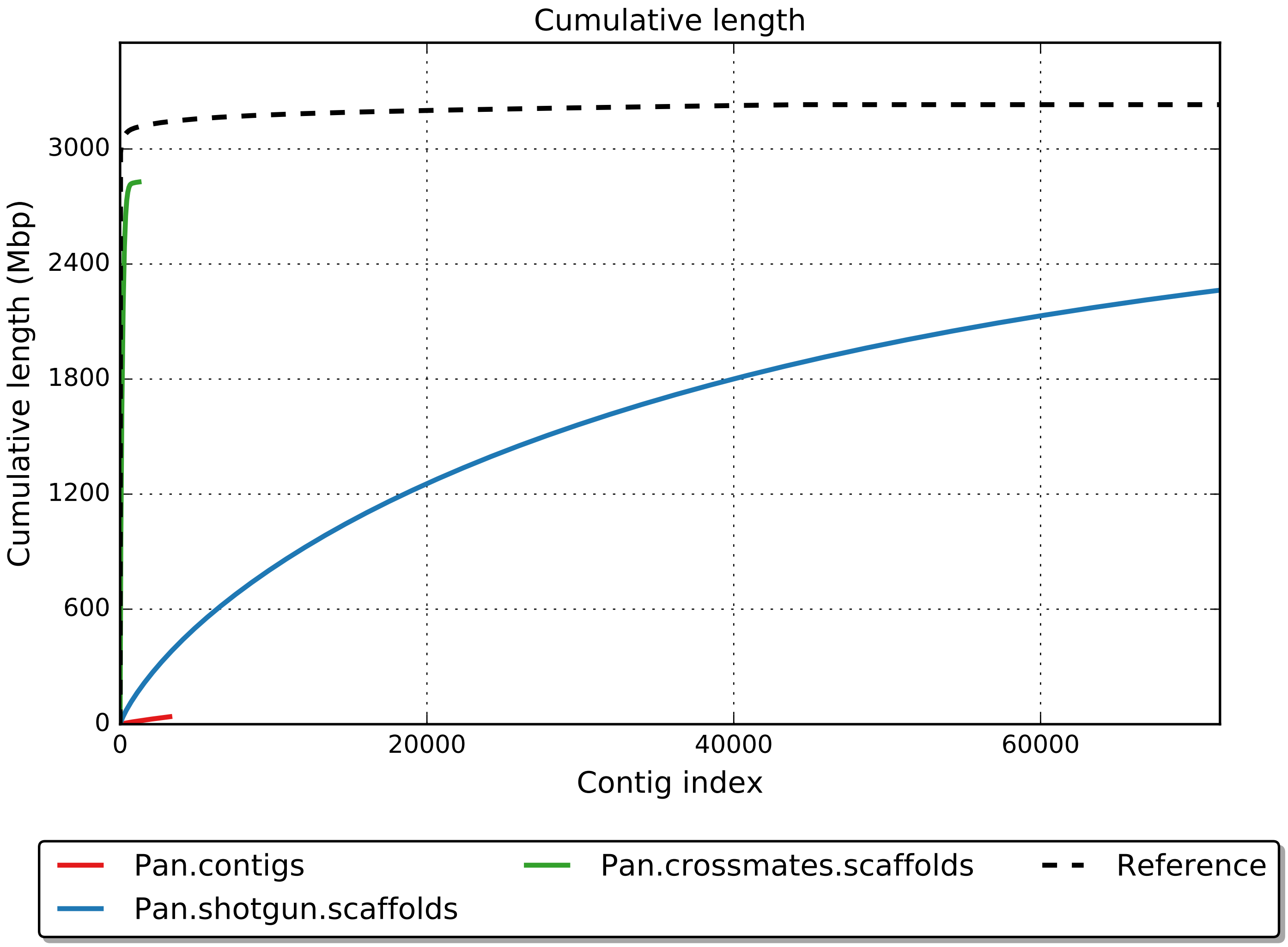
Cumulative length
Cumulative length (Mbp)
0
600
1200
1800
2400
3000
0
20000
40000
60000
Contig index
Pan.contigs
Pan.crossmates.scaffolds
Reference
Pan.shotgun.scaffolds

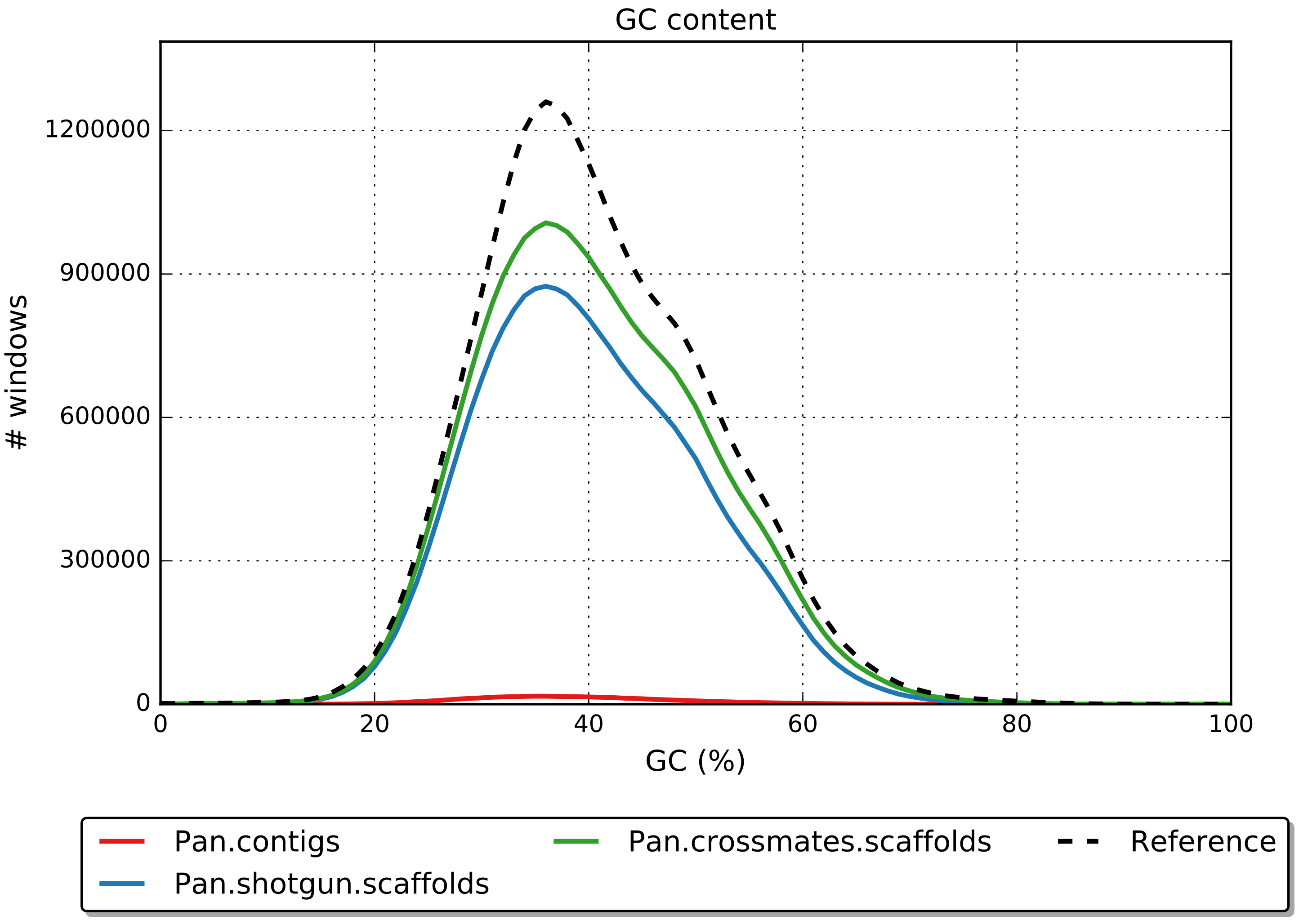
GC content
windows
1200000
900000
600000
300000
0
0
20
40
60
80
100
GC (%)
Pan.contigs
Pan.shotgun.scaffolds
Pan.crossmates.scaffolds
Reference

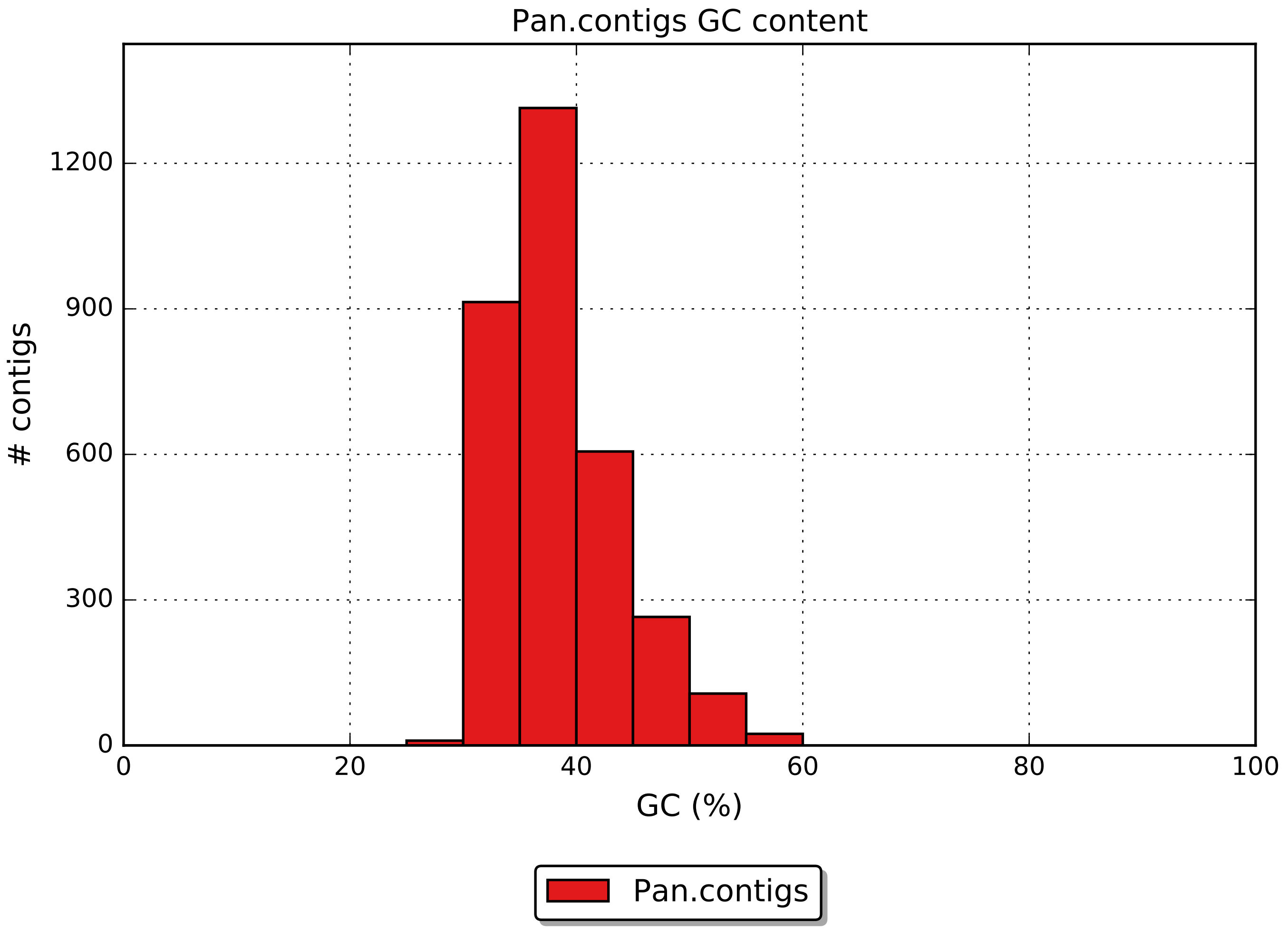

Pan.contigs GC content
contigs
1200
900
600
300
0
0
20
40
60
80
100
GC (%)
Pan.contigs

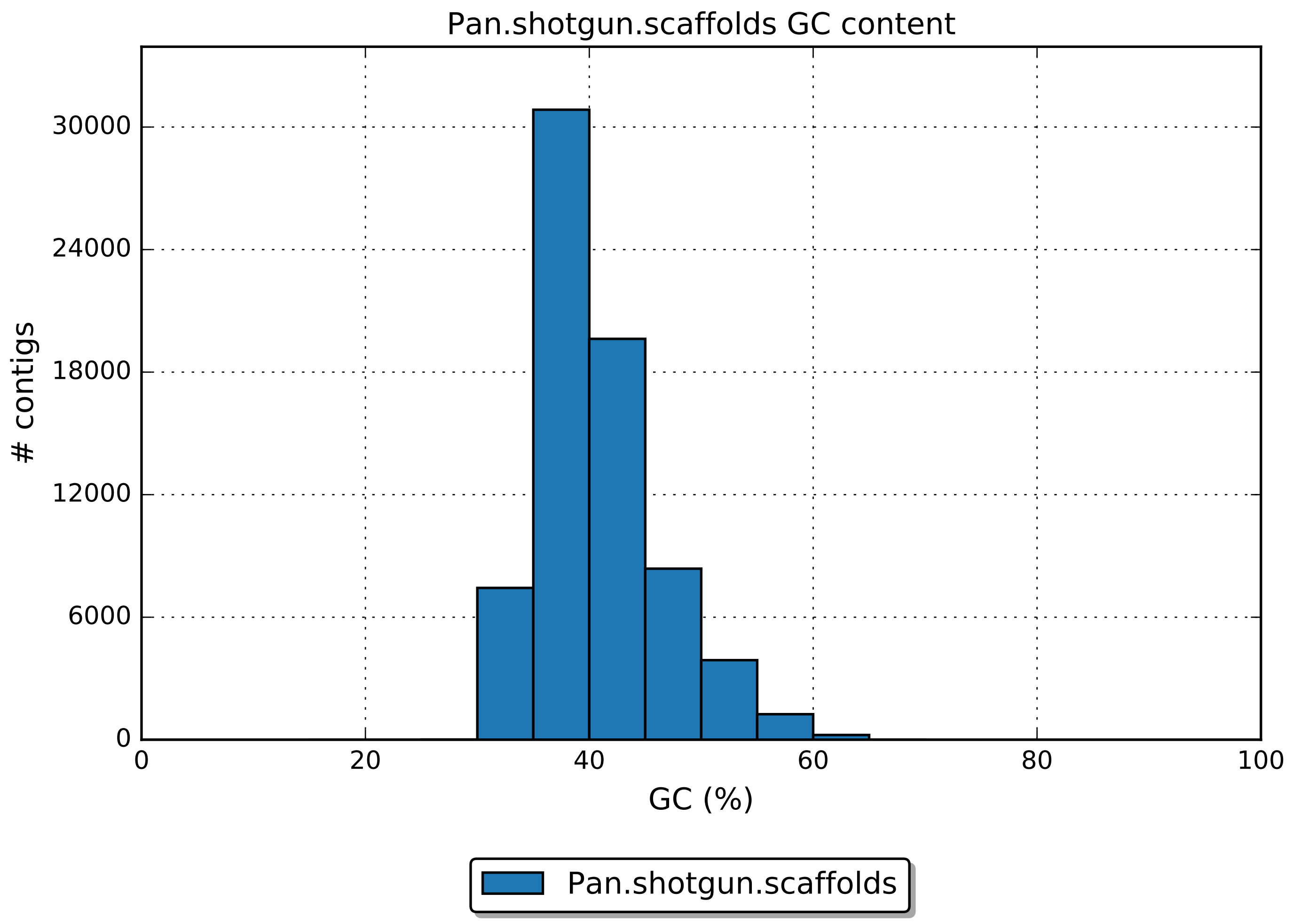

Pan.shotgun.scaffolds GC content
contigs
30000
24000
18000
12000
6000
0
0
20
40
60
80
100
GC (%)
Pan.shotgun.scaffolds

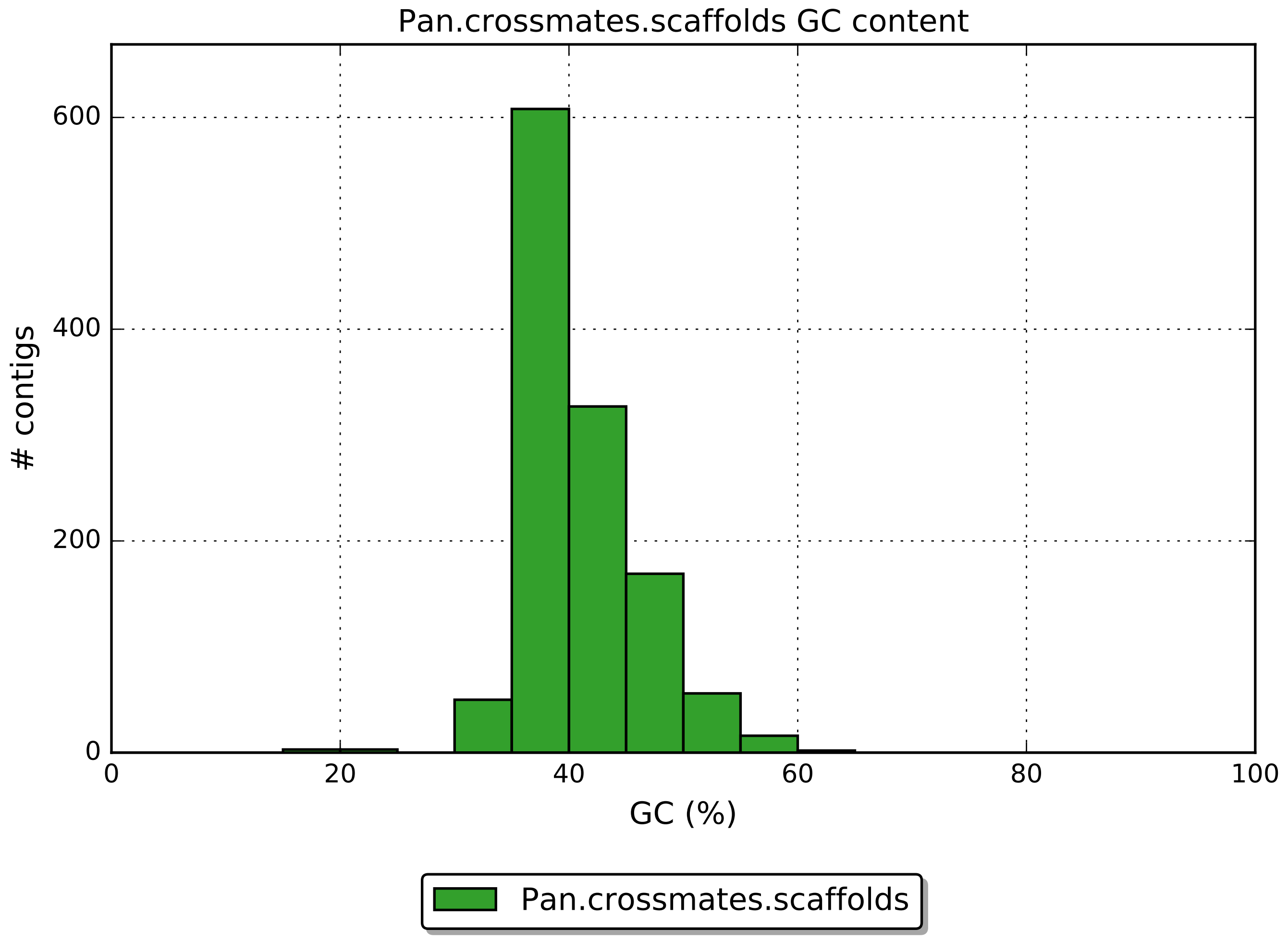
Pan.crossmates.scaffolds GC content
600
400
200
0
contigs
0
20
40
60
80
100
GC (%)
Pan.crossmates.scaffolds

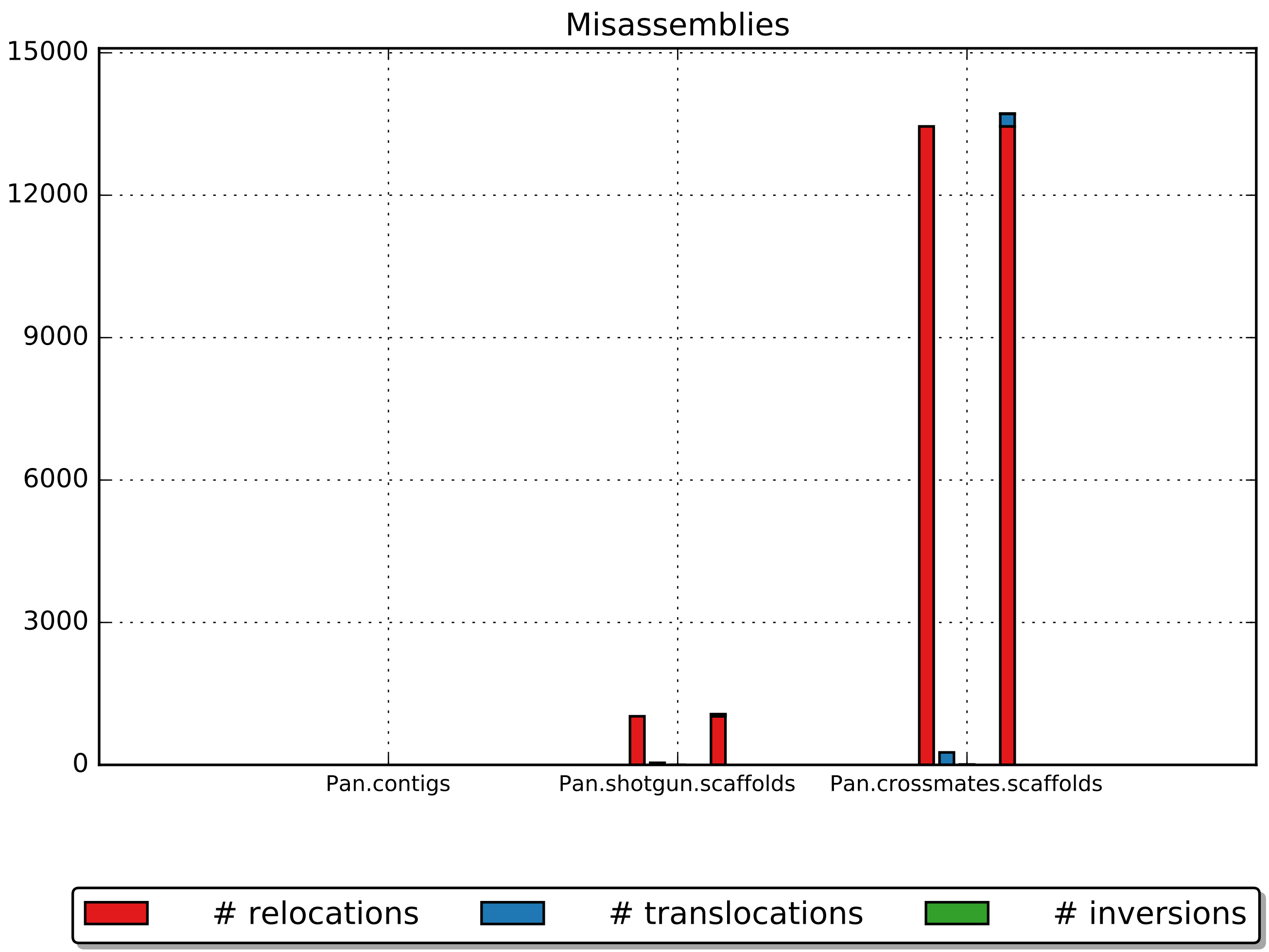

Misassemblies
15000
12000
9000
6000
3000
0
Pan.contigs
Pan.shotgun.scaffolds
Pan.crossmates.scaffolds
relocations
translocations
inversions

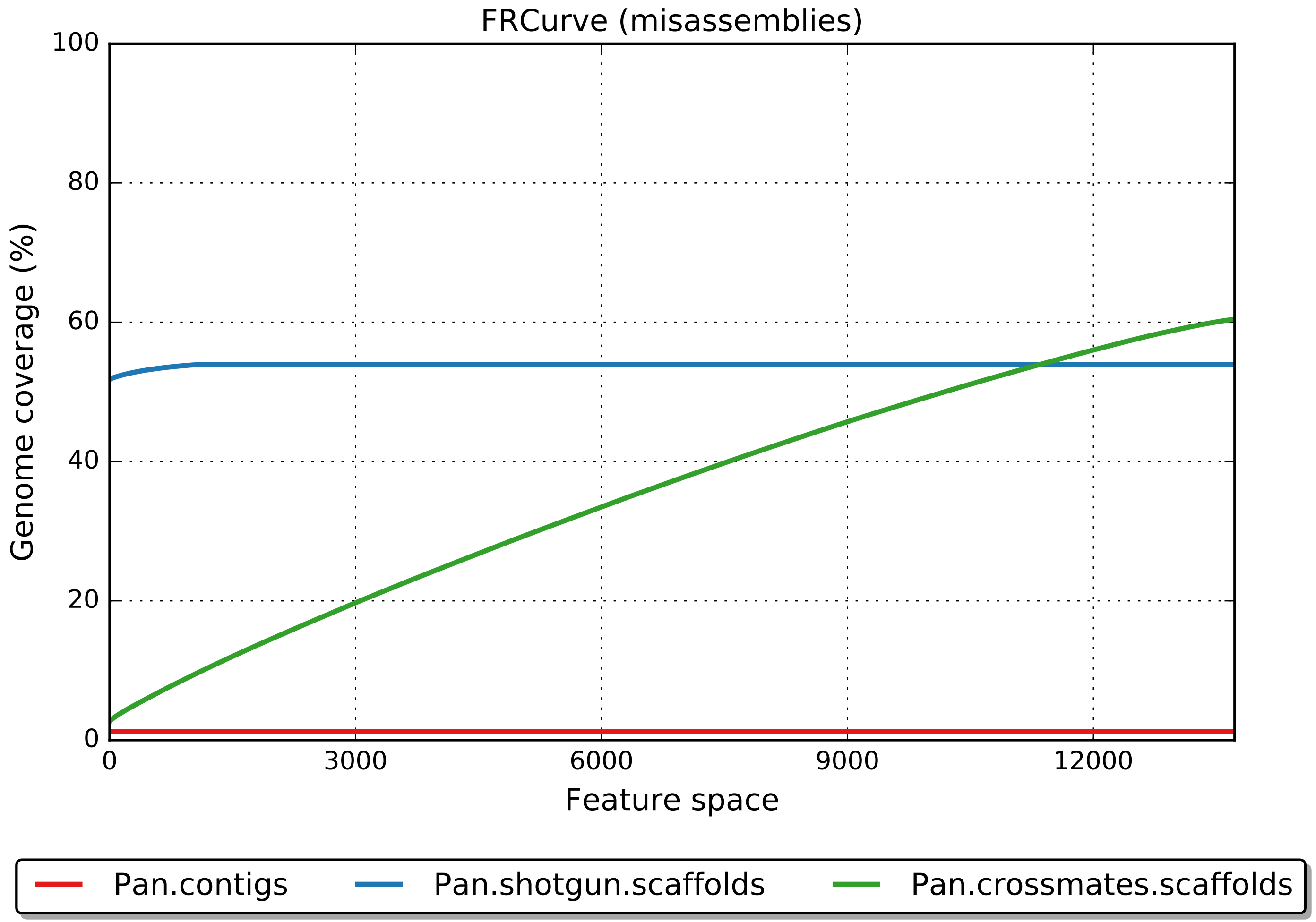
FRCurve (misassemblies)
Genome coverage (%)
100
80
60
40
20
0
0
3000
6000
9000
12000
Feature space
Pan.contigs
Pan.shotgun.scaffolds
Pan.crossmates.scaffolds

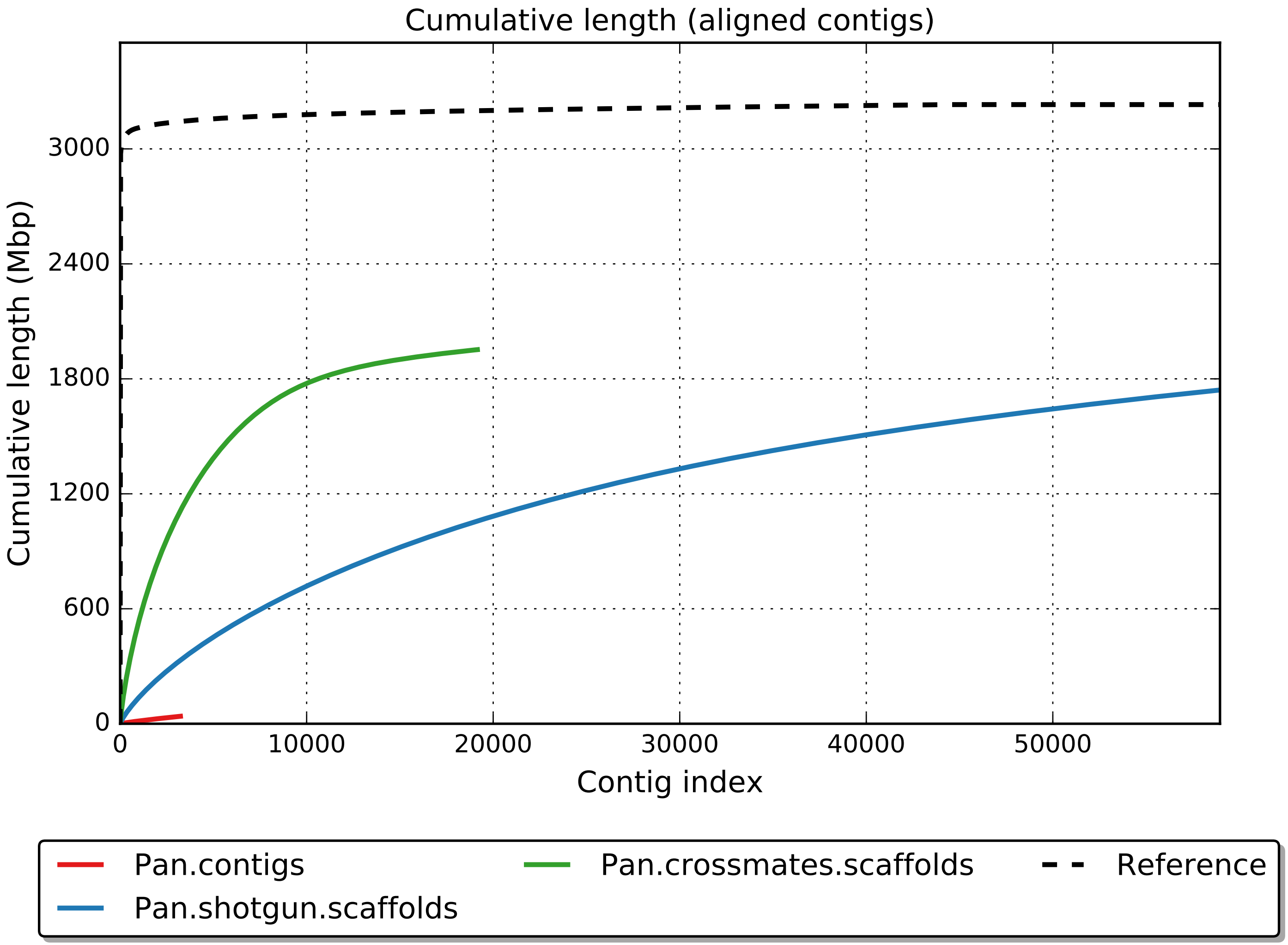
Cumulative length (aligned contigs)
Cumulative length (Mbp)
0
600
1200
1800
2400
3000
0
10000
20000
30000
40000
50000
Contig index
Pan.contigs
Pan.shotgun.scaffolds
Pan.crossmates.scaffolds
Reference

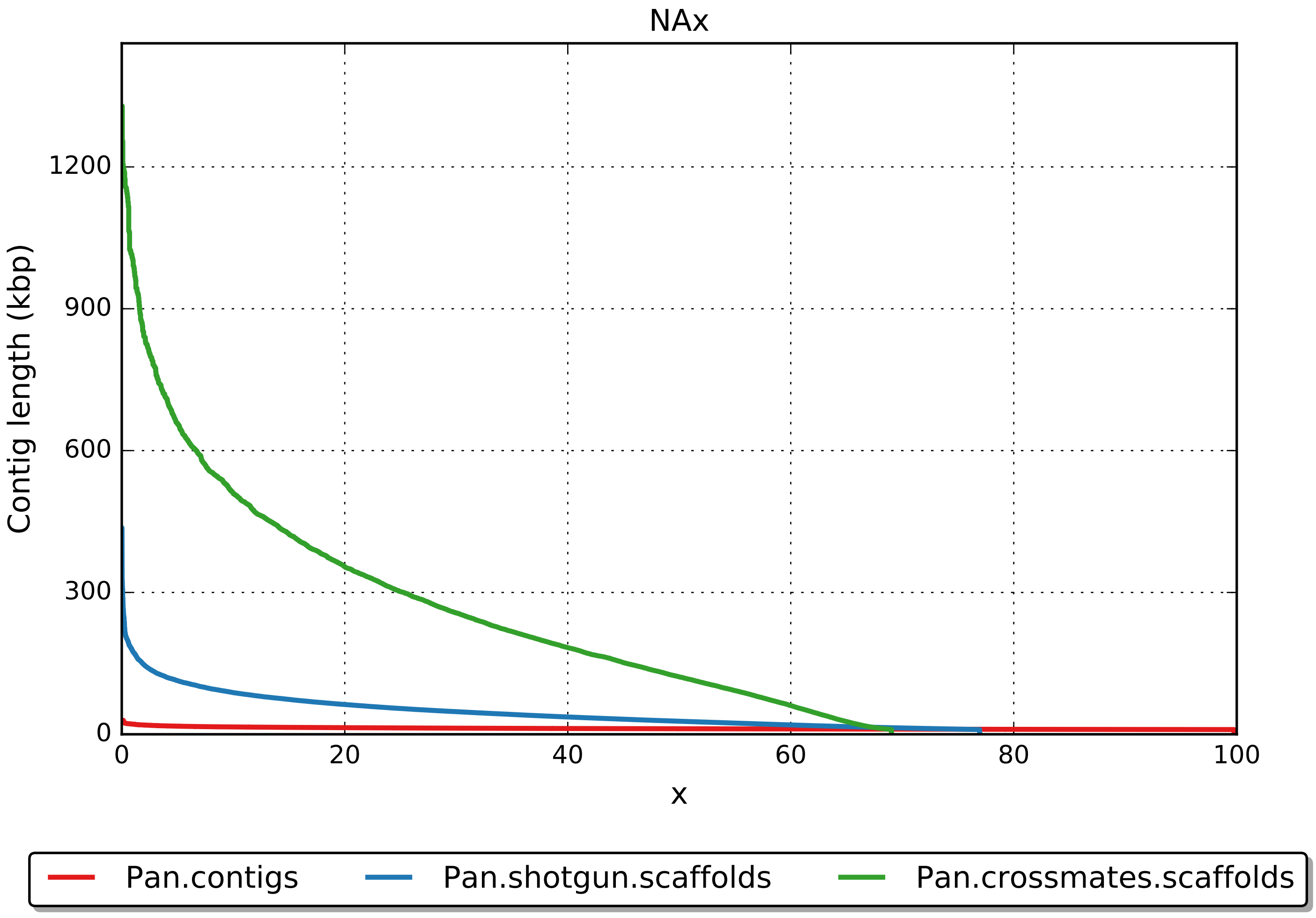
NAx
Contig length (kbp)
1200
900
600
300
0
0
20
40
60
80
100
x
Pan.contigs
Pan.shotgun.scaffolds
Pan.crossmates.scaffolds

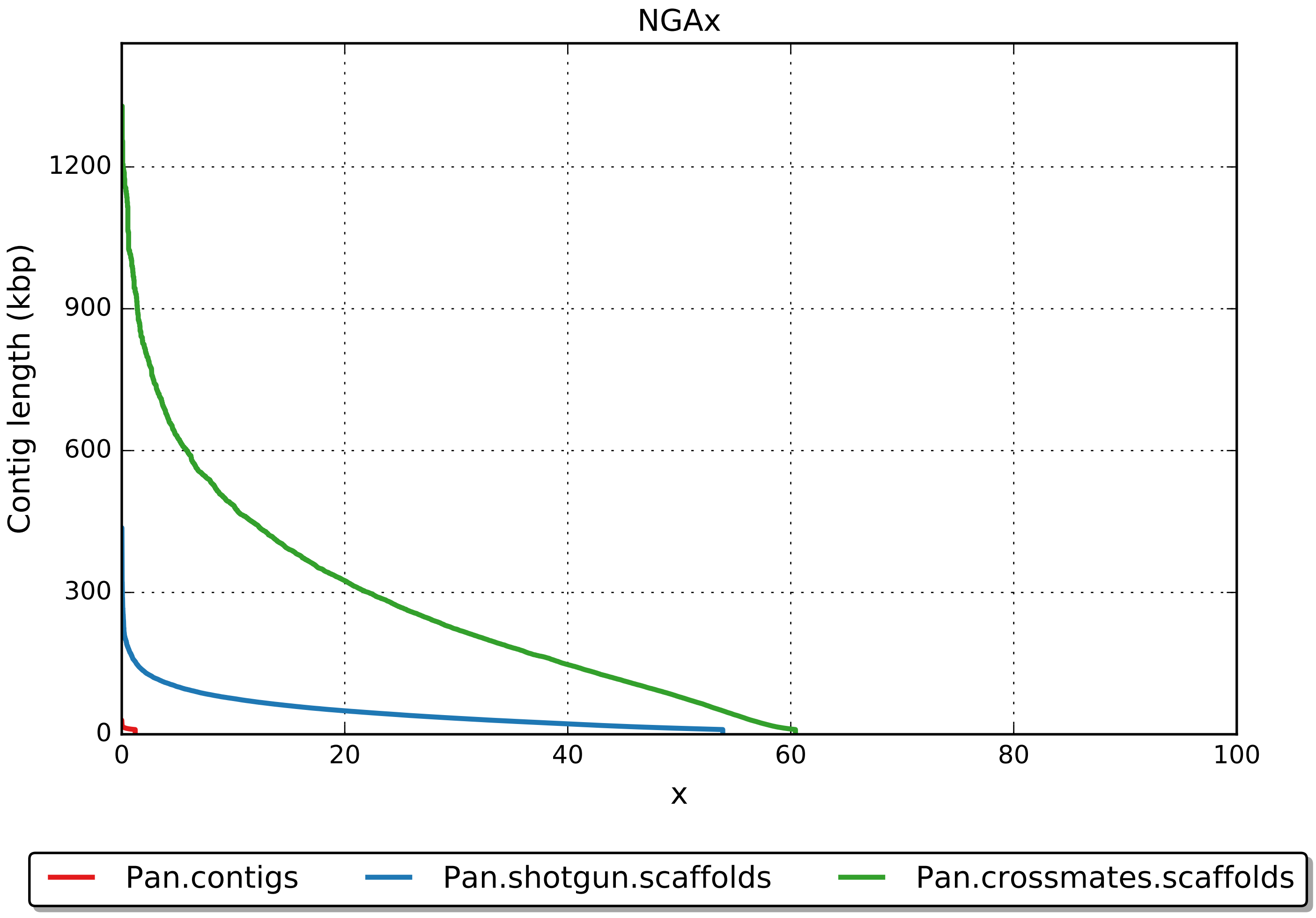
NGAx
Contig length (kbp)
1200
900
600
300
0
0
20
40
60
80
100
x
Pan.contigs
Pan.shotgun.scaffolds
Pan.crossmates.scaffolds

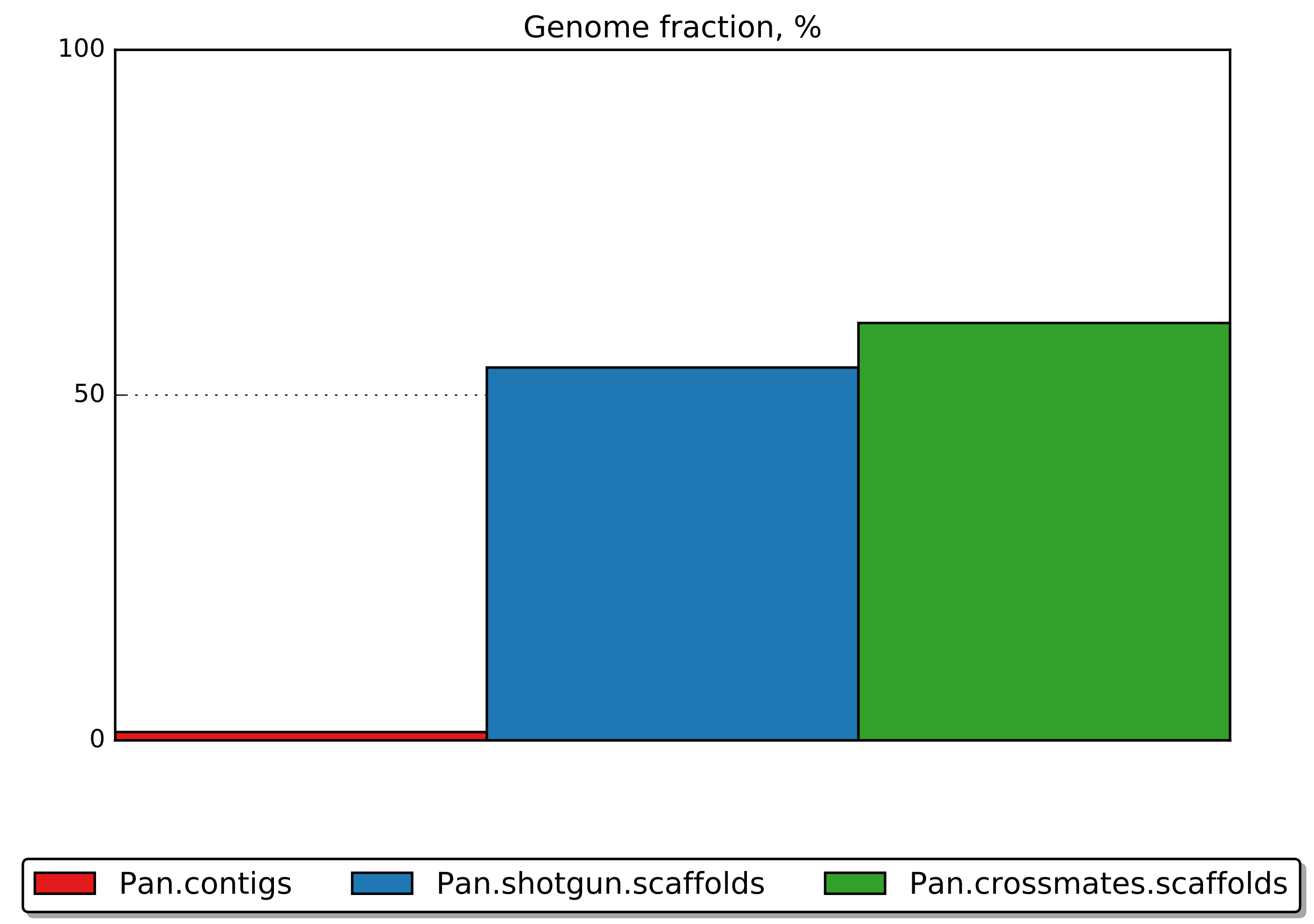

Genome fraction, %
100
50
0
Pan.contigs
Pan.shotgun.scaffolds
Pan.crossmates.scaffolds
