# Supplementary material for: Improving draft genome contiguity with reference-derived in silico mate-pair libraries
Source: Gigascience. 2018 Apr 21;7(5):giy029. doi: 10.1093/gigascience/giy029 (PMC5967465; doi:10.1093/gigascience/giy029)

Additional File 1

Improving draft genome contiguity with reference-derived *in silico* mate-pair libraries

José Horacio Grau [1ǂ], Thomas Hackl [2ǂ], Klaus-Peter Koepfli [3,4], Michael Hofreiter [5].

[1] Museum für Naturkunde Berlin, Leibniz-Institut für Evolutions- und Biodiversitätsforschung an der Humboldt-Universität zu Berlin. Invalidenstraße 43, 10115. Berlin, Germany.
[2] Massachusetts Institute of Technology, Department of Civil and Environmental Engineering, 15 Vassar Street, Cambridge, MA, 02139. USA.
[3] Smithsonian Conservation Biology Institute, National Zoological Park, 3001 Connecticut Avenue NW, Washington, D.C. 20008. USA.
[4] Theodosius Dobzhansky Center for Genome Bioinformatics, St. Petersburg State University, Sredniy Prospekt 41A, St. Petersburg, 199004. Russia.
[5] Faculty of Mathematics and Life Sciences, Institute of Biochemistry and Biology, Unit of General Zoology–Evolutionary Adaptive Genomics, University of Potsdam, Karl-Liebknecht-Straße 24-25, 14476 Potsdam, Germany.
[ǂ] Authors contributed equally

Text S1) Cross-mates command line example for tapeworm experiment.
The following command calls cross-mates script to use produce a consensus fastq by mapping a paired-end library from *Taenia solium* (SRR524725) on the reference genome assembly of *Taenia asiatica* (GCA_001693035.2_Taenia_asiatica_TASYD01_v1_genomic), and subsequently produce *in silico* scaffolding libraries of 50 bp length at a 10X coverage. The -z and -s switch on gzipped fastq files and output preliminary configuration file for SOAPdenovo assembler. The different insert sizes are specified with the -i switch:

```
./cross-species-scaffolding/bin/cross-mates \
GCA_001693035.2_Taenia_asiatica_TASYD01_v1_genomic \
SRR524725_1.fastq.gz SRR524725_2.fastq.gz \
-t 20 -l 50 -c 10 -z -s -i 500,1000,1500,2000,5000,10000,20000,50000,100000,200000
```

**Table S1) Genome size and multiplicity of 23-mers by KrATER for the paired-end datasets used to generate the *in silico* mate pair libraries.**					
		Pan troglodytes SRP012268	*Daubentonia madagascariensis* SRP007603	*Taenia solium* SRR524725	*Saccharomyces cerevisiae* CLIB324
	Plot	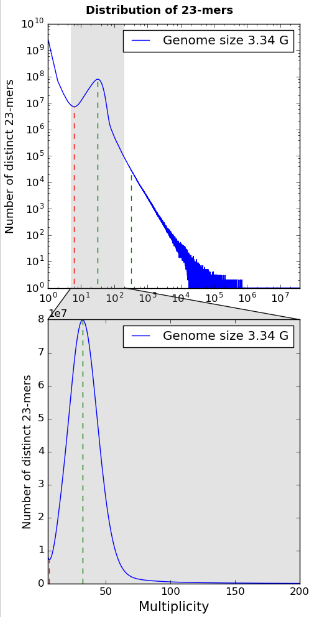	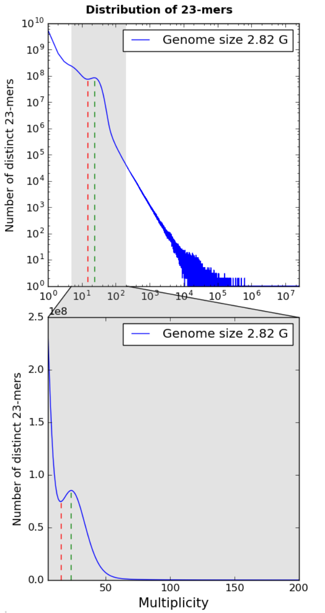	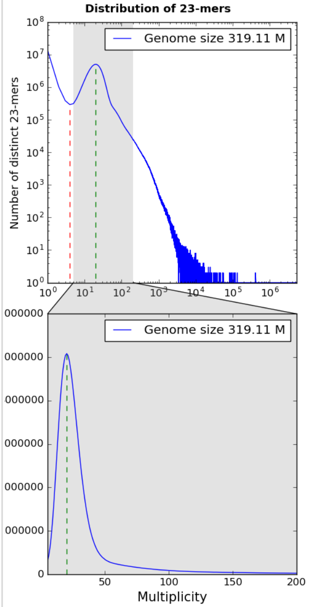	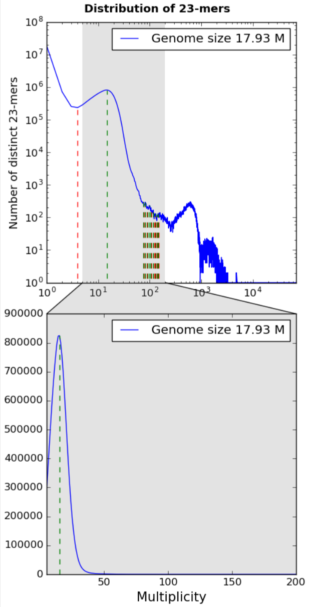
	Number of distinct kmers	5,378,036,130	10,242,942,794	138,892,216	32,787,741
	Number of distinct kmers with errors	2,990,084,768	8,295,490,890	15,334,880	20,054,474
	Fraction of distinct kmers with errors	1	1	0	1
	Total number of kmers	110,174,286,172	86,016,577,465	6,400,447,389	291,194,337
	Total number of kmers with errors	3,169,243,606	20,009,811,283	17,149,342	21,290,323
	Fraction of kmers with errors	0	0	0	0
	Kmer multiplicity at first minimum	6	15	4	4
	Kmer multiplicity at first maximum	32	23	20	15
	Width of first peak	55	15	50	21
	Mean kmer multiplicity in first peak	33	22	23	14
	Standard deviation of kmer multiplicity in first peak	11	4	9	5
	Variance coefficient of kmer multiplicity in first peak	0	0	0	0
	Estimated genome size, bp	3,342,555,746	2,821,173,432	319,106,216 *	17,930,250
	* Genome size estimation of *Taenia* not accurate because of high degree of heterozygosity.				

Table S2) Assembly metrics and BUSCO scores of Chimpanzee and Aye-aye before and after scaffolding with in silico mate pair libraries (libs).

Species	*Pan troglodytes*	*Pan troglodytes*	*Pan troglodytes*	*Daubentonia madagascariensis*	*Daubentonia madagascariensis*	*Daubentonia madagascariensis*	*Daubentonia madagascariensis*
Assembly	RERFERENCE GCF_000001515.7_Pan_tro_3.0_genomic	Chimp no scaffolding libs	Chimp with scaffolding libs from Human	RERFERENCE GCA_000241425.1 DauMad_1.0	Aye-aye no scaffolding libs	Aye-aye with scaffolding libs from Human	Aye-aye with scaffolding libs from Mouse lemur
References for *in silico* mate pairs			Human			Human	Mouse lemur
# contigs (>= 1000 bp)	44,437	166,796	22,391	499,368	312,253	283,226	101,520
# contigs (>= 5000 bp)	6,337	101,771	2,201	130,095	143,004	126,586	26,715
# contigs (>= 10000 bp)	3,086	71,696	1,234	34,769	69,736	75,093	17,424
# contigs (>= 25000 bp)	1,028	32,575	820	1,195	10,607	28,610	12,595
# contigs (>= 50000 bp)	616	11,431	723	5	634	9,717	9,569
Total length (>= 0 bp)	3,231,170,666	2,726,360,032	2,947,169,439	2,855,365,987	3,196,808,300	3,869,477,297	3,414,194,764
Total length (>= 1000 bp)	3,231,158,757	2,639,881,281	2,870,750,141	2,050,547,627	2,197,219,134	2,908,394,768	2,437,644,047
Total length (>= 5000 bp)	3,164,768,919	2,481,733,589	2,834,595,366	1,155,352,725	1,775,435,987	2,538,555,576	2,282,526,226
Total length (>= 10000 bp)	3,141,974,501	2,263,681,491	2,828,034,793	497,635,767	1,250,962,806	2,173,147,166	2,218,957,858
Total length (>= 25000 bp)	3,111,504,067	1,632,215,540	2,821,935,846	35,751,424	357,996,146	1,436,457,581	2,143,694,566
Total length (>= 50000 bp)	3,097,759,375	892,232,735	2,818,657,214	294,164	38,164,514	780,238,007	2,033,422,285
# contigs	44,449	543,105	387,322	3,231,305	5,460,621	5,386,719	5,368,402
Largest contig	228,573,443	437,303	32,950,256	86,436	125,674	468,047	3,769,618
Total length	3,231,170,666	2,726,360,032	2,947,169,439	2,855,365,987	3,196,808,300	3,869,477,297	3,414,194,764
GC (%)	41	41	41	40	39	39	39
N50	135,926,727	32,703	9,012,513	3,653	6,622	14,332	118,481
N75	83,230,942	14,903	4,695,019	605	371	1,052	410
L50	10	23,158	94	193,780	112,334	54,955	5,465
L75	17	53,872	205	615,366	705,827	277,097	320,137
# N's per 100 kbp	3,050	577	5,866	260	3,008	16,949	6,956
Complete BUSCOs	2,468	1,474	2,456	285	624	1,035	1,727
Complete and single-copy BUSCOs	2,329	1,438	2,397	277	614	1,013	1,705
Complete and duplicated BUSCOs	139	36	59	8	10	22	22
Fragmented BUSCOs	375	812	377	596	801	867	722
Missing BUSCOs	180	737	190	2,142	1,598	1,121	574
Total BUSCO groups searched	3,023	3,023	3,023	3,023	3,023	3,023	3,023
**Reference genomes**							
Human		*Homo sapiens (GRCh38.p8; GCF_000001405)*					
Mouse lemur		*Microcebus murinus (Mmur_2.0; GCF_000165445)*					

Table S3) Assembly metrics and BUSCO scores of pork tapeworm (*Taenia solium*) before and after scaffolding with *in silico* mate pair libraries (libs) sampled from reference genomes of four species of tapeworm.

Species	*Taenia saginata*	*Taenia asiatica*	*Taenia multiceps*	*Taenia solium MEX*	*Taenia solium*	*Taenia solium*
Assembly	REFERENCE GCA_001693075.2_ASM169307v2_genomic	REFERENCE GCA_001693035.2_Taenia_asiatica_TASYD01_v1_genomic	REFERENCE GCA_001923025.1_T_multiceps_v1.0_genomic	REFERENCE GCA_001870725.1_MEX_genome_complete.1-6-13_genomic	*Taenia solium* no scaffolding libs	*Taenia solium* with scaffolding libs from 4 reference genomes
References for *in silico* mate pairs						*Taenia saginata, T. asiatica, T.multiceps, T. solium*
# contigs (>= 1000 bp)	3,344	5,466	5,527	7,145	14,841	3,758
# contigs (>= 5000 bp)	1,726	2,481	5,125	2,042	6,446	750
# contigs (>= 10000 bp)	1,255	1,670	4,091	1,279	3,336	458
# contigs (>= 25000 bp)	813	923	1,259	736	671	295
# contigs (>= 50000 bp)	524	495	499	500	70	249
Total length (>= 0 bp)	169,104,283	168,679,183	240,089,990	129,810,970	121,307,316	148,598,691
Total length (>= 1000 bp)	168,922,558	167,682,211	240,089,990	126,704,980	107,197,346	136,670,994
Total length (>= 5000 bp)	164,866,497	161,418,450	238,582,817	115,490,774	87,520,159	130,971,385
Total length (>= 10000 bp)	161,543,453	155,746,626	230,761,979	110,193,047	65,412,772	128,982,996
Total length (>= 25000 bp)	154,223,960	143,770,124	184,391,633	101,641,387	24,347,633	126,457,451
Total length (>= 50000 bp)	143,829,057	128,802,785	159,279,070	93,446,729	4,325,040	124,896,203
# contigs	3,626	6,900	5,527	13,131	73,109	57,520
Largest contig	7,334,011	4,220,646	10,500,003	1,802,680	139,370	5,891,131
Total length	169,104,283	168,679,183	240,089,990	129,810,970	121,307,316	148,598,691
GC (%)	43	43	44	43	43	43
N50	586,235	342,420	275,186	165,960	11,276	907,969
N75	94,827	55,803	27,254	35,619	4,193	207,346
L50	65	97	114	192	2,890	39
L75	280	452	1,094	593	7,200	120
# N's per 100 kbp	1,650	2,533	0	36	30	10,169
Complete BUSCOs	290	297	296	274	243	278
Complete and single-copy BUSCOs	274	277	275	258	228	259
Complete and duplicated BUSCOs	16	20	21	16	15	19
Fragmented BUSCOs	170	158	160	163	172	171
Missing BUSCOs	383	388	387	406	428	394
Total BUSCO groups searched	843	843	843	843	843	843

Table S4) Assembly metrics and BUSCO scores of yeast before and after scaffolding with in silico mate pair libraries (libs).

Species	*Saccharomyces cerevisiae*	*Saccharomyces cerevisiae*	*Saccharomyces cerevisiae*
Assembly	REFERENCE NC_001133.9 Saccharomyces cerevisiae S288c	SPADES no scaffolding libs	SPADES with scaffolding libs
Reference for *in silico* mate-pairs			NC_001133.9 Saccharomyces cerevisiae S288c
# contigs (>= 1000 bp)	17	634	174
# contigs (>= 5000 bp)	17	421	54
# contigs (>= 10000 bp)	17	326	31
# contigs (>= 25000 bp)	17	162	27
# contigs (>= 50000 bp)	17	47	26
Total length (>= 0 bp)	12,157,105	12,002,012	12,259,296
Total length (>= 1000 bp)	12,157,105	11,425,473	11,712,269
Total length (>= 5000 bp)	12,157,105	10,937,217	11,433,669
Total length (>= 10000 bp)	12,157,105	10,282,848	11,281,309
Total length (>= 25000 bp)	12,157,105	7,424,095	11,210,411
Total length (>= 50000 bp)	12,157,105	3,306,220	11,175,310
# contigs	17	2,804	2,074
Largest contig	1,531,933	122,700	1,026,282
Total length	12,157,105	11,947,045	12,127,960
GC (%)	38	38	38
N50	924,431	33,942	547,102
N75	666,816	17,927	279,367
L50	6	113	8
L75	10	233	15
# N's per 100 kbp	0	82	1,641
Complete BUSCOs	1,646	1,633	1,633
Complete and single-copy BUSCOs	1,607	1,599	1,596
Complete and duplicated BUSCOs	39	34	37
Fragmented BUSCOs	58	67	68
Missing BUSCOs	7	11	10
Total BUSCO groups searched	1,711	1,711	1,711

Table S5) Runtimes of cross-mate pipeline sub-commands for the generation of the chimp consensus genome

The pipeline was run on a 20 CPU HPC cluster node; Intel(R) Xeon(R) CPU E5-2650 v3 @ 2.30GHz; 256 GB RAM

Command	CPUs (effectively used)	Runtime [hh:mm:ss]	Runtime [s]
bwa index	1	1:16:34	4594
bwa mem | samtools view | samtools sort	20	9:18:37	33517
samtools index	1	16:49	1009
samtools mpileup | bcftools | vcfutils | seqtk	2	30:34:05	110045
**Total**		**41:26:08**	**149165**

Figure S1) Runtime of *in silico* mate-pair library generation using seq-frag based on the chimp-human consensus genome with different insert sizes and target coverages.

Each point represents a consensus chromosome. Runtimes scale linearly with genome size and coverage, and are only slightly affected by larger insert sizes. Tests were performed on a customary laptop (Lenovo ThinkPad T460s, with Intel(R) Core(TM) i7-6600U CPU @ 2.60GHz, 32GB RAM and a 1TB SSD)

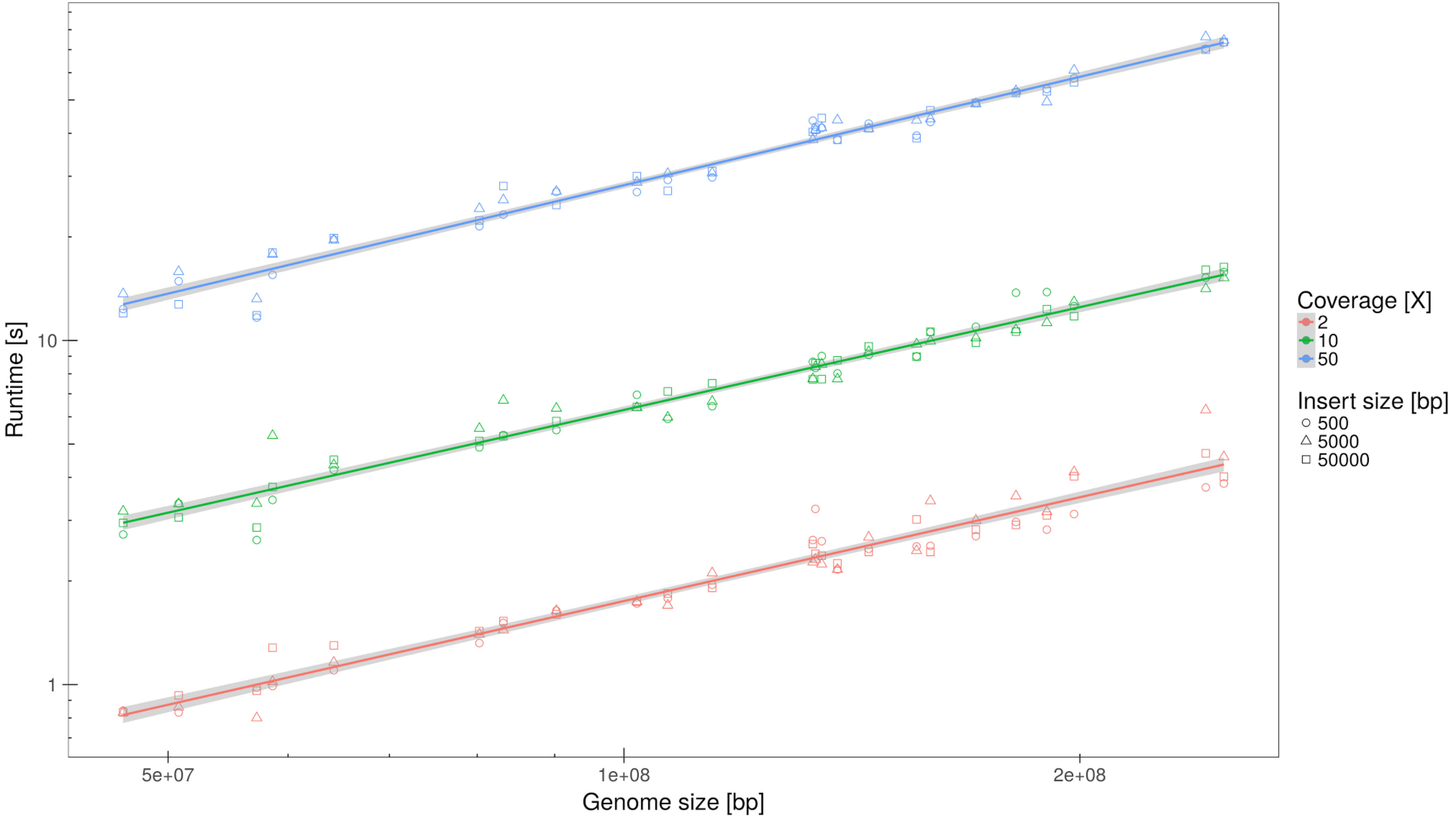

Supplement: Additional Files [file giy029_supp.zip › Supplementary1.pdf]
