# Supplementary material for: Improving draft genome contiguity with reference-derived in silico mate-pair libraries
Source: Gigascience. 2018 Apr 21;7(5):giy029. doi: 10.1093/gigascience/giy029 (PMC5967465; doi:10.1093/gigascience/giy029)
Supplement: Additional Files [file giy029_supp.zip › Taenia_report.pdf]

Report

	Taenia.contigs	Taenia.shotgun.scaffolds	Taenia.crossmates.scaffolds
# contigs (>= 0 bp)	221144	73109	57520
# contigs (>= 1000 bp)	14669	14841	3758
# contigs (>= 5000 bp)	6471	6446	750
# contigs (>= 10000 bp)	3173	3336	458
# contigs (>= 25000 bp)	557	671	295
# contigs (>= 50000 bp)	45	70	249
Total length (>= 0 bp)	126290659	121307316	148598691
Total length (>= 1000 bp)	102994748	107197346	136670994
Total length (>= 5000 bp)	82975876	87520159	130971385
Total length (>= 10000 bp)	59516092	65412772	128982996
Total length (>= 25000 bp)	19492216	24347633	126457451
Total length (>= 50000 bp)	2673497	4325040	124896203
# contigs	3173	3336	458
Largest contig	113067	139370	5891131
Total length	59516092	65412772	128982996
Reference length	129810970	129810970	129810970
GC (%)	42.88	42.87	42.91
Reference GC (%)	43.00	43.00	43.00
N50	19567	20744	1140004
NG50	-	10178	1140004
N75	14110	14599	459206
NG75	-	-	451717
L50	1022	1039	29
LG50	-	3286	29
L75	1925	1987	74
LG75	-	-	76
# misassemblies	2	9	1031
# misassembled contigs	2	9	137
Misassembled contigs length	94018	331185	111076478
# local misassemblies	4	60	659
# unaligned mis. contigs	0	0	23
# unaligned contigs	11 + 37 part	169 + 380 part	135 + 221 part
Unaligned length	238167	3539145	28349077
Genome fraction (%)	45.633	47.580	74.345
Duplication ratio	1.001	1.002	1.043
# N's per 100 kbp	0.00	4.76	11656.53
# mismatches per 100 kbp	2.08	3.12	13.45
# indels per 100 kbp	0.24	0.63	2.99
Largest alignment	113067	139370	791542
Total aligned length	59270918	61815366	100634108
NA50	19490	19744	98008
NGA50	-	-	96833
NA75	13980	13503	15820
NGA75	-	-	14626
LA50	1027	1077	326
LGA50	-	-	331
LA75	1933	2087	1086
LGA75	-	-	1127

All statistics are based on contigs of size >= 10000 bp, unless otherwise noted (e.g., "# contigs (>= 0 bp)" and "Total length (>= 0 bp)" include all contigs).

Misassemblies report

	Taenia.contigs	Taenia.shotgun.scaffolds	Taenia.crossmates.scaffolds
# misassemblies	2	9	1031
# relocations	2	2	625
# translocations	0	7	393
# inversions	0	0	13
# misassembled contigs	2	9	137
Misassembled contigs length	94018	331185	111076478
# local misassemblies	4	60	659
# misassemblies caused by fragmented reference	1	4	278
# unaligned mis. contigs	0	0	23
# mismatches	1231	1925	12985
# indels	140	391	2885
# indels (<= 5 bp)	98	288	1763
# indels (> 5 bp)	42	103	1122
Indels length	1331	2817	31793

All statistics are based on contigs of size >= 10000 bp, unless otherwise noted (e.g., "# contigs (>= 0 bp)" and "Total length (>= 0 bp)" include all contigs).

Unaligned report

	Taenia.contigs	Taenia.shotgun.scaffolds	Taenia.crossmates.scaffolds
# fully unaligned contigs	11	169	135
Fully unaligned length	131267	2085240	6980348
# partially unaligned contigs	37	380	221
Partially unaligned length	106900	1453905	21368729
# N's	0	3111	15034942

All statistics are based on contigs of size >= 10000 bp, unless otherwise noted (e.g., "# contigs (>= 0 bp)" and "Total length (>= 0 bp)" include all contigs).

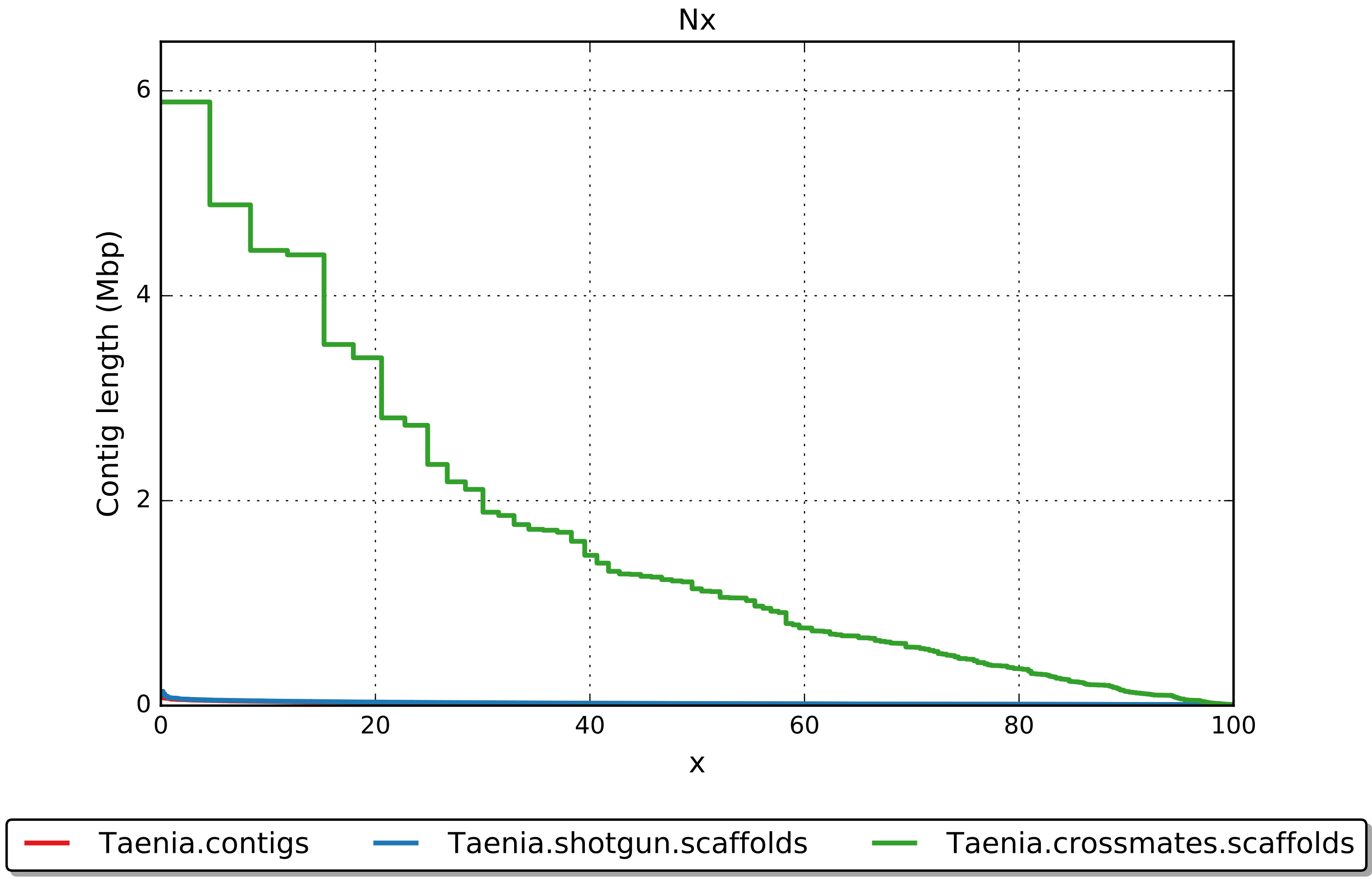
Nx
Contig length (Mbp)
0
2
4
6
0
20
40
60
80
100
x
Taenia.contigs
Taenia.shotgun.scaffolds
Taenia.crossmates.scaffolds

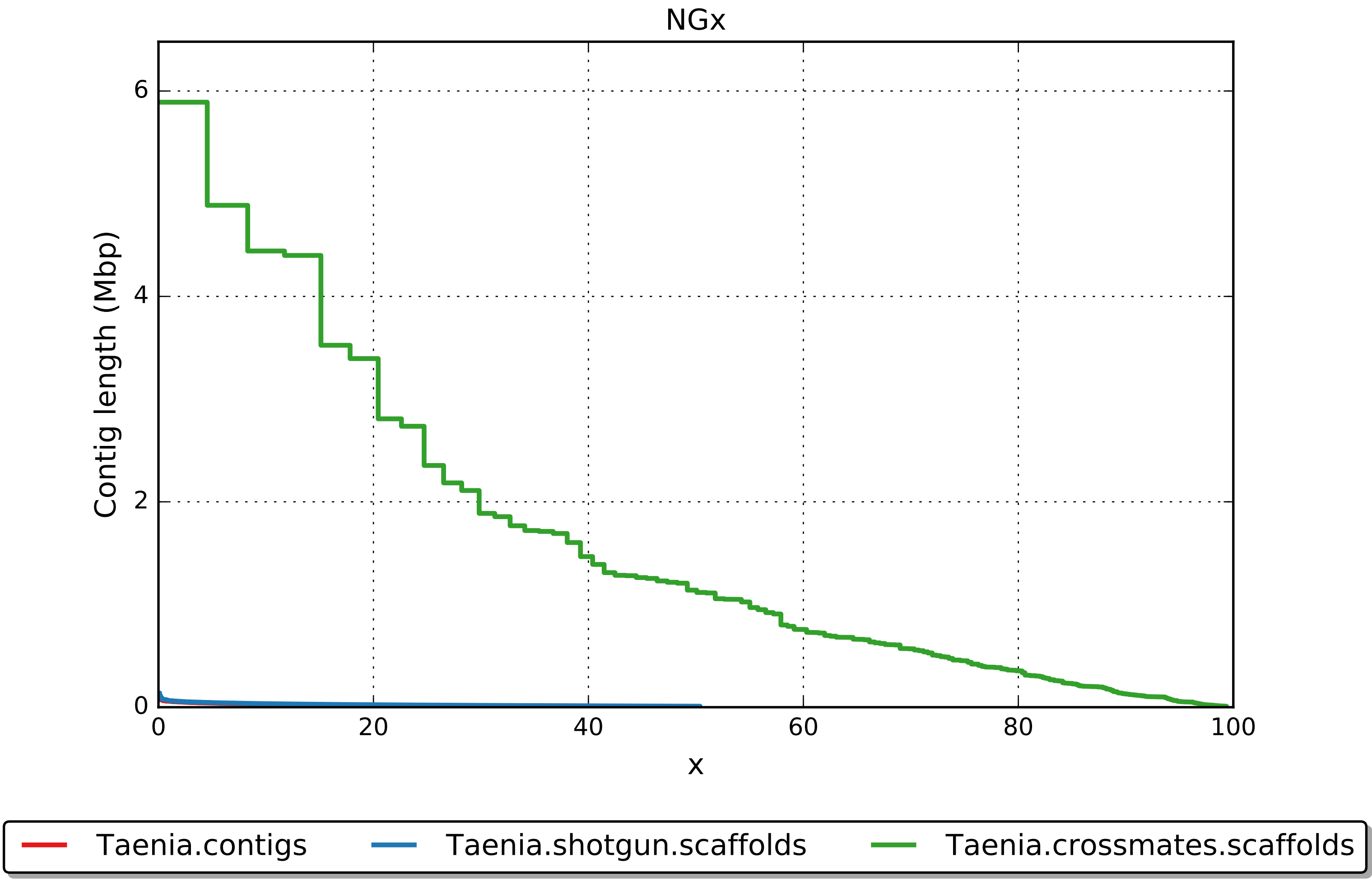

NGx
Contig length (Mbp)
6
4
2
0
0
20
40
60
80
100
x
Taenia.contigs
Taenia.shotgun.scaffolds
Taenia.crossmates.scaffolds

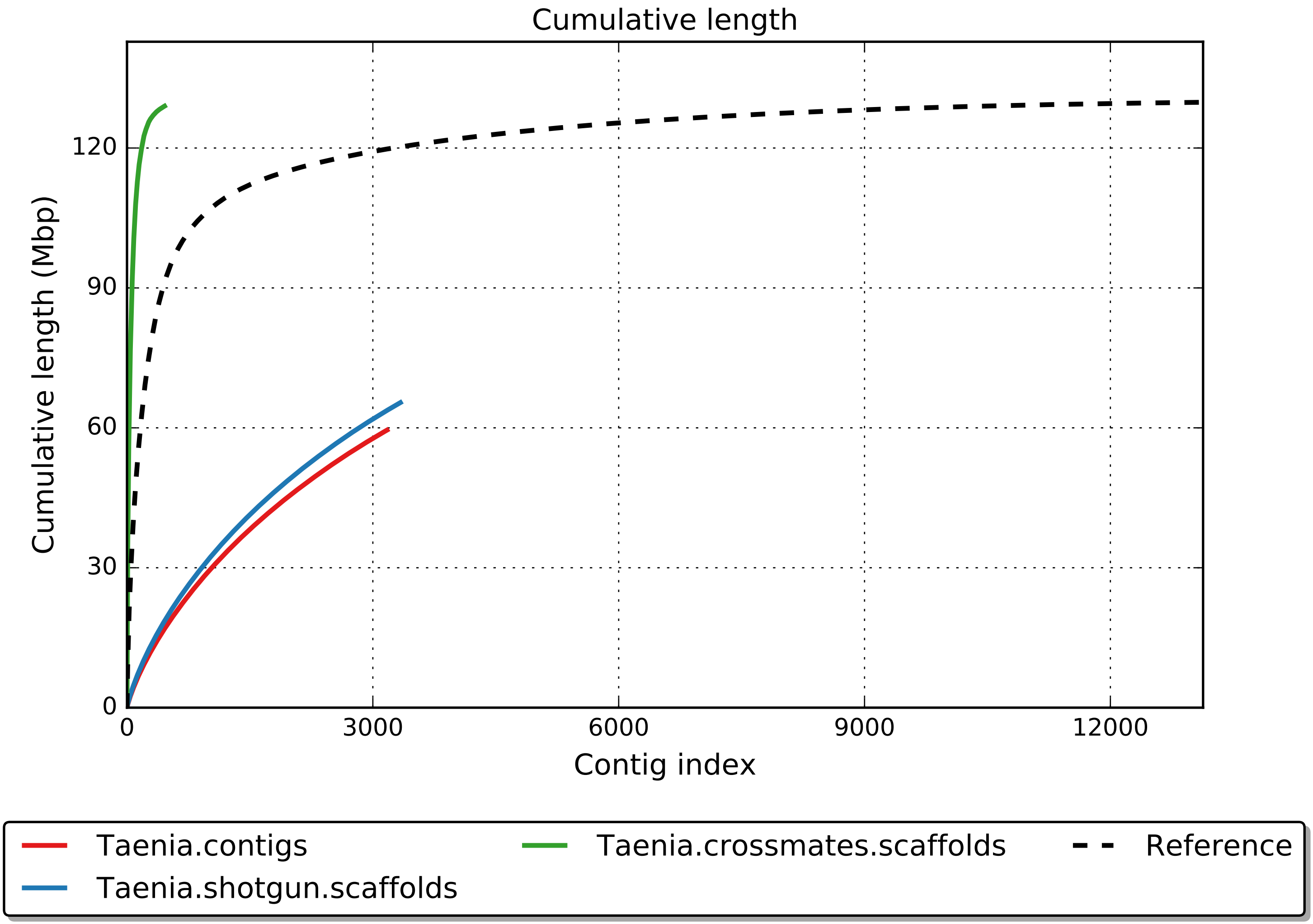
Cumulative length
Cumulative length (Mbp)
0
30
60
90
120
0
3000
6000
9000
12000
Contig index
Taenia.contigs
Taenia.crossmates.scaffolds
Reference
Taenia.shotgun.scaffolds

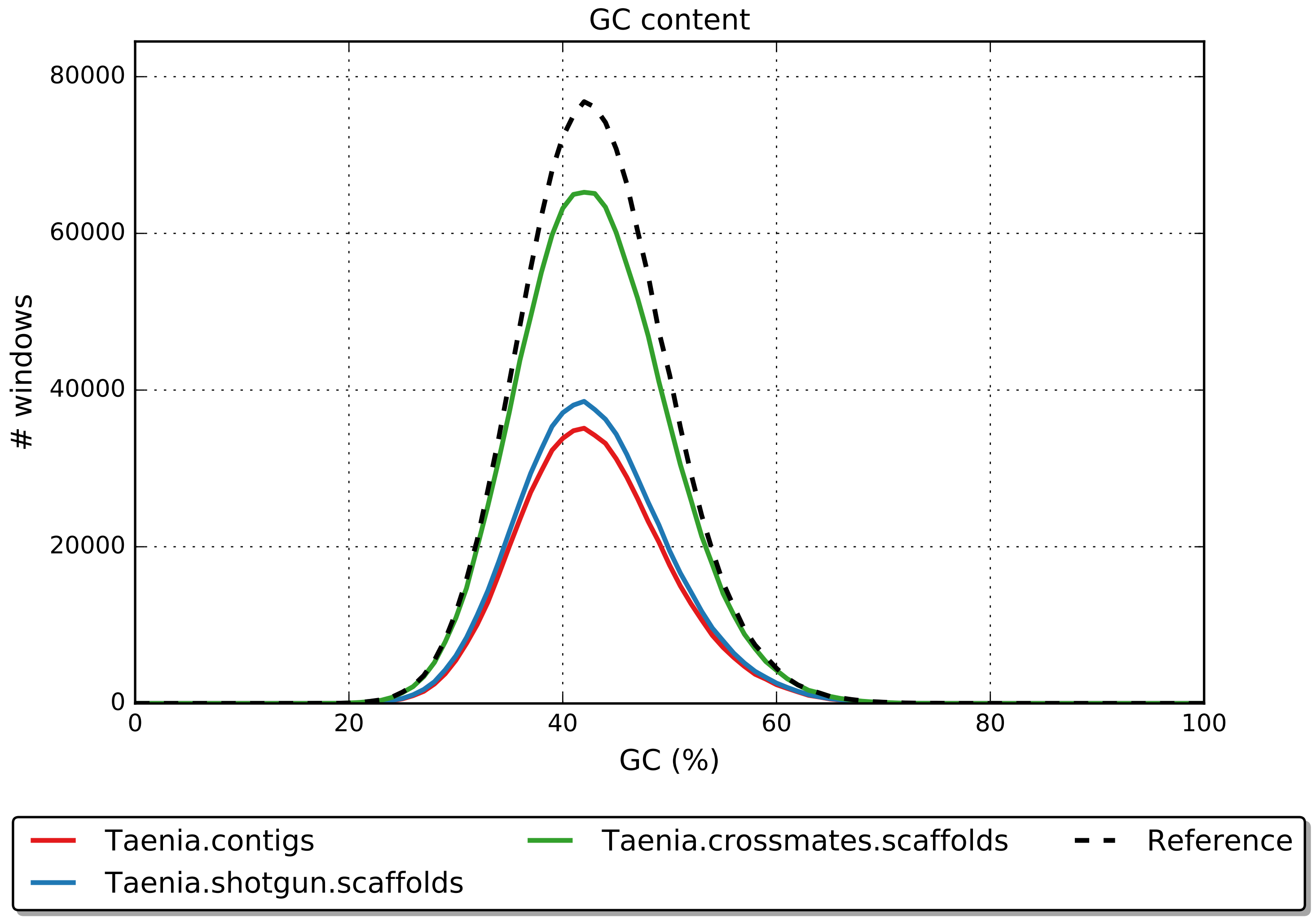
GC content
windows
GC (%)
0
20000
40000
60000
80000
0
20
40
60
80
100
Taenia.contigs
Taenia.shotgun.scaffolds
Taenia.crossmates.scaffolds
Reference

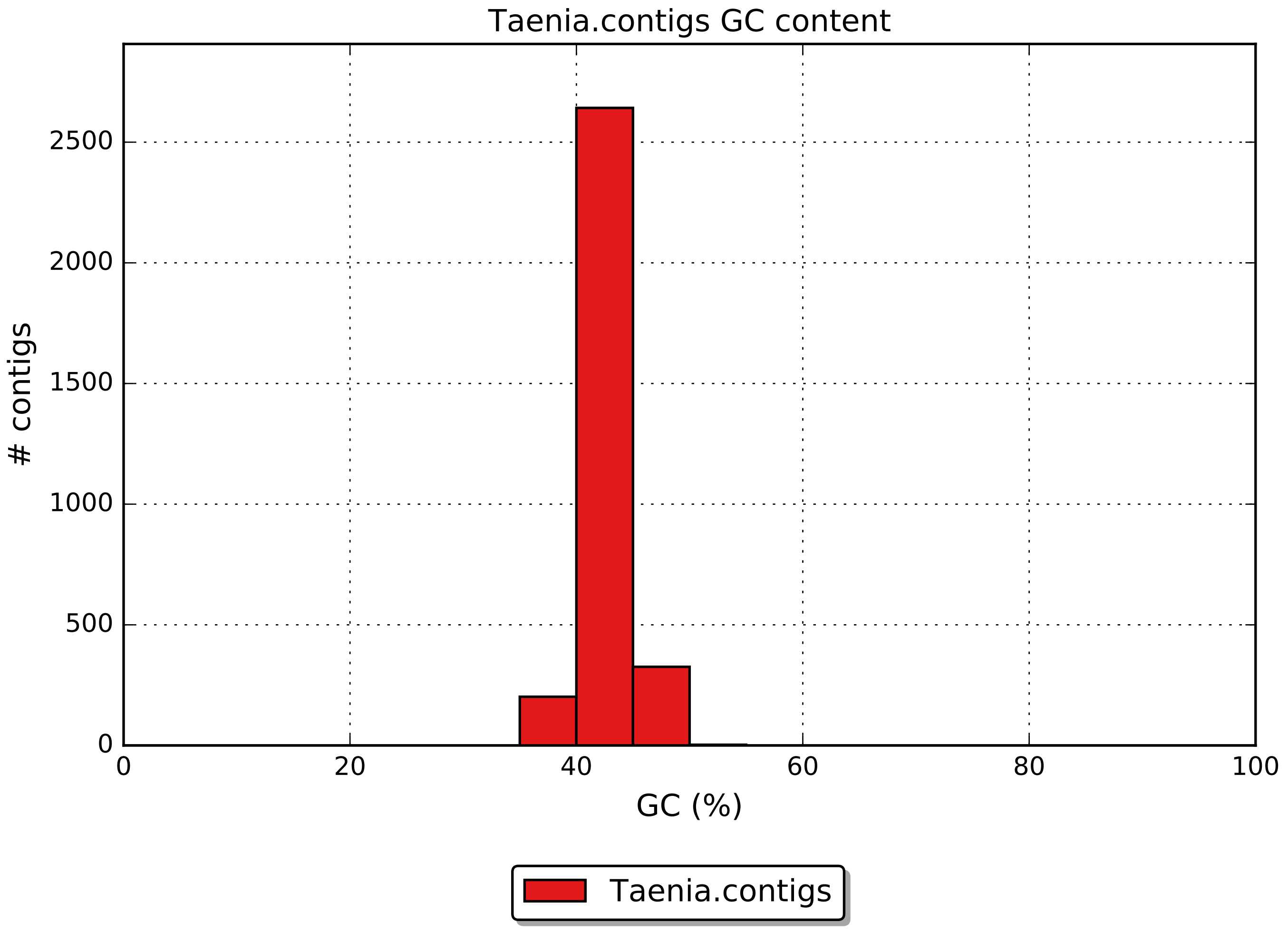

Taenia.contigs GC content
contigs
2500
2000
1500
1000
500
0
0
20
40
60
80
100
GC (%)
Taenia.contigs

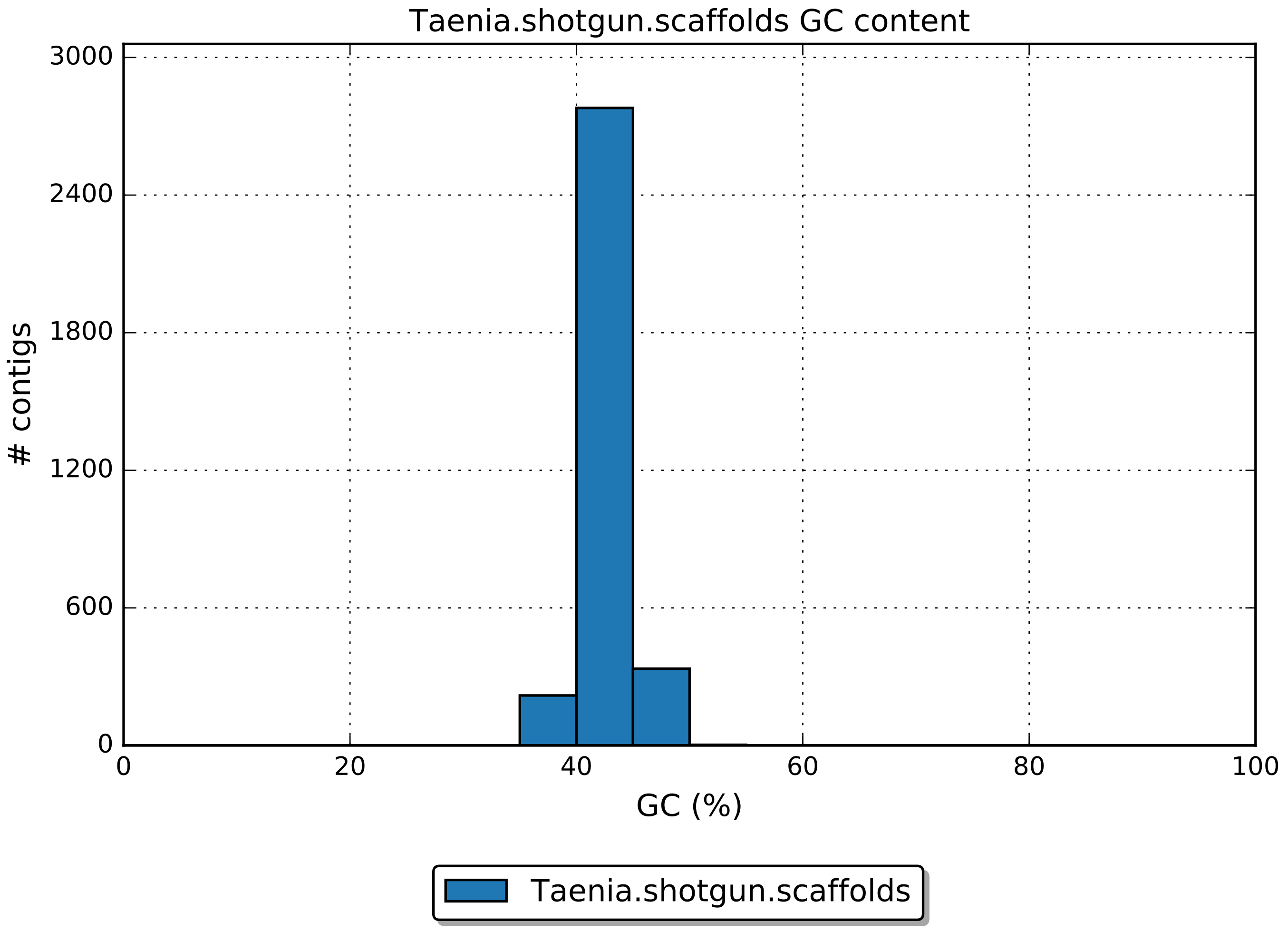

Taenia.shotgun.scaffolds GC content
contigs
3000
2400
1800
1200
600
0
0
20
40
60
80
100
GC (%)
Taenia.shotgun.scaffolds

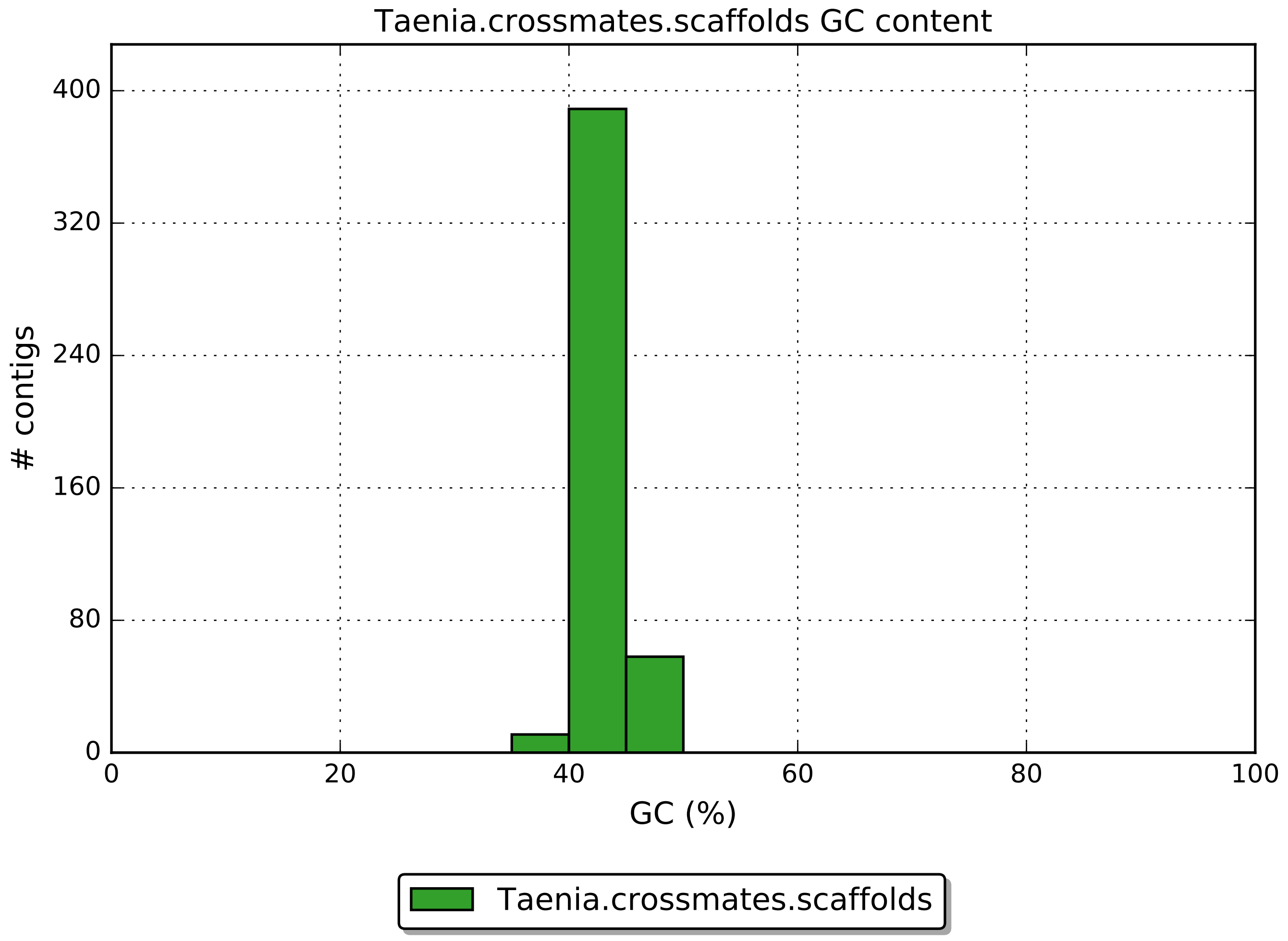
Taenia.crossmates.scaffolds GC content
contigs
0
80
160
240
320
400
0
20
40
60
80
100
GC (%)
Taenia.crossmates.scaffolds

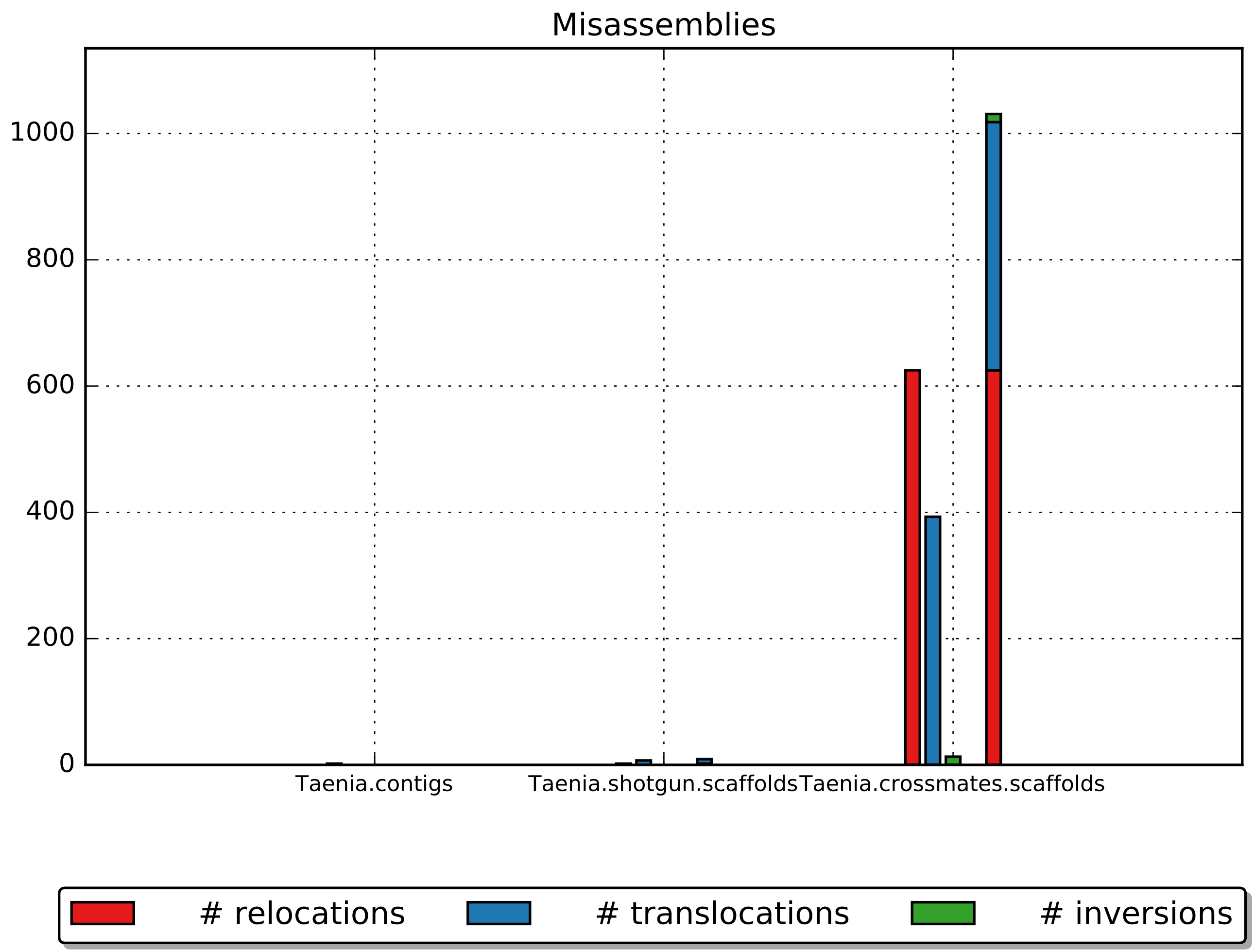
Misassemblies
0
200
400
600
800
1000
Taenia.contigs
Taenia.shotgun.scaffolds
Taenia.crossmates.scaffolds
relocations
translocations
inversions

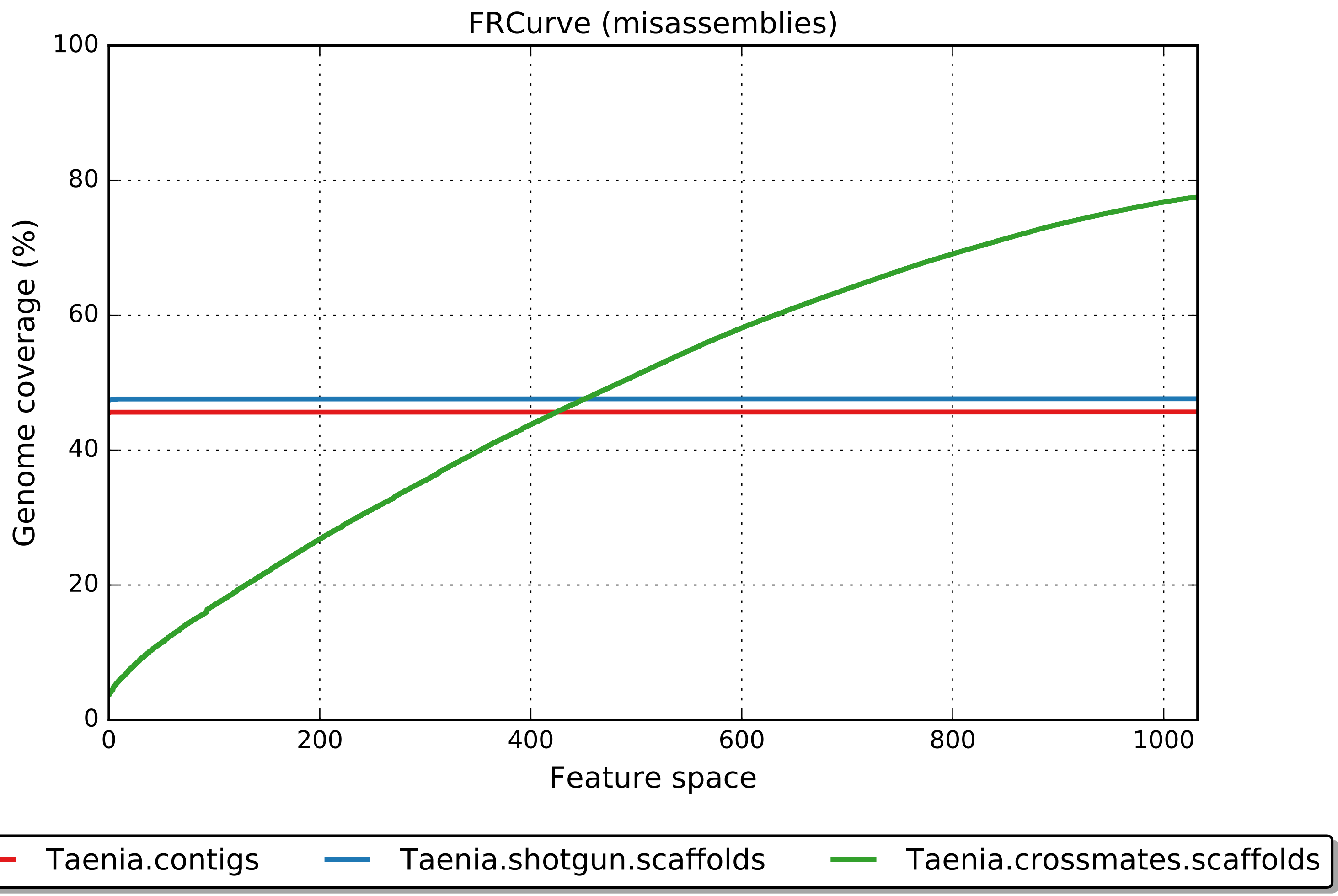
FRCurve (misassemblies)
Genome coverage (%)
Feature space
0
20
40
60
80
100
0
200
400
600
800
1000
Taenia.contigs
Taenia.shotgun.scaffolds
Taenia.crossmates.scaffolds

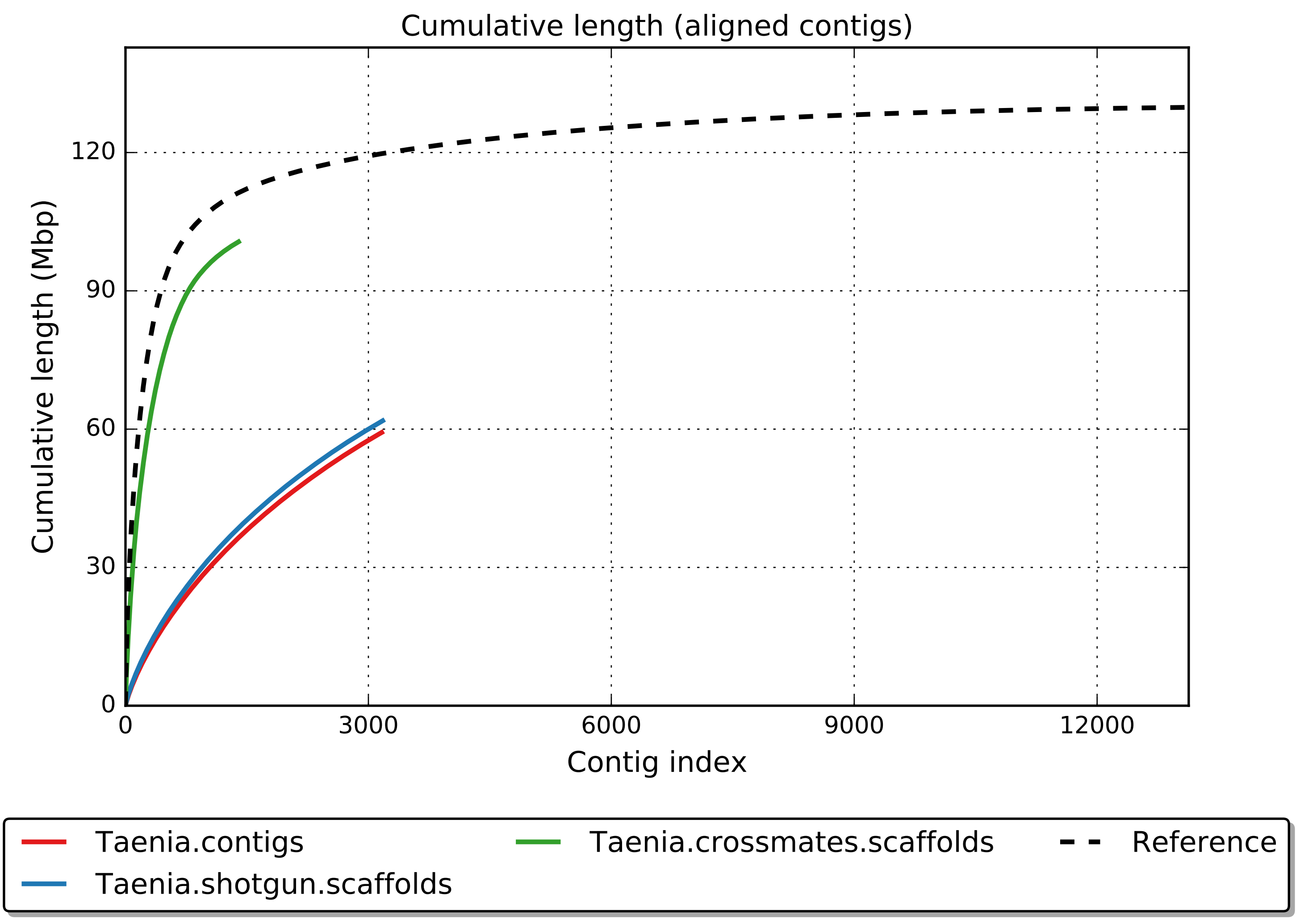
Cumulative length (aligned contigs)
Cumulative length (Mbp)
0
30
60
90
120
0
3000
6000
9000
12000
Contig index
Taenia.contigs
Taenia.shotgun.scaffolds
Taenia.crossmates.scaffolds
Reference

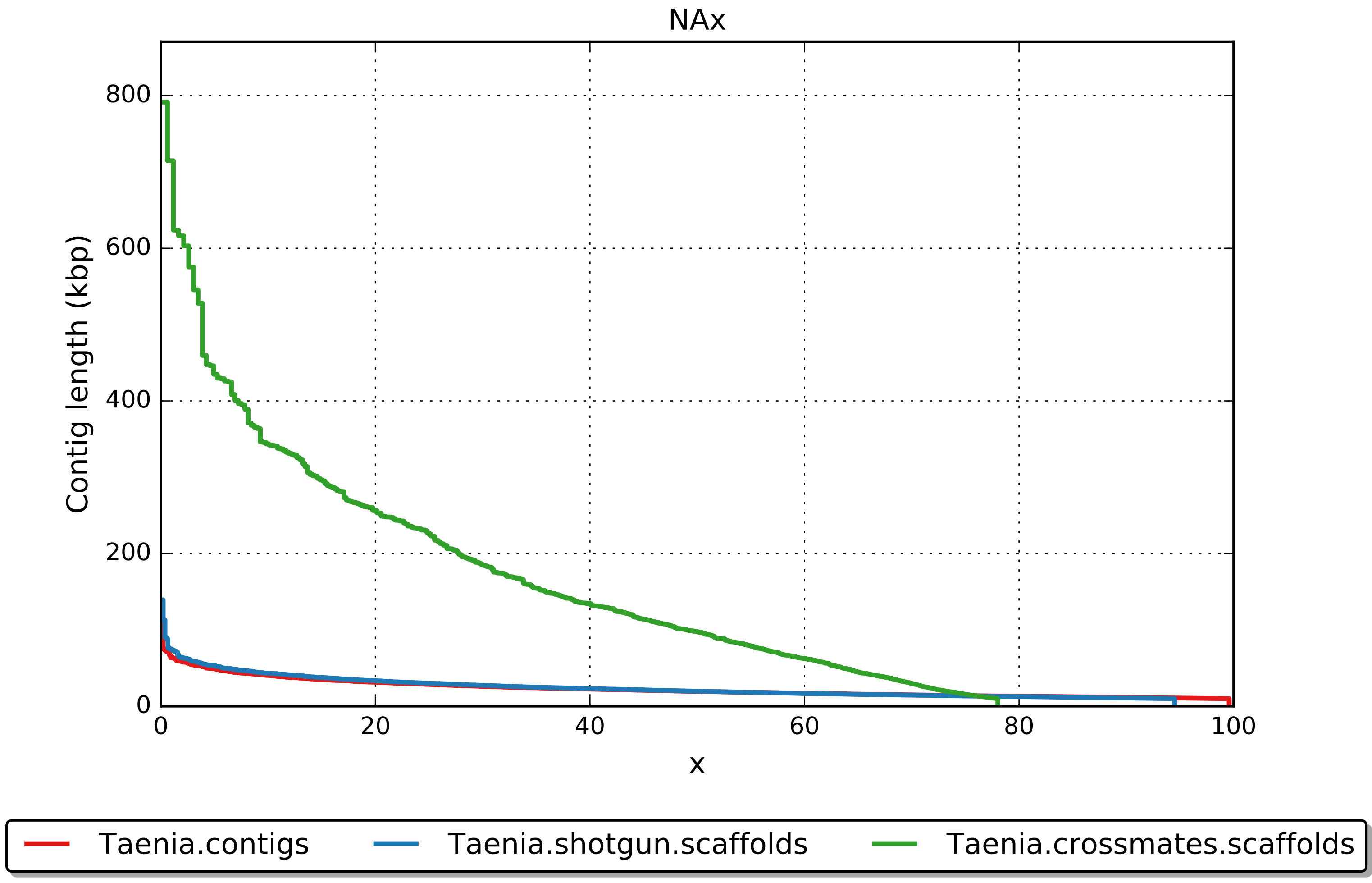
NAx
Contig length (kbp)
800
600
400
200
0
0
20
40
60
80
100
x
Taenia.contigs
Taenia.shotgun.scaffolds
Taenia.crossmates.scaffolds

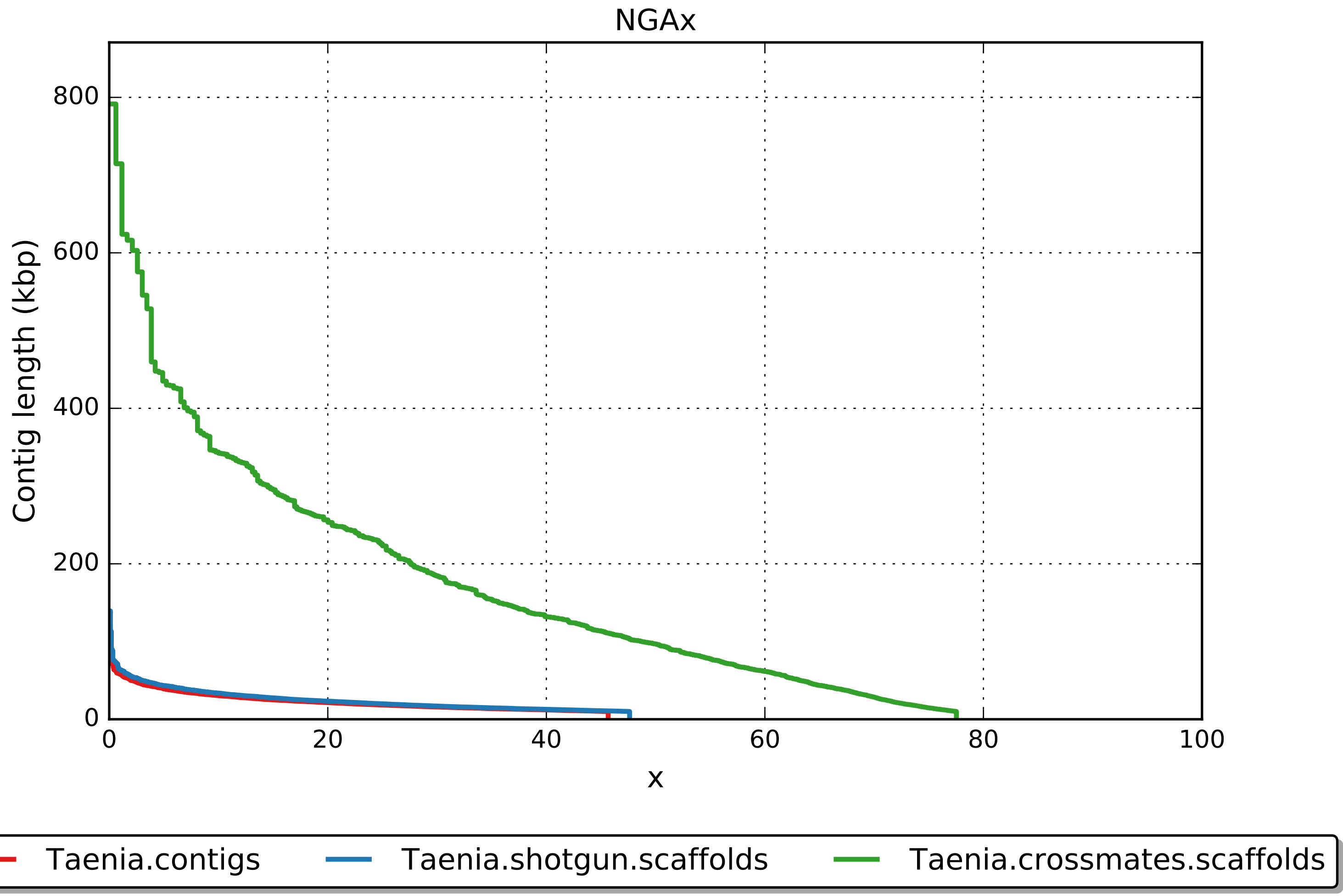
NGAx
800
600
400
200
0
Contig length (kbp)
0
20
40
60
80
100
x
Taenia.contigs
Taenia.shotgun.scaffolds
Taenia.crossmates.scaffolds

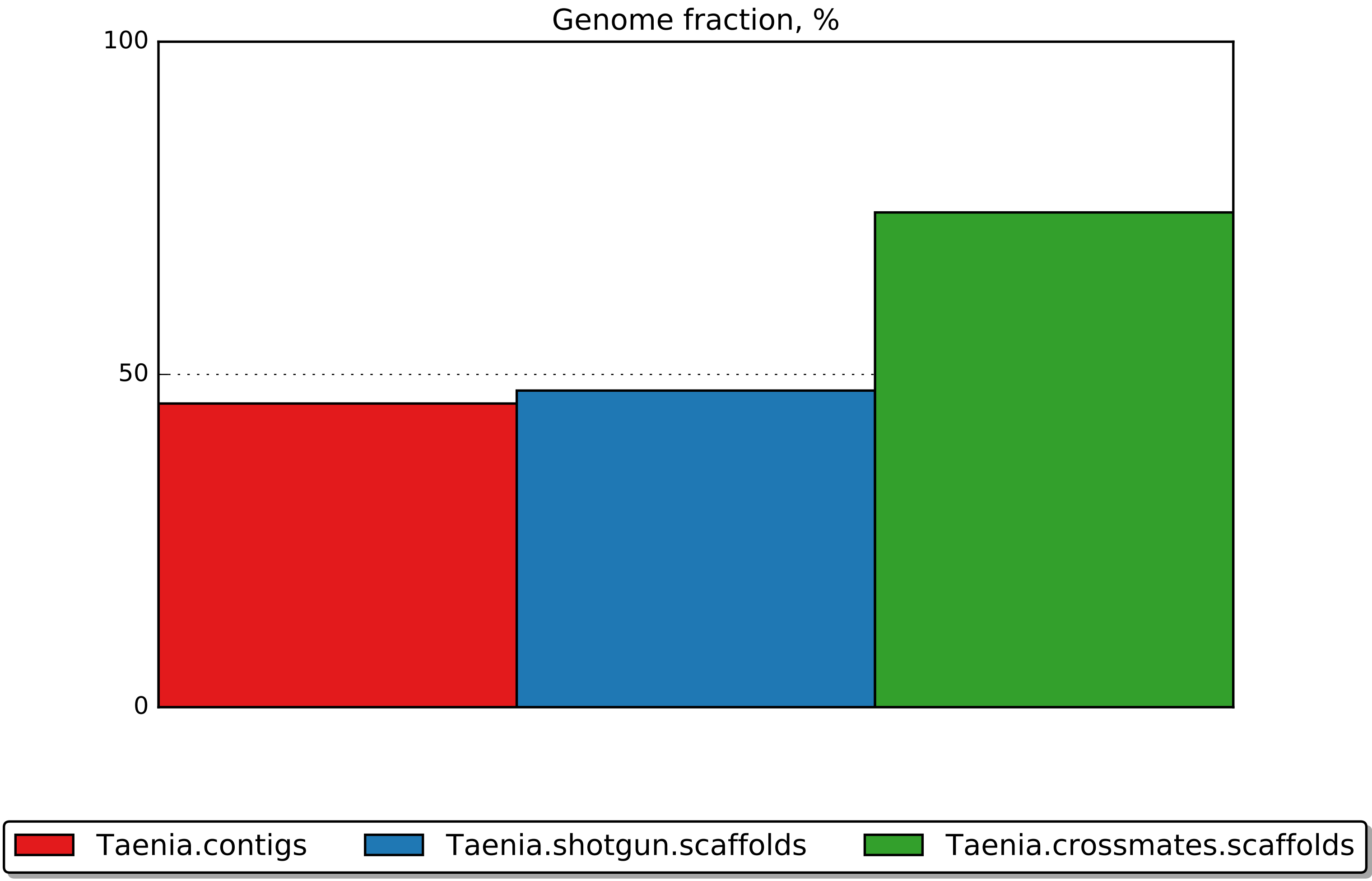

Genome fraction, %
100
50
0
Taenia.contigs
Taenia.shotgun.scaffolds
Taenia.crossmates.scaffolds
