# Supplementary material for: Improving draft genome contiguity with reference-derived in silico mate-pair libraries
Source: Gigascience. 2018 Apr 21;7(5):giy029. doi: 10.1093/gigascience/giy029 (PMC5967465; doi:10.1093/gigascience/giy029)
Supplement: Additional Files [file giy029_supp.zip › yeast_report.pdf]

Report

	scaffolds_no_mates	scaffolds_with_mates2
# contigs (>= 0 bp)	3529	3907
# contigs (>= 1000 bp)	634	174
# contigs (>= 5000 bp)	421	54
# contigs (>= 10000 bp)	326	31
# contigs (>= 25000 bp)	162	27
# contigs (>= 50000 bp)	47	26
Total length (>= 0 bp)	12002012	12259296
Total length (>= 1000 bp)	11425473	11712269
Total length (>= 5000 bp)	10937217	11433669
Total length (>= 10000 bp)	10282848	11281309
Total length (>= 25000 bp)	7424095	11210411
Total length (>= 50000 bp)	3306220	11175310
# contigs	326	31
Largest contig	122700	1026282
Total length	10282848	11281309
Reference length	12157105	12157105
GC (%)	38.23	38.23
Reference GC (%)	38.15	38.15
N50	37920	649591
NG50	32758	547102
N75	23892	419482
NG75	17086	279367
L50	90	7
LG50	116	8
L75	174	13
LG75	242	15
# misassemblies	6	49
# misassembled contigs	5	21
Misassembled contigs length	314601	10650739
# local misassemblies	7	44
# unaligned mis. contigs	0	0
# unaligned contigs	6 + 27 part	3 + 22 part
Unaligned length	205037	711625
Genome fraction (%)	82.814	86.892
Duplication ratio	1.001	1.001
# N's per 100 kbp	76.01	1752.87
# mismatches per 100 kbp	198.01	194.41
# indels per 100 kbp	17.54	9.32
Largest alignment	122541	521791
Total aligned length	10071300	10568901
NA50	36592	188364
NGA50	30975	183025
NA75	22935	102567
NGA75	16627	78650
LA50	93	19
LGA50	121	21
LA75	182	38
LGA75	254	45

All statistics are based on contigs of size >= 10000 bp, unless otherwise noted (e.g., "# contigs (>= 0 bp)" and "Total length (>= 0 bp)" include all contigs).

Misassemblies report

	scaffolds_no_mates	scaffolds_with_mates2
# misassemblies	6	49
# relocations	4	49
# translocations	2	0
# inversions	0	0
# misassembled contigs	5	21
Misassembled contigs length	314601	10650739
# local misassemblies	7	44
# misassemblies caused by fragmented reference	0	0
# unaligned mis. contigs	0	0
# mismatches	19935	20536
# indels	1766	985
# indels (<= 5 bp)	1554	725
# indels (> 5 bp)	212	260
Indels length	5377	8464

All statistics are based on contigs of size >= 10000 bp, unless otherwise noted (e.g., "# contigs (>= 0 bp)" and "Total length (>= 0 bp)" include all contigs).

Unaligned report

	scaffolds_no_mates	scaffolds_with_mates2
# fully unaligned contigs	6	3
Fully unaligned length	89558	60054
# partially unaligned contigs	27	22
Partially unaligned length	115479	651571
# N's	7816	197747

All statistics are based on contigs of size >= 10000 bp, unless otherwise noted (e.g., "# contigs (>= 0 bp)" and "Total length (>= 0 bp)" include all contigs).

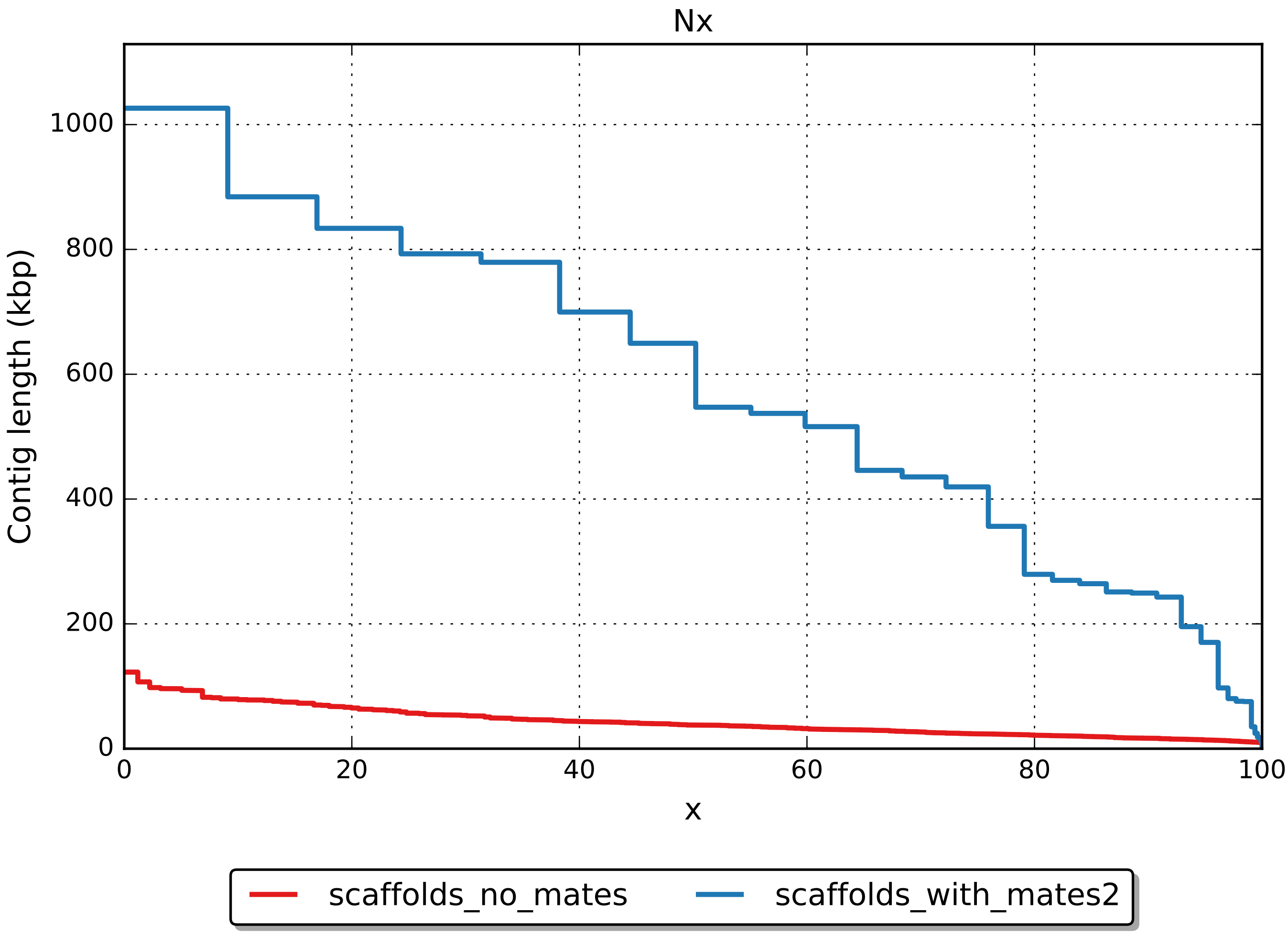

Nx
Contig length (kbp)
1000
800
600
400
200
0
0
20
40
60
80
100
x
scaffolds_no_mates
scaffolds_with_mates2

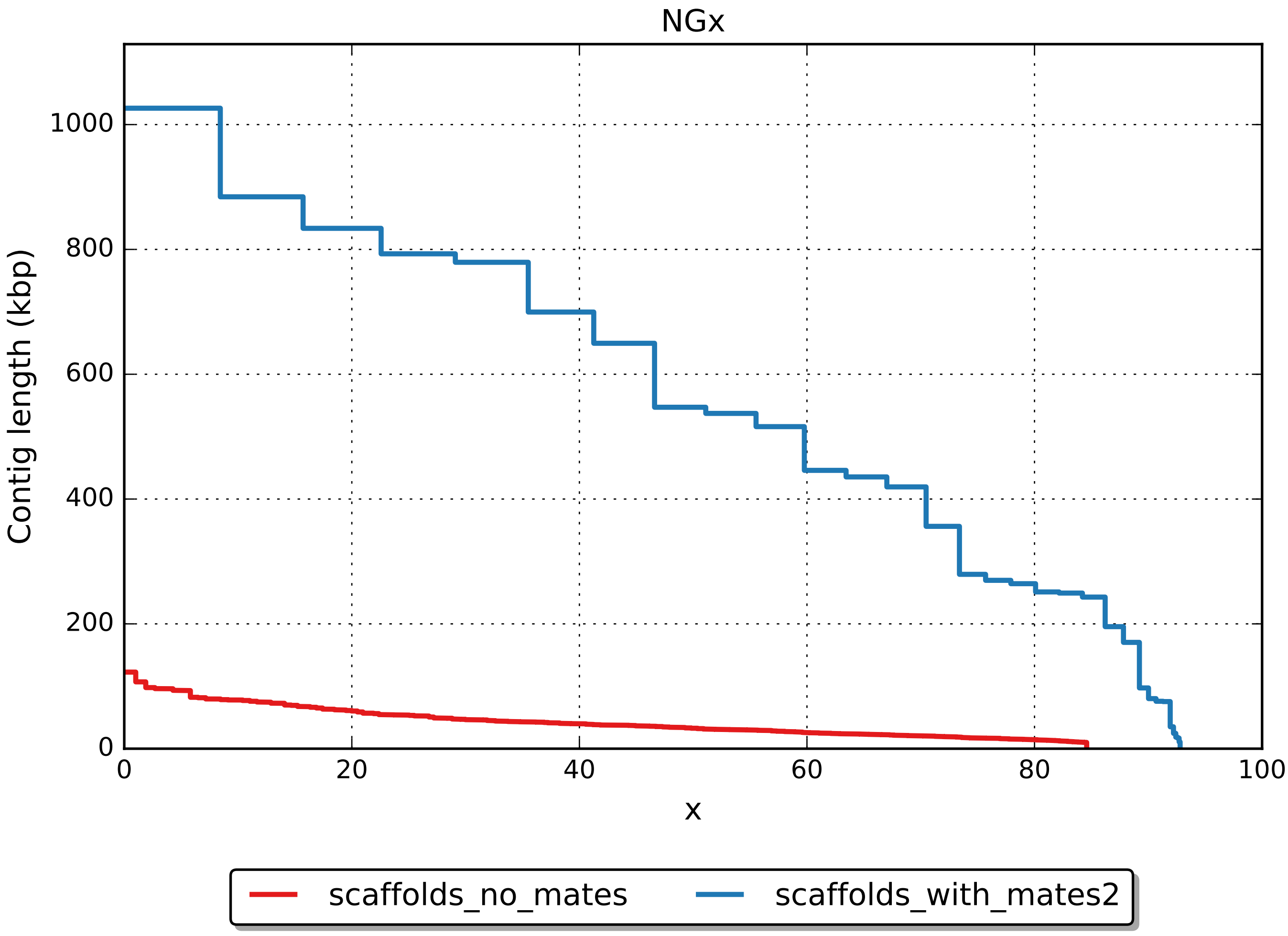
NGx
Contig length (kbp)
0
200
400
600
800
1000
0
20
40
60
80
100
x
scaffolds_no_mates
scaffolds_with_mates2

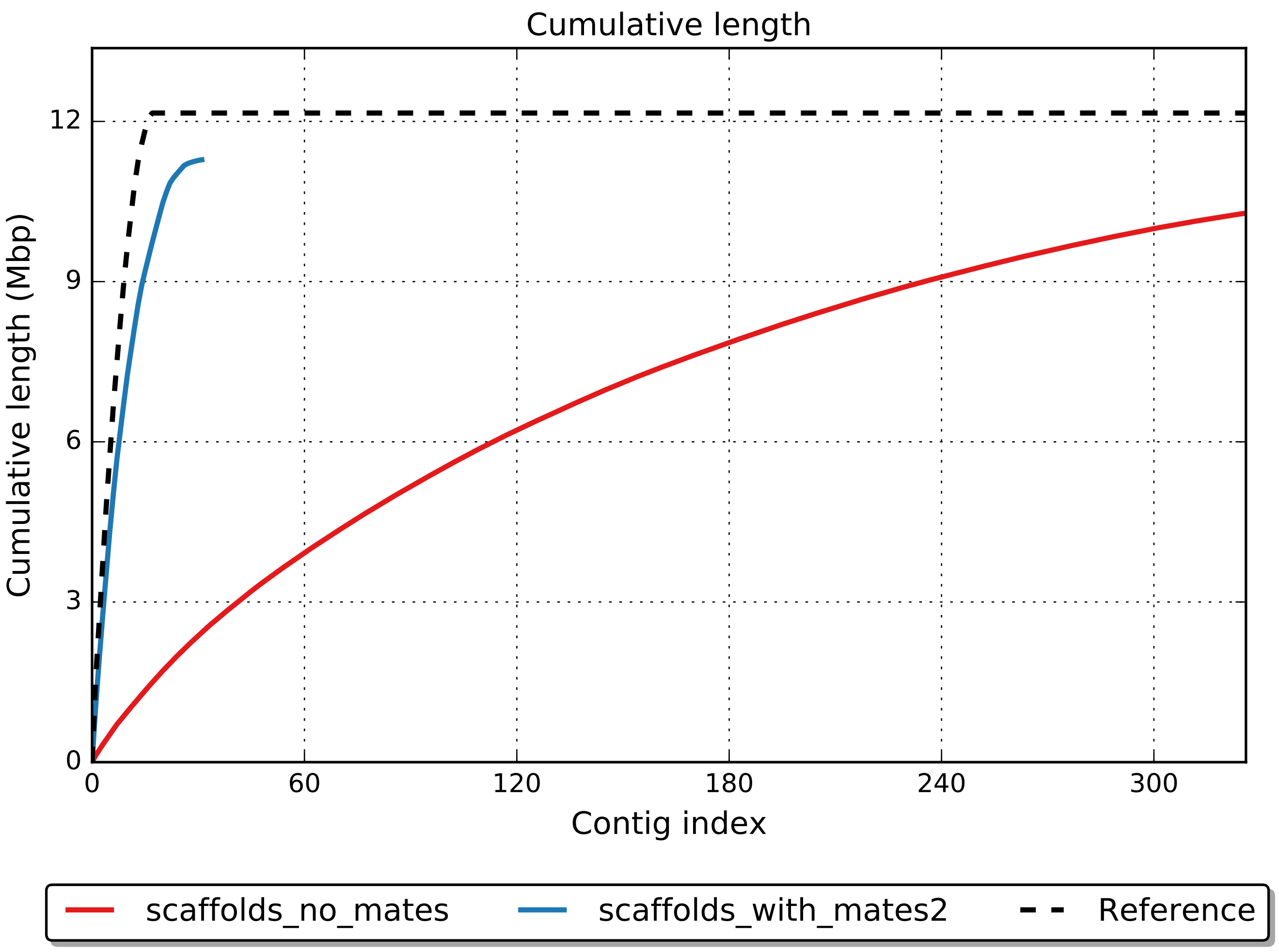
Cumulative length
Cumulative length (Mbp)
0
3
6
9
12
0
60
120
180
240
300
Contig index
scaffolds_no_mates
scaffolds_with_mates2
Reference

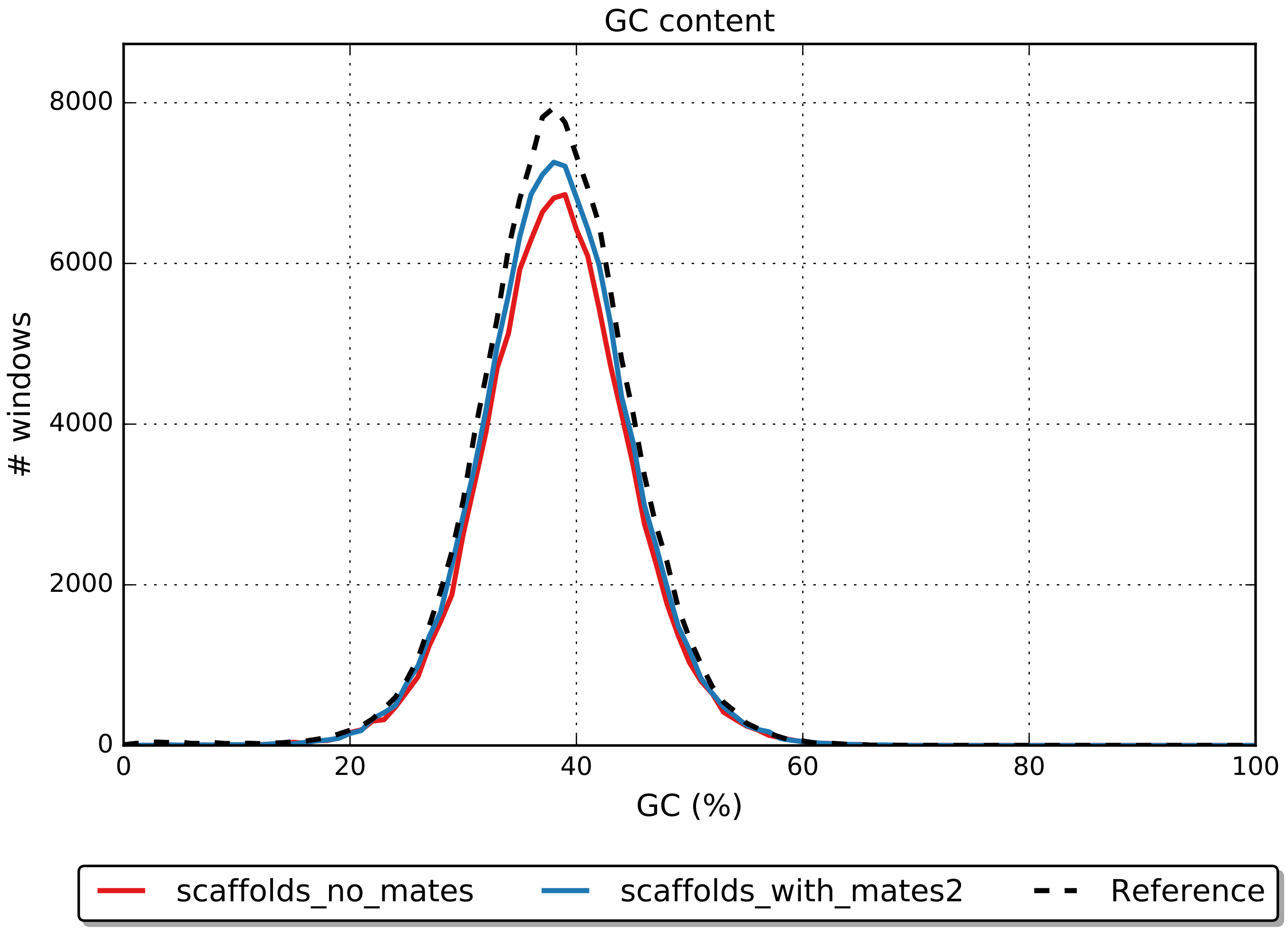
GC content
windows
0
2000
4000
6000
8000
0
20
40
60
80
100
GC (%)
scaffolds_no_mates
scaffolds_with_mates2
Reference

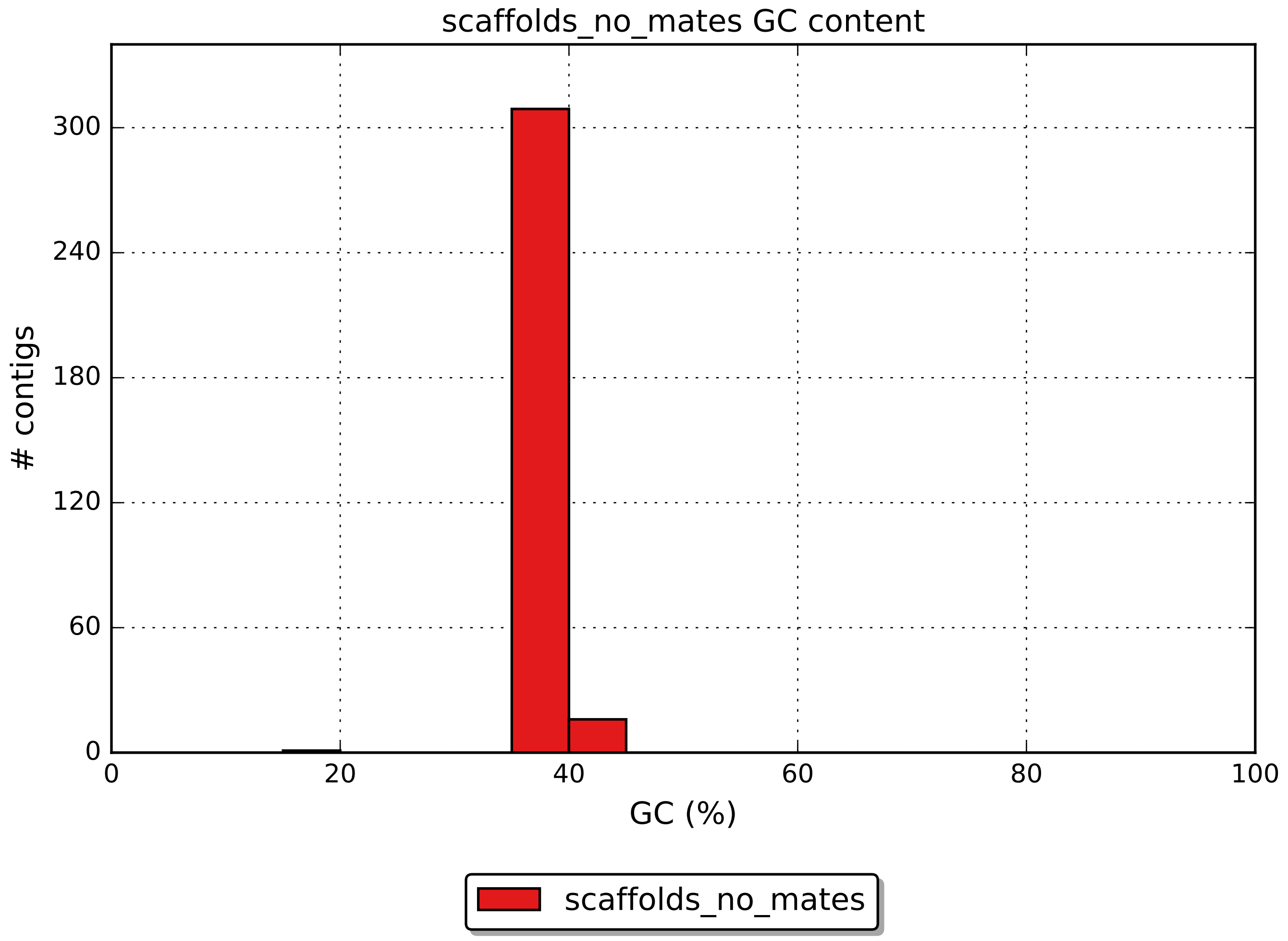
scaffolds_no_mates GC content
contigs
0
60
120
180
240
300
0
20
40
60
80
100
GC (%)
scaffolds_no_mates

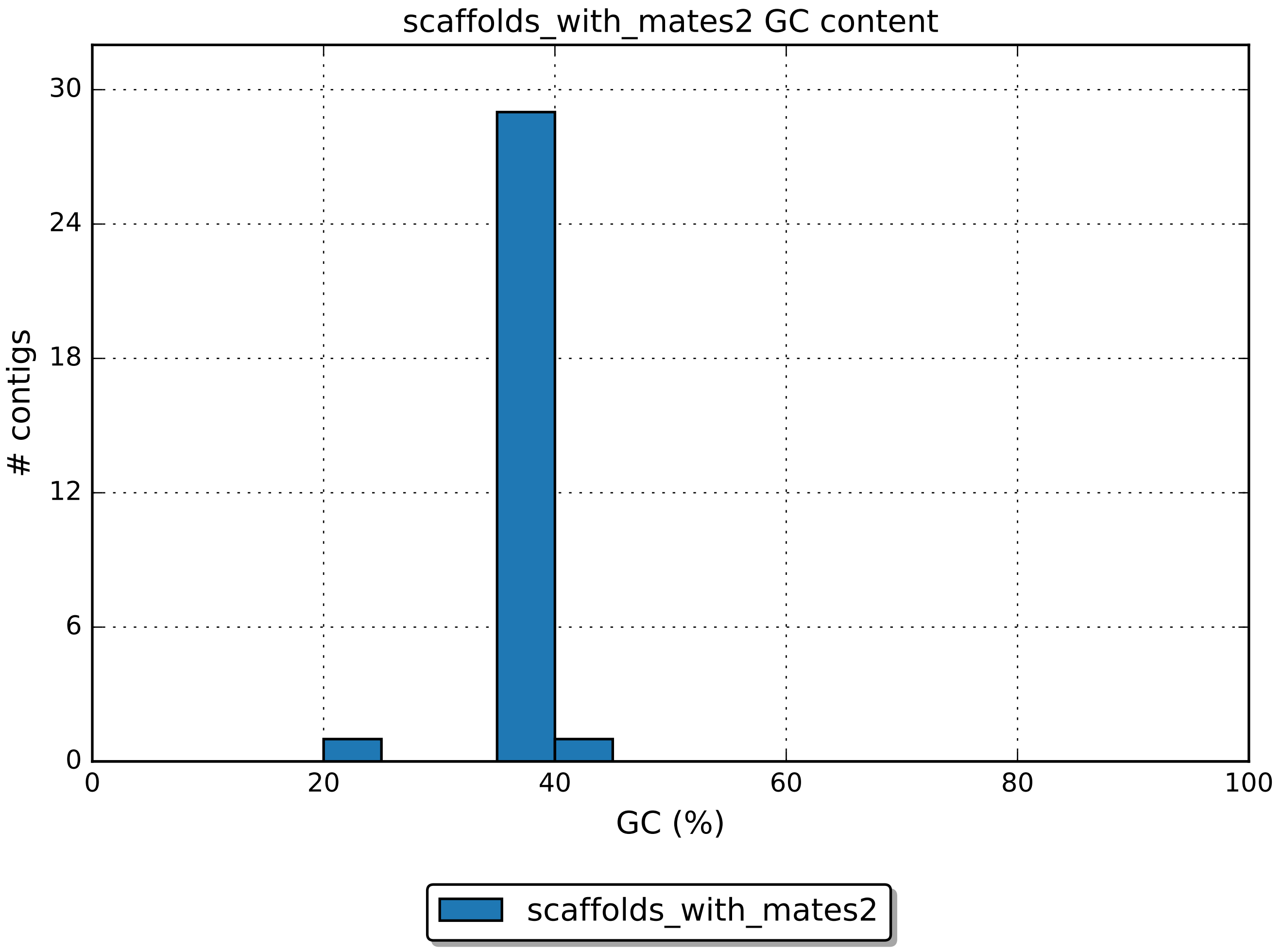
scaffolds_with_mates2 GC content
contigs
0
6
12
18
24
30
0
20
40
60
80
100
GC (%)
scaffolds_with_mates2

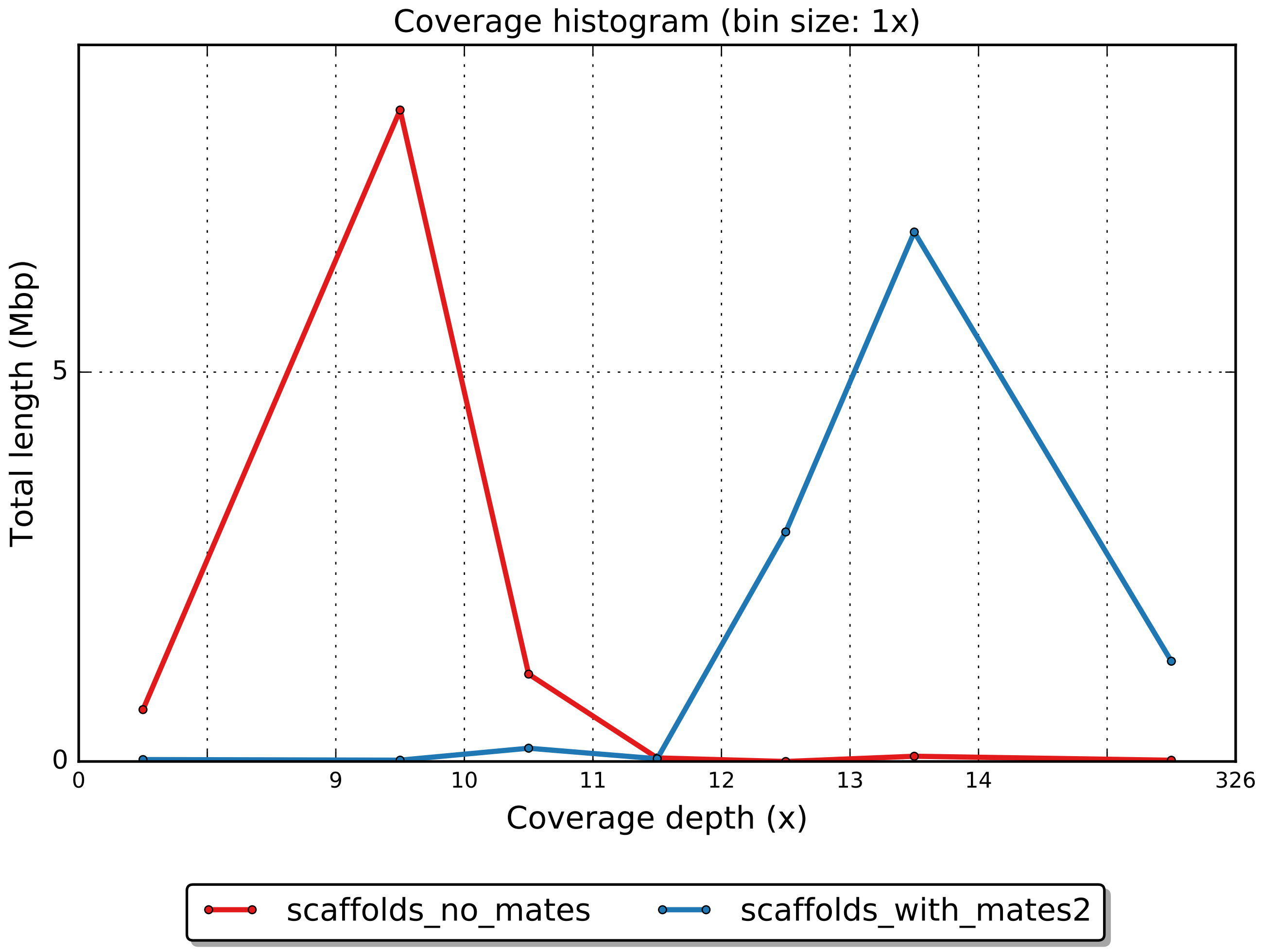
Coverage histogram (bin size: 1x)
Total length (Mbp)
5
0
0
9
10
11
12
13
14
326
Coverage depth (x)
scaffolds_no_mates
scaffolds_with_mates2

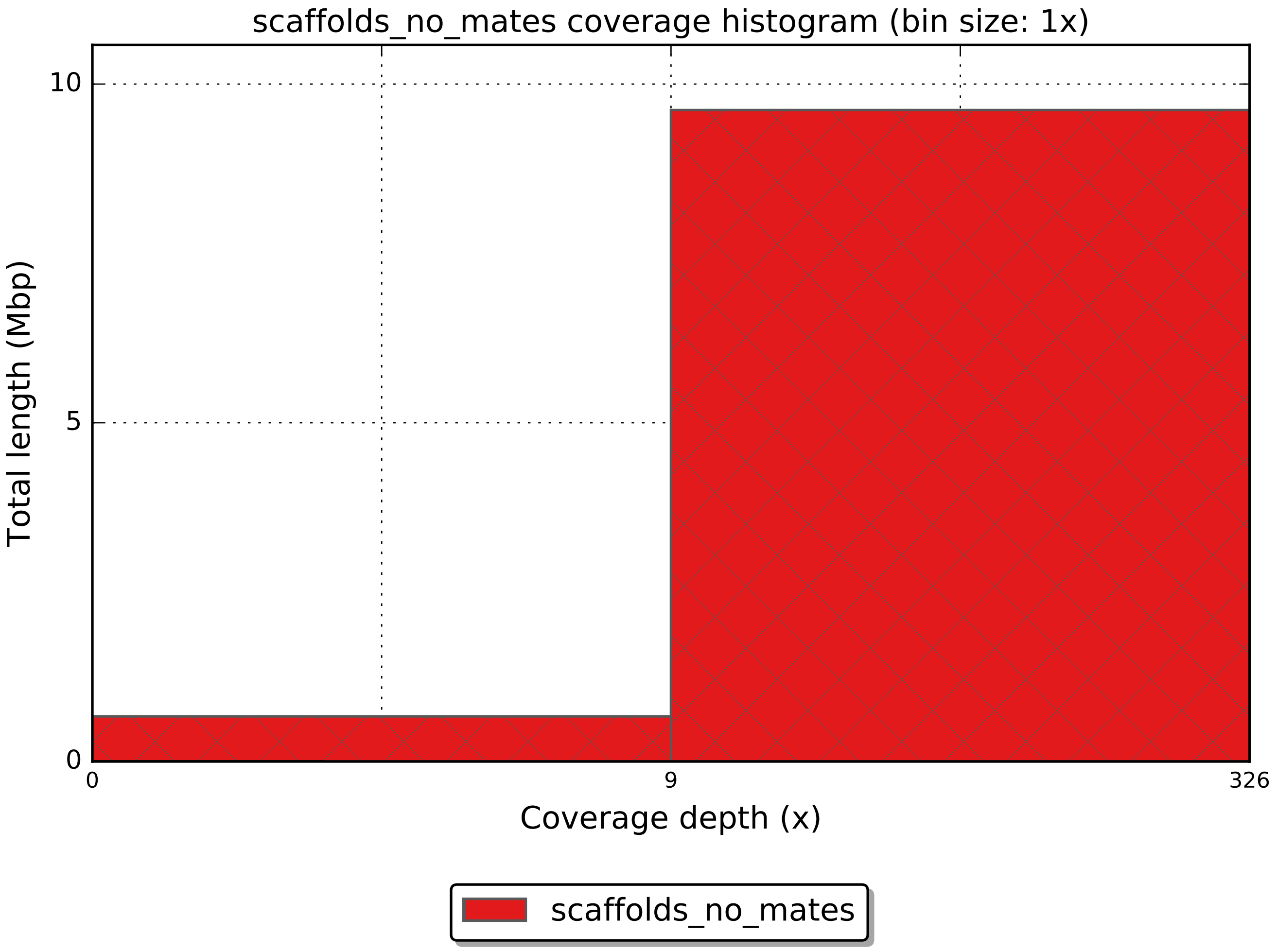
scaffolds_no_mates coverage histogram (bin size: 1x)
Total length (Mbp)
10
5
0
0
9
326
Coverage depth (x)
scaffolds_no_mates

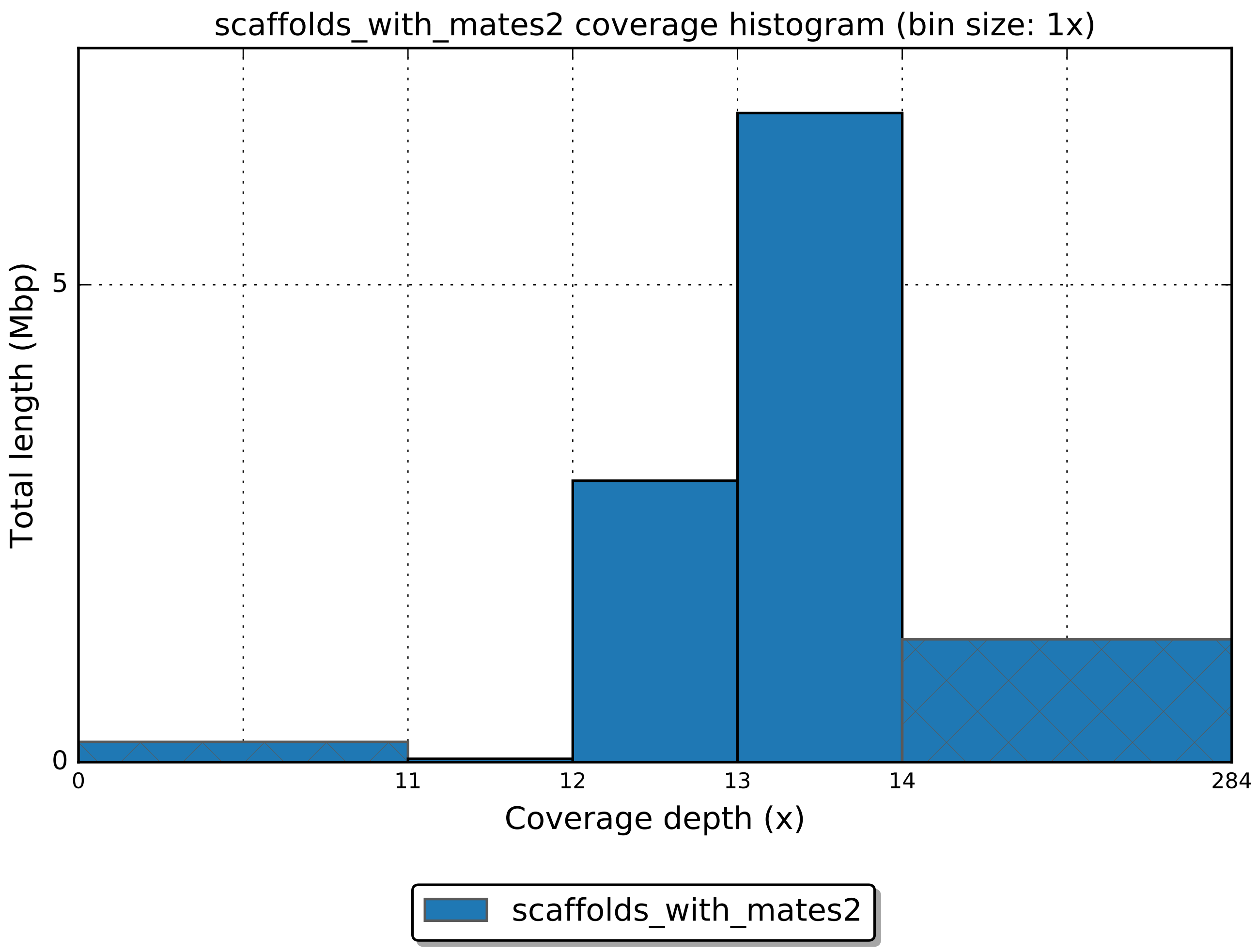
scaffolds_with_mates2 coverage histogram (bin size: 1x)
Total length (Mbp)
5
0
0
11
12
13
14
284
Coverage depth (x)
scaffolds_with_mates2

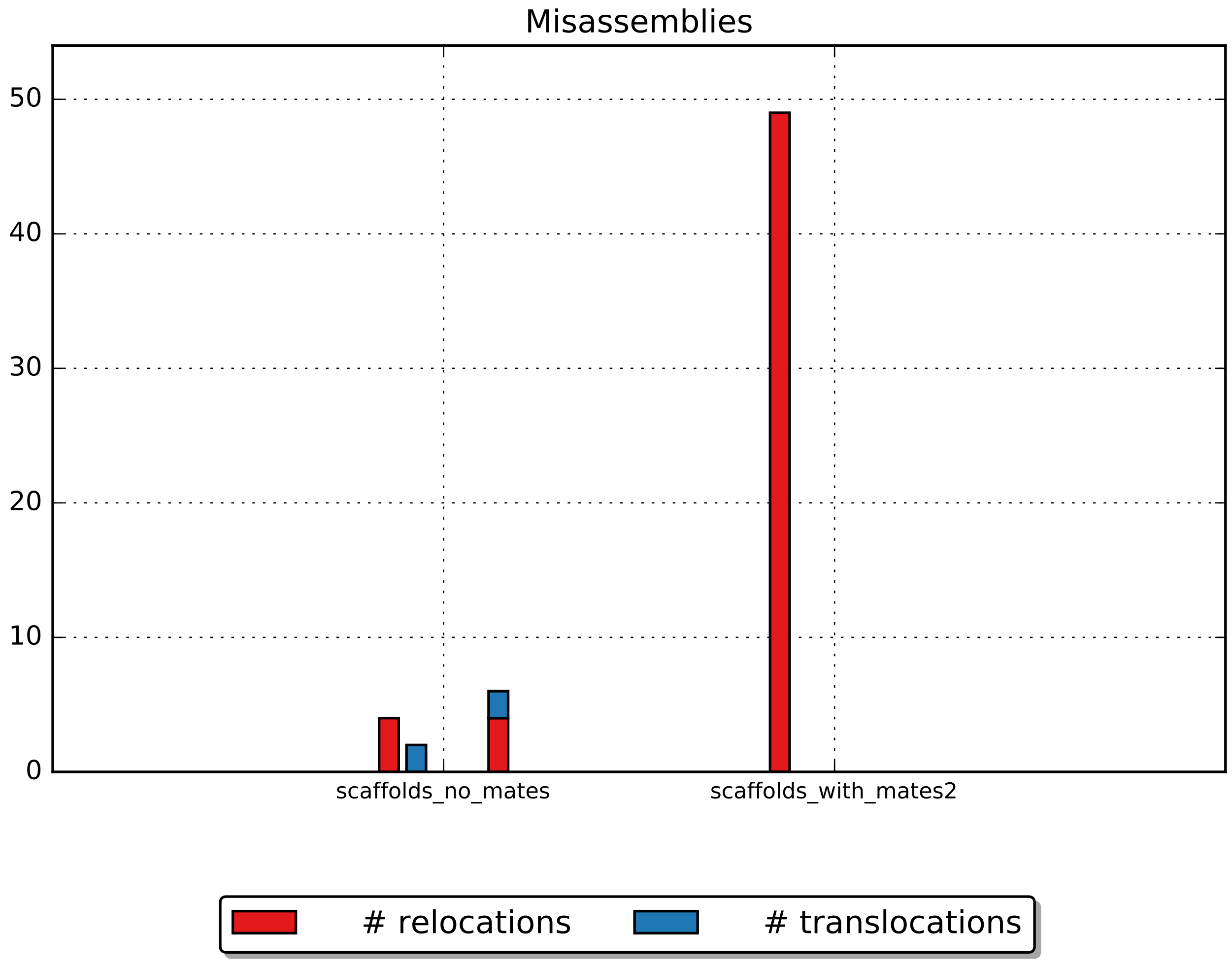
Misassemblies
50
40
30
20
10
0
scaffolds_no_mates
scaffolds_with_mates2
relocations
translocations

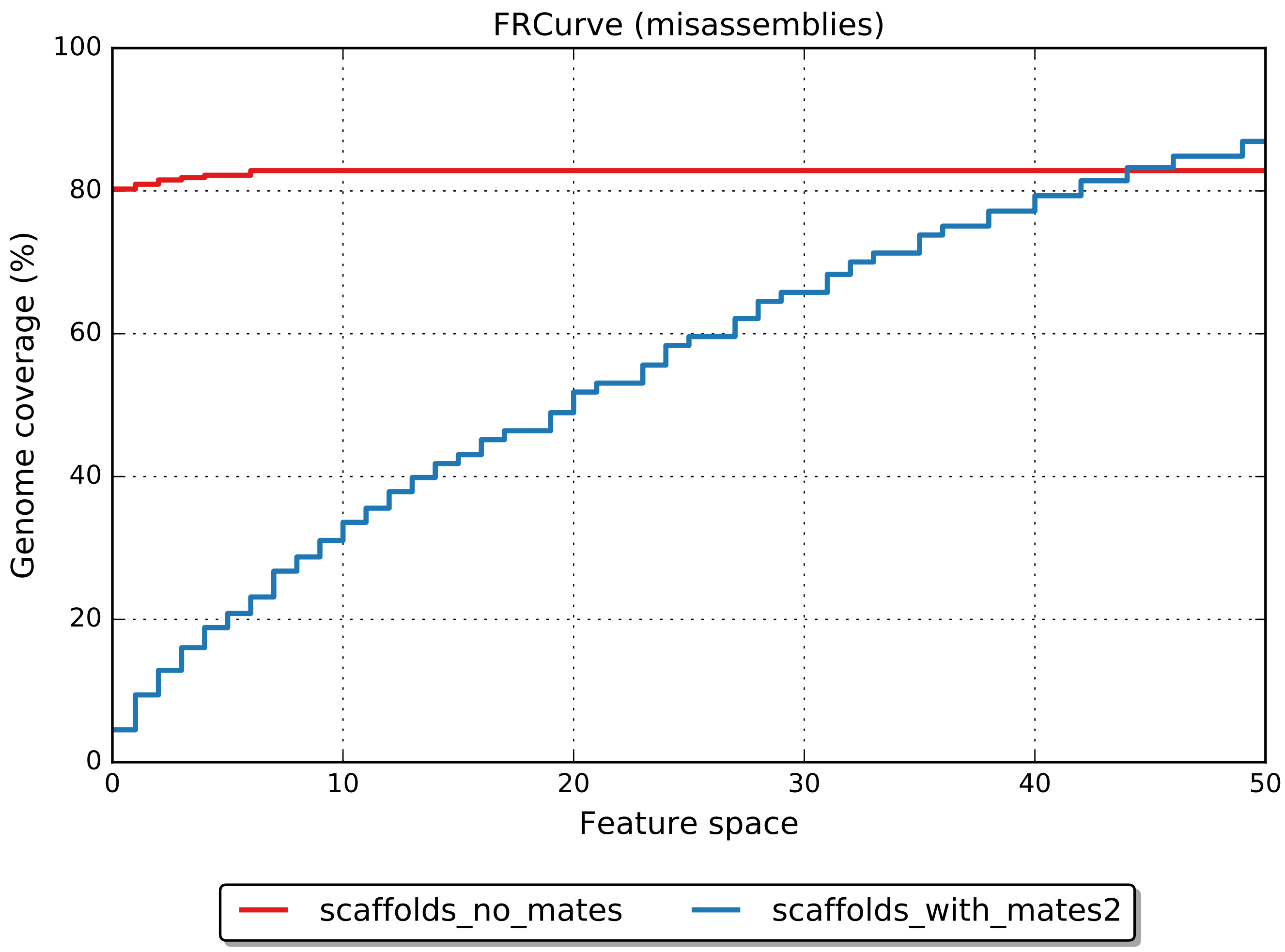
FRCurve (misassemblies)
Genome coverage (%)
Feature space
0
20
40
60
80
100
0
10
20
30
40
50
scaffolds_no_mates
scaffolds_with_mates2

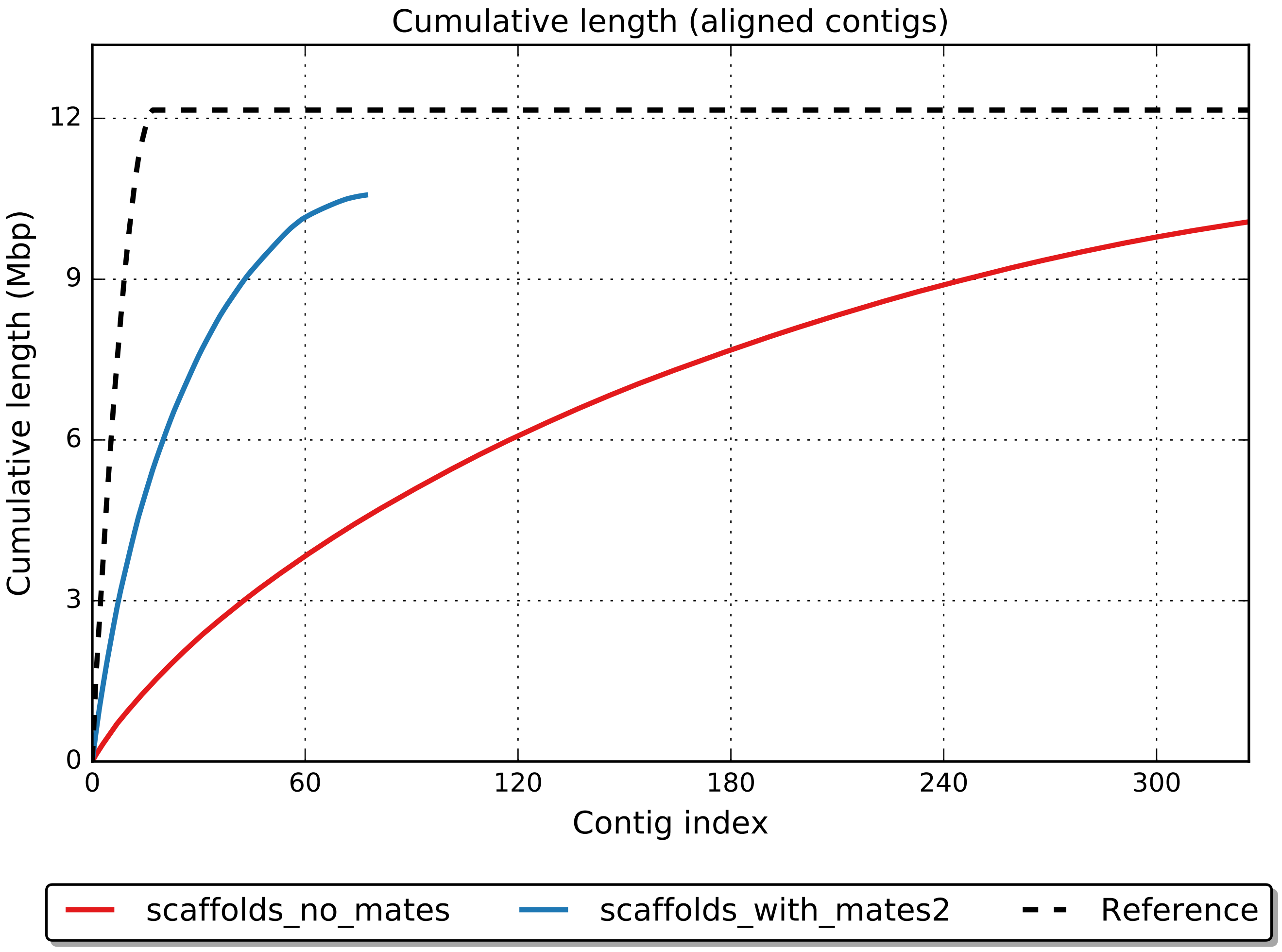

Cumulative length (aligned contigs)
Cumulative length (Mbp)
0
3
6
9
12
0
60
120
180
240
300
Contig index
scaffolds_no_mates
scaffolds_with_mates2
Reference

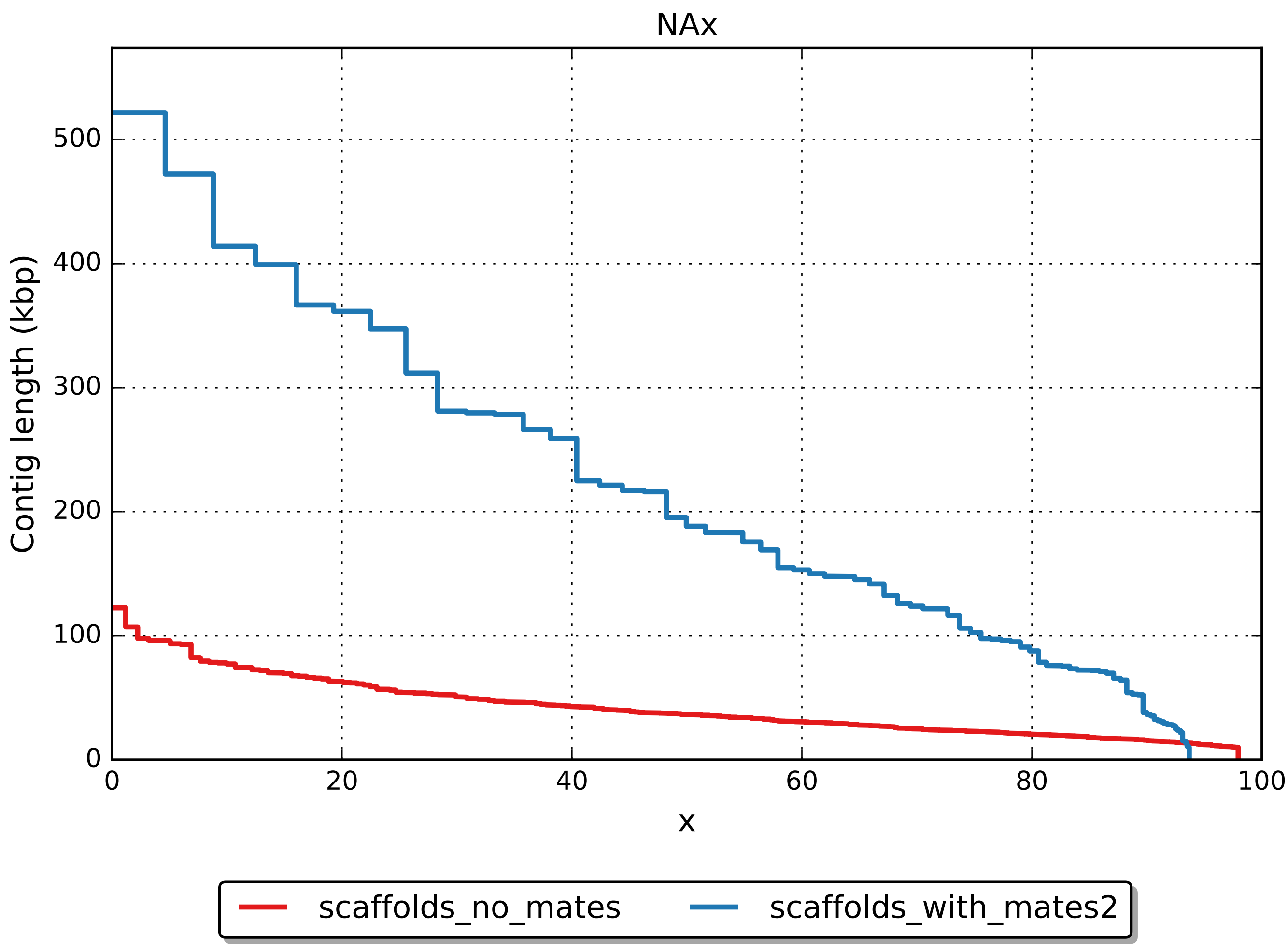
NAx
Contig length (kbp)
0
100
200
300
400
500
0
20
40
60
80
100
x
scaffolds_no_mates
scaffolds_with_mates2

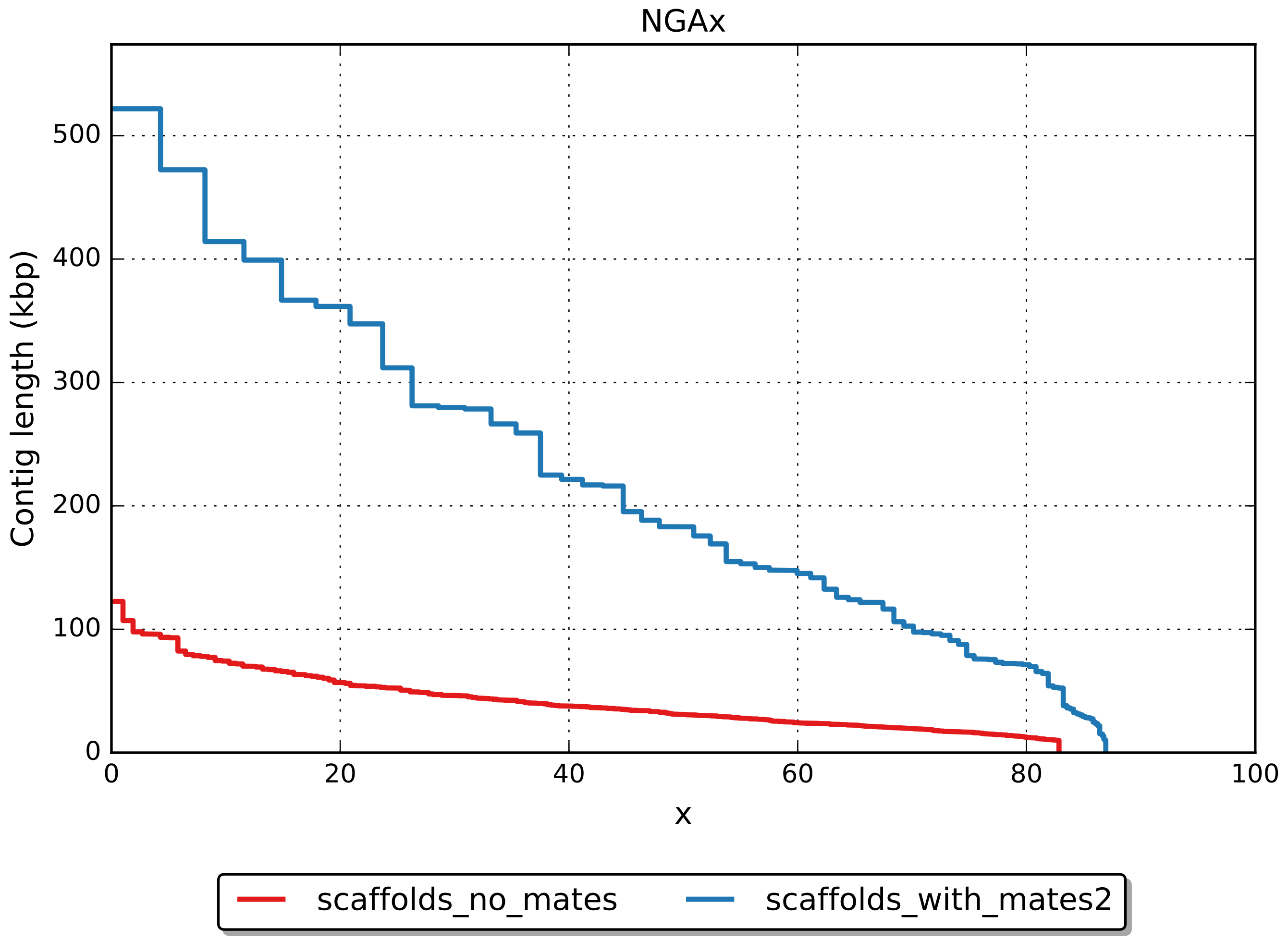

NGAx
Contig length (kbp)
0
100
200
300
400
500
0
20
40
60
80
100
x
scaffolds_no_mates
scaffolds_with_mates2

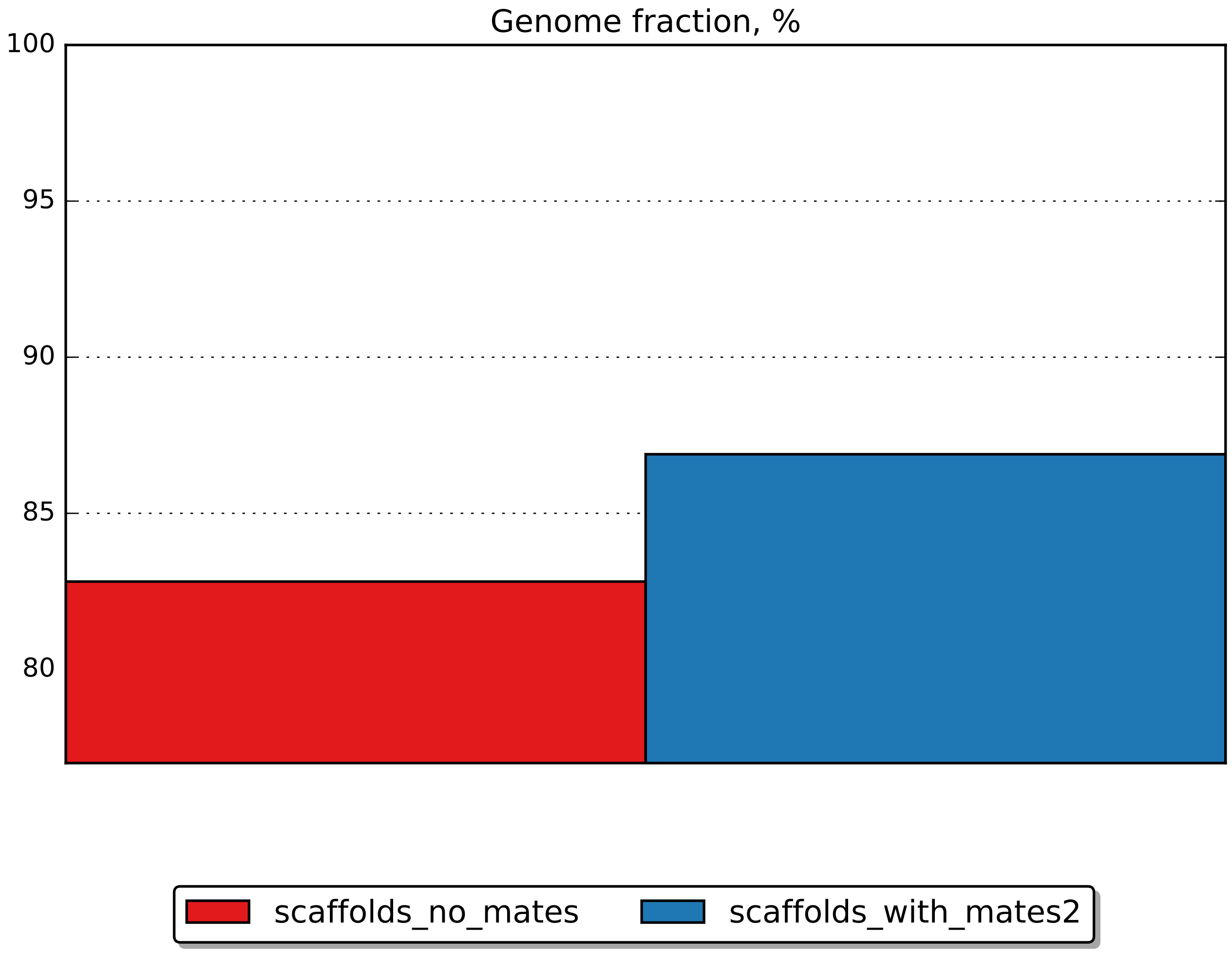
Genome fraction, %
100
95
90
85
80
scaffolds_no_mates
scaffolds_with_mates2
