# Supplementary material for: Improving draft genome contiguity with reference-derived in silico mate-pair libraries
Source: Gigascience. 2018 Apr 21;7(5):giy029. doi: 10.1093/gigascience/giy029 (PMC5967465; doi:10.1093/gigascience/giy029)
Supplement: Response_to_Reviewer_Comments_Revision_1.pdf [file giy029_response_to_reviewer_comments_revision_1.pdf]

Author's Response To Reviewer Comments

Please find Respond to reviewers in the Personal letter. Thank you.
