# Supplementary material for: Improving draft genome contiguity with reference-derived in silico mate-pair libraries
Source: Gigascience. 2018 Apr 21;7(5):giy029. doi: 10.1093/gigascience/giy029 (PMC5967465; doi:10.1093/gigascience/giy029)
Supplement: Reviewer_1_Report_(Original_Submission) -- Kristoffer Sahlin — 12 May 2017 Reviewed [file giy029_reviewer_1_report_(original_submission).pdf]

**Reviewer Report**

**Title:** Improving draft genome contiguity with reference-derived in silico mate-pair libraries

**Version:** Original Submission **Date:** 12 May 2017

**Reviewer name:** Kristoffer Sahlin

**Reviewer Comments to Author:**

SUMMARY

The authors present a pipeline to generate in silico mate pair (MP) libraries of a query organism using a related genome sequence as reference. They show that their pipeline constructs libraries that, when used by the assembler SOAPdenovo, significantly improves various assembly metrics. The genome assembly approach used in this paper is to (1) generate a set of contigs using Illumina reads from a query genome, (2) Scaffold the contigs using their in silico generated MP libraries (3) fill the gaps with a gap closer. Several details on how the in silico MP libraries are generated are left out (see comments below). In general: Illumina reads from the query genome are mapped to a related reference genome. A artificial reference sequence in fastq format is constructed by consensus calling over each base pair based on the illumina query reads. The fastq quality values are determined by the coverage over each position. MP reads in fastq format are generated by simulating a position and an insert size on the artificial reference.

The pipeline for creating in silico mate pair libraries is easy to install and the code repository is well documented. The described pipeline seems to be novel and I find the assembly strategy appealing given that one is willing to accept the obvious risk of introducing errors from structural differences between query and reference. The authors show that not only the quantitative metrics improve with this method but also the quality in terms of recovered genes — which increases significantly. Also, the results show that scaffolding of the Aye-Aye genome is more conservative with a more distant reference (human) compared to a closer one (Lemur). These are both sound attributes of the method.

The authors do not directly evaluate assembly errors introduced by this approach — which is the obvious pitfall to point out. After some consideration, my opinion is that it's not crucial to evaluate assembly errors in this work. My reasons for this is that any evaluation of reasonable magnitude I can think of would not be directly capture flaws in the main idea, which is the construction of in silico MP reads. For instance, the pipeline is not tied to any specific assembler, thus assembly errors may vary with the assembly method using the in silico libraries. Also, it is relatively clear from context that this is an obvious drawback of such a method — although this should at least be explicitly mentioned in the manuscript. I would therefore leave any evaluation of assembly errors as discretionary. Below are some issues I believe need to be addressed.

MINOR

* Several details of the pipeline needs to be described:
1. How are insert sizes and mate pair positions sampled from the reference? I assume at random by looking at seq-frag source, this needs to be described.
2. What happens if there are no reads in a region where a MP is sampled? will it be discarded, consists of only N's, or the sequence of the reference is taken? Please describe.
3. How are fastq qualities chosen in the reads? Specifically: "Additionally, because of the consensus calling from the mapped reads, allelic differences will be converted to ambiguous bases in the scaffolding libraries", what are the fastq qualities of those bases?
3. Which reads were used in the gap filling step, only illumina query or the in silico MP reads as well?
4. What alignment parameters were used for BWA to align illumina to reference?

* Discussion: "This is an essentially novel approach since other chromosome scaffolders, such as Chromosomer [17] , MeDuSa [28] , and AlignGraph [29] , exploit distance information from genomes of closely related organisms to order and extend scaffold or contigs after the de novo assembly process." — Generating in silico MP libraries is on a high level the same as "exploit distance information", so this novelty motivation and and distinction to other methods needs to be refined.

* Provide runtime of the mate pair generation experiments.

* Methods: "Assembly quality statistics were measured with Quast" A bit misleading if a reference is not used to calculate _quality_ of assembly such as misassembles. Use other term such as quantity metrics or listing the metrics explicitly.

* Discussion: "First, extra-long-range scaffolding information can be easily obtained, since our tool has no maximum insert size and the upper limit of insert size remains to be explored in relation to syntenic conservation" — Manuscript text says that mate pair libraries of any size can be generated but the pipeline script output options up to 200000. Fix discrepancy, e.g., by updating the code documentation that values are flexible and can easily be adjusted in the code. (Also, documentation says 200,000 kbp. —> 200kbp)

* Explain "DauMad-1.0" in figure 2B.

DISCRETIONARY

* How about comparing your assembly to the current chimp reference? For instance comparing the scaffolds (i.e., "Chimp with human") and the original contigs (i.e., "Chimp") using QUAST.

OTHER COMMENTS

The idea of using information of multiple reference sequences as done in MeDuSa or Multi-CAR might translate to a good extension for this approach as well, especially when multiple high quality references become available. Since coverage from each reference can be controlled in the in silico generation of mate pairs in cross-species-scaffolding pipeline, one possibility is to generate each mate pair library as a mixture of sampling from each artificial reference with, e.g., equal abundance.

The scaffolding tool (in this case SOAPdenovo) will in that case hopefully have more choices to select a correct join of contigs as it is more likely that at least one of the references are similar in any given region. This might conceptually remove some of the structural errors introduced by using a single reference sequence. The downside would be more "noisy" input to a typical genome scaffolder — with the "noise" being inconsistencies between read pairs within a library as they come from different references. This could potentially be handled with a scaffolder designed to take this specific input instead of a typical genome scaffolder.

**Level of Interest**

Please indicate how interesting you found the manuscript: An article of importance in its field

**Quality of Written English**

Please indicate the quality of language in the manuscript: Acceptable

**Declaration of Competing Interests**

Please complete a declaration of competing interests, considering the following questions:

- Have you in the past five years received reimbursements, fees, funding, or salary from an organisation that may in any way gain or lose financially from the publication of this manuscript, either now or in the future?
- Do you hold any stocks or shares in an organisation that may in any way gain or lose financially from the publication of this manuscript, either now or in the future?
- Do you hold or are you currently applying for any patents relating to the content of the manuscript?
- Have you received reimbursements, fees, funding, or salary from an organization that holds or has applied for patents relating to the content of the manuscript?
- Do you have any other financial competing interests?
- Do you have any non-financial competing interests in relation to this paper?

If you can answer no to all of the above, write 'I declare that I have no competing interests' below. If your reply is yes to any, please give details below.

I declare that I have no competing interests.

I agree to the open peer review policy of the journal. I understand that my name will be included on my report to the authors and, if the manuscript is accepted for publication, my named report including any attachments I upload will be posted on the website along with the authors' responses. I agree for my report to be made available under an Open Access Creative Commons CC-BY license (http://creativecommons.org/licenses/by/4.0/). I understand that any comments which I do not wish to be included in my named report can be included as confidential comments to the editors, which will not be published.

I agree to the open peer review policy of the journal

To further support our reviewers, we have joined with Publons, where you can gain additional credit to further highlight your hard work (see: https://publons.com/journal/530/gigascience). On publication of this paper, your review will be automatically added to Publons, you can then choose whether or not to claim your Publons credit. I understand this statement.

Yes
