# Supplementary material for: Improving draft genome contiguity with reference-derived in silico mate-pair libraries
Source: Gigascience. 2018 Apr 21;7(5):giy029. doi: 10.1093/gigascience/giy029 (PMC5967465; doi:10.1093/gigascience/giy029)
Supplement: Reviewer_1_Report_(Revision_1) -- Kristoffer Sahlin — 22 Aug 2017 Reviewed [file giy029_reviewer_1_report_(revision_1).pdf]

Reviewer Report

Title: Improving draft genome contiguity with reference-derived in silico mate-pair libraries

Version: Revision 1 **Date:** 22 Aug 2017

Reviewer name: Kristoffer Sahlin

Reviewer Comments to Author:

The authors has addressed most of the points and I'm in general satisfied with the answers. I do have some significant remaining issues.

1. Runtime should be measured on the entire pipeline since a user have to run all three steps (alignment, pileup and seq-frag) to obtain the in silico mate pairs (my assumption at this point is especially that pileup is a costly way to iterate over alignments). I would advise the authors to provide the runtime of the three different steps separately (aligning, pileup, and seq-frag).

2. Provide the misassembly metrics from QUAST. The authors only present the QUAST quantity metrics positively influenced by increased contiguity, but leaves the misassembly stats out. In my previous review I argued that I did not find an _evaluation_ of assembly errors and types necessary because this will depend on differences between reference and query as well as assembly algorithm. However, simply providing numbers such as adjusted N50 and the larger misassembly types for the applicable datasets (where high quality reference exists, e.g., chimp, yeast) is useful and helps further development in this direction. In fact, the yeast data set will be very informative here as the reference and query are the same, thus removing the factor of structural differences between genomes.

3. "While in silico mate-pairs introduces minimal errors since position and arrangement is suggested and validated by shotgun data during the de novo assembly process, they cannot fully replace ..." Beginning of sentence a bit misleading. "introduces minimal errors" in this context can be interpreted as introduces errors in the assembly. MP has errors such as PE contamination and chimera, in silico generated MP on the other hand has "structural errors" corresponding to the differences between reference and query. I suggest something more precise in the style of "While in silico MP preparation does not introduce any [sequencing/library preparation] errors such as PE contamination, chimera,... they cannot replace"

Discretionary
* "An obvious drawback of this approach may be the introduction of assembly chimaeras.." —> "A drawback of this approach may be the introduction of assembly chimaeras". Although I used "obvious" three times in my previous review, I don't think this belongs in a manuscript.

Level of Interest

Please indicate how interesting you found the manuscript: An article whose findings are important to those with closely related research interests

Quality of Written English

Please indicate the quality of language in the manuscript: Acceptable

Declaration of Competing Interests

Please complete a declaration of competing interests, considering the following questions:

- Have you in the past five years received reimbursements, fees, funding, or salary from an organisation that may in any way gain or lose financially from the publication of this manuscript, either now or in the future?
- Do you hold any stocks or shares in an organisation that may in any way gain or lose financially from the publication of this manuscript, either now or in the future?
- Do you hold or are you currently applying for any patents relating to the content of the manuscript?
- Have you received reimbursements, fees, funding, or salary from an organization that holds or has applied for patents relating to the content of the manuscript?
- Do you have any other financial competing interests?
- Do you have any non-financial competing interests in relation to this paper?

If you can answer no to all of the above, write 'I declare that I have no competing interests' below. If your reply is yes to any, please give details below.

I declare that I have no competing interests

I agree to the open peer review policy of the journal. I understand that my name will be included on my report to the authors and, if the manuscript is accepted for publication, my named report including any attachments I upload will be posted on the website along with the authors' responses. I agree for my report to be made available under an Open Access Creative Commons CC-BY license (http://creativecommons.org/licenses/by/4.0/). I understand that any comments which I do not wish to be included in my named report can be included as confidential comments to the editors, which will not be published.

I agree to the open peer review policy of the journal

To further support our reviewers, we have joined with Publons, where you can gain additional credit to further highlight your hard work (see: https://publons.com/journal/530/gigascience). On publication of this paper, your review will be automatically added to Publons, you can then choose whether or not to claim your Publons credit. I understand this statement.

Yes
